# Integrative taxonomy and species distribution models of the genus *Diamesus* Hope, 1840 (Coleoptera: Staphylinidae: Silphinae)

**DOI:** 10.1038/s41598-023-30019-x

**Published:** 2023-02-23

**Authors:** Jan Růžička, Pavel Jakubec, Karolina Mahlerová, Hana Šípková, Masaaki Nishikawa

**Affiliations:** 1 Department of Ecology, Faculty of Environmental Sciences, Czech University of Life Sciences Prague, Kamýcká 129, 165 00 Prague-Suchdol, Czech Republic; 2 Kashiwagaya 1112-16, Ebina, 243-0402 Japan

**Keywords:** Entomology, Taxonomy, Phylogenetics

## Abstract

Integrative taxonomy of *Diamesus* Hope, 1840 (Coleoptera: Silphinae) is presented. Adults of *D. bimaculatus* Portevin, 1914 (endemic to Taiwan) and *D. osculans* (Vigors, 1825) (widely distributed from northern India to Australia) are redescribed, keyed and figured, including characters of the male and female genitalia of both species. Variation in elytral maculation in *D. osculans* is discussed and illustrated. The absence of diagnostic differences of *D*. *osculans* var. *reductus* Pic, 1917 from *D. osculans* is discussed, and the former name is confirmed as a junior subjective synonym of *D. osculans*. Types of all three names available were studied; a lectotype and paralectotypes are designated for the name *D. osculans* var. *bimaculatus* Portevin, 1914. Molecular phylogenetic analysis confirms the genus *Diamesus* is sister group to the genus *Necrodes* Leach, 1815, and *D. osculans* and *D. bimaculatus* are two, well supported clades. Detailed data on the distribution of *D. bimaculatus* and *D. osculans* are presented and mapped. Species distribution models for both species were created and interpreted. *Diamesus osculans* is reported for the first time from India: Uttarakhand, China: Anhui, Hainan, Hunan, Jiangxi, Shaanxi and Zhejiang Provinces, and Australia: Victoria; it is also recently confirmed from Taiwan, being sympatric in distribution there with *D. bimaculatus*. Available data on the ecology and seasonality of both species of *Diamesus* are also discussed.

## Introduction

The subfamily Silphinae contains beetles with wide range of ecological and food strategies, which probably originated from necrophagy^1^. The necrophagous species belonging to Silphinae became the focus of many ecological studies in recent years, and as a result, it was found that different species react differently to the same stimuli. For example, values of lower developmental threshold (LDT) and accumulated degree days (ADD) can differ between species of the same genus^2–4^. Therefore, correct species delimitation is extremely important for ecology and many other fields of science including forensic entomology, which use LDT and ADD for predictions such as post-mortem interval (PMImin) or time of colonization (ToC)^4^. In this paper, we delimit the species of the genus *Diamesus* Hope, 1840, which can be further used for ToC prediction. Vigors^5^ described from “Indiâ Orientali” the species *Necrodes osculans*, and discussed its seemingly transitional evolutionary position between two genera of carrion beetles, *Necrodes* Leach, 1815 and *Nicrophorus* Fabricius, 1775. Hope^6^ created a separate genus *Diamesus* for this species. Portevin^7^ described a series of specimens from Taiwan: Kosempo with only bimaculate elytra as *D. osculans* var. *bimaculatus*. Later, Portevin^8^ elevated this variety to species status, based on its reduced elytral maculation and its shortened median crest of the pronotum and scutellum in comparison with *D. osculans*. Pic^9^ described a single specimen from Sumatra with reduced red elytral maculation as *D. osculans* var. *reductus*. Portevin^10^ provided a detailed redescription of both *D. bimaculatus* and *D. osculans*. Arnett^11^ also included a description and illustration of the female genitalia of *D. osculans*, and mentioned the virtual absence of differentiating characters between *D. osculans* and *D. bimaculatus* other than the reduced maculation in the latter. Peck^12^ reviewed the carrion beetles of Australia, and mapped the distribution of *D. osculans* in the Australian Region. King et al.^1^ studied the phylogenetic placement of *Diamesus* as sister to *Necrodes* Leach, 1815, and published sequences for Australian carrion beetles including *D. osculans*. Růžička and Schneider^13^ and Růžička^14^ reviewed the general distribution of both species of *Diamesus* through the Palaearctic Region. Zhang et al.^15^ published a complete mitochondrial genome of *D. osculans*.

Carrion beetles were traditionally classified as a separate family Silphidae, closely related to the megadiverse family Staphylinidae^16,17^. Recent molecular phylogenetic studies placed carrion beetles as an internal lineague within Staphylinidae^18–20^. Most recently, Cai et al.^21^ formally downgraded carrion beetles as a subfamily of Staphylinidae. This is also followed by Newton^22^. Traditionally, within Silphidae, two subfamilies were recognised, Nicrophorinae and Silphinae, the latter sometimes further divided into two tribes, Necrodini and Silphini^17^. Consequently, to the changes proposed by Cai et al.^21^, these lineagues should be downgraded to two tribes, Nicrophorini and Silphini, the latter with two subtribes, Necrodina and Silphina. This classification is followed further in the text.

*Diamesus* and *Necrodes* (the latter with three species, two Palaearctic and one Nearctic in distribution) were traditionally classified in the subtribe Necrodina, with other Silphini in Silphina Portevin^10^. This classification was followed by Peck^12^ and King et al.^1^. However, a preliminary phylogenetic analysis of Dobler and Müller^23^, which was later corroborated by King et al.^1^ showed that the Silphina is paraphyletic with respect to Necrodina. Also, Newton and Thayer^24^ listed Necrodina as a synonym of Silphini. This classification is followed in most recent papers, including Sikes^17,25^, Růžička^14^ and Newton^22^.

**Figure 1:**
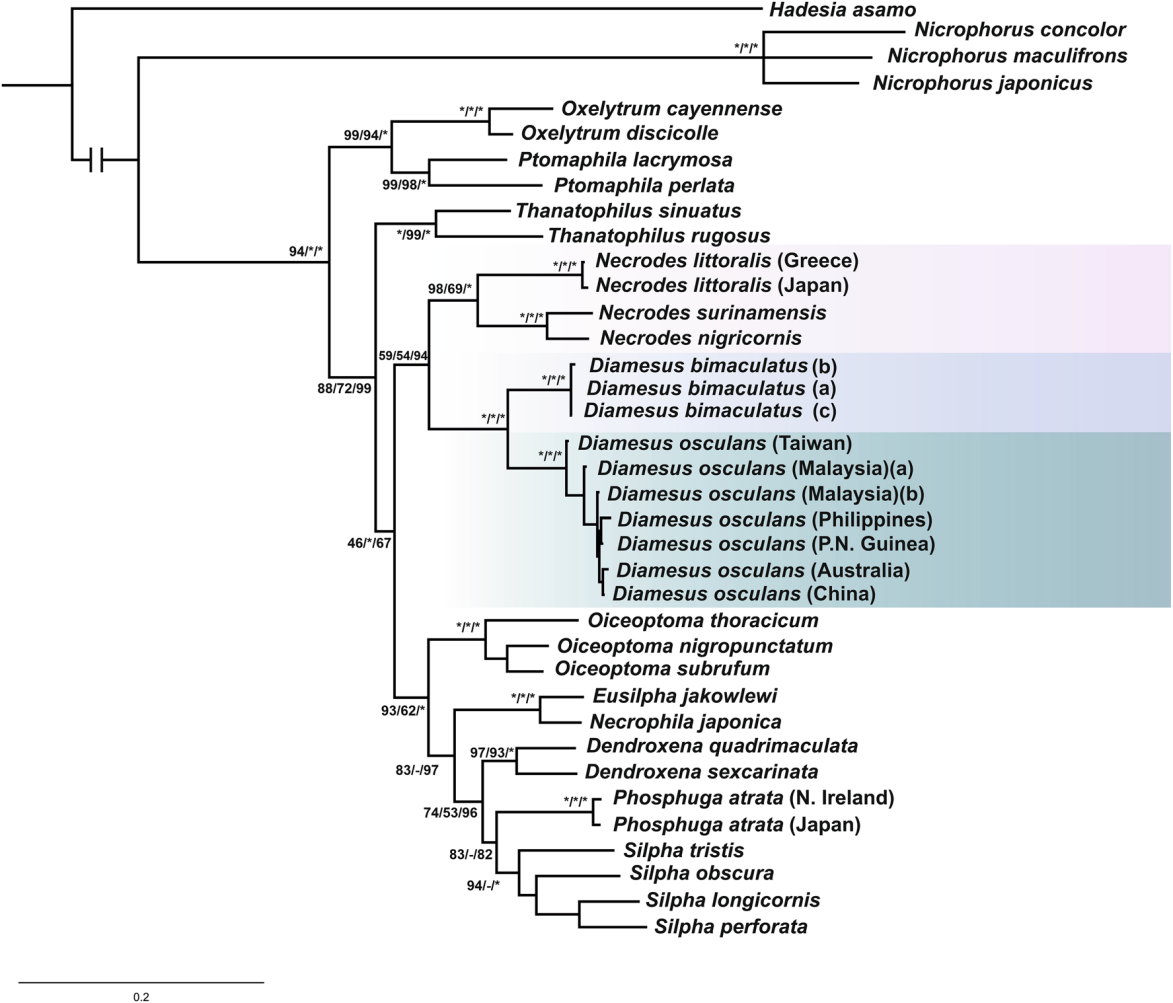
Phylogenetic tree of Silphinae based on the topology calculated by Bayesian interference. Numbers next to branches show the posterior probability and bootstrap values of maximum likelihood (ML)/maximal parsimony (MP)/Bayesian interference (BI). *Hadesia asamo* Perreau & Pavićević, 2008 (Leiodidae) was selected as outgroup (* = 100, − < 50).

**Figure 2:**
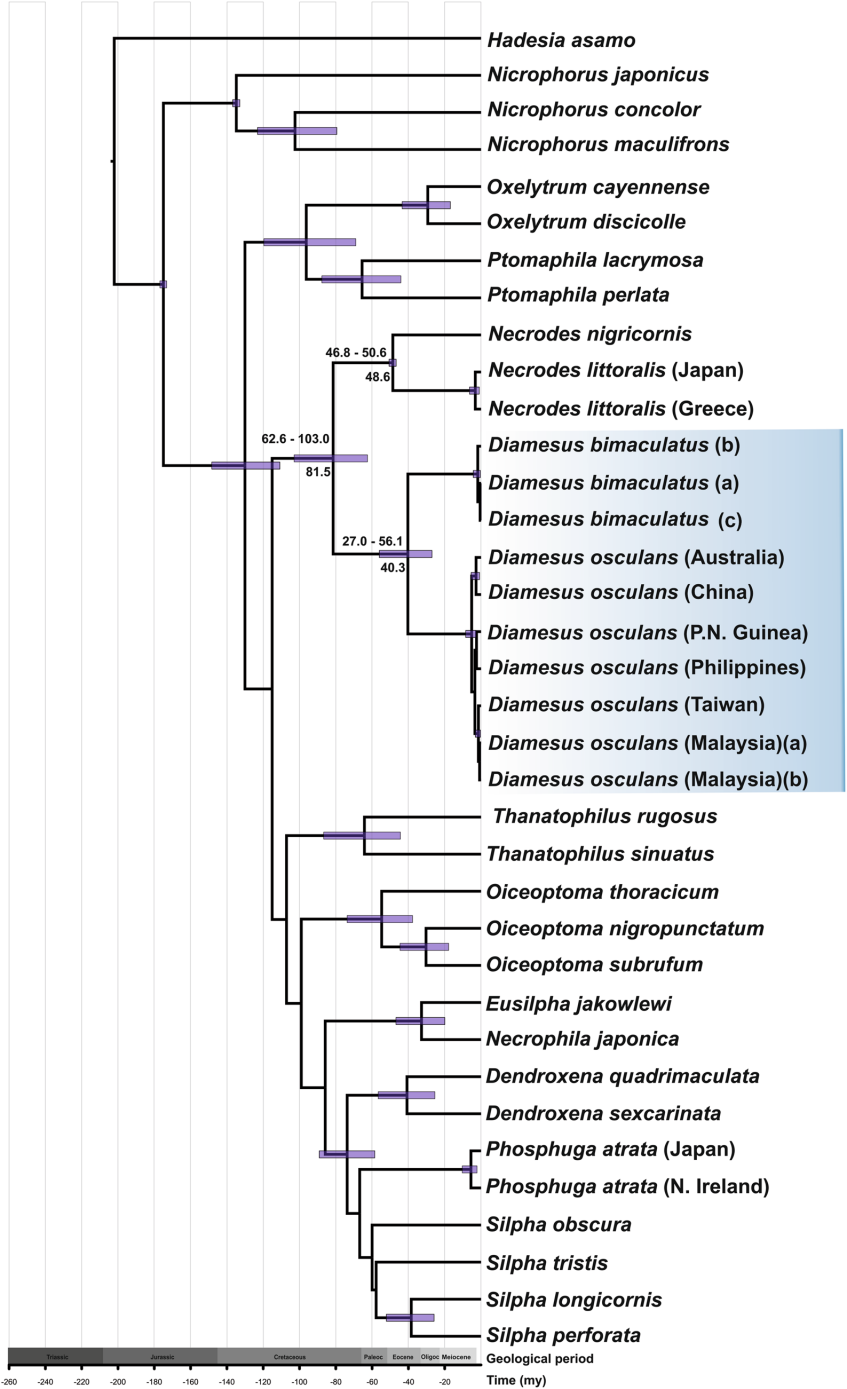
Estimation of the divergence time based on mitochondrial genes COI and 16S. Posterior probability node bars show the 95% hight posterior density intervals around the mean age (above bars) and the median age is indicated under branches. The scale axis indicates time in mya.

The aim of this paper is to employ an integrative taxonomy approach for delimitation of the species of *Diamesus*. Classical morphology is applied to redescribe adults of both species of *Diamesus*, focusing on details of external morphology (including characters of male and female genitalia), to demonstrate the validity of both taxa, which were mostly differentiated based on elytral colour pattern. We infer the phylogenetic placement of *Diamesus* within the subfamily Silphinae and compare differences between both species of *Diamesus*, based on three concatenated genes—two mitochondrial (COI, protein coding; 16S rDNA, ribosomal) and one nuclear (28S, ribosomal DNA). We also summarize and provide maps of the detailed distributions of both species as well as species distribution models based on the occurrence data (Figs. 1, 2).

## Results

*Diamesus* Hope, 1840 and *Necrodes* Leach, 1815 are two genera similar in appearance^1^. Adults of both can be characterized by a combination of the following characters: (1) body distinctly flattened dorsally, subtrapezoidal in shape in dorsal view, expanding posteriorly; (2) eyes large, prominent laterally; (3) pronotal postcoxal lobe narrow and prolonged posteriorly in lateral view; and (4) trochanter in large males flattened, extended or with the mesofemora in large males distinctly expanded, with small spine or spines along posterior apical portion^10^. The two genera can be separated using the following identification key:

1. Pronotum subconical, widest before posterior angles. Antenna black, with ultimate antennomere orange (Fig. 5b). Body large, body length 22–49 mm (Eastern Palaearctic, Oriental to Australian). … *Diamesus* Hope, 1840
2. Pronotum orbicular, widest toward middle. Antenna completely black, or with the last three antennomeres orange (Fig. 5a). Body generally smaller, body length 14–30 mm (Holarctic). … *Necrodes* Leach, 1815

**Figure 3:**
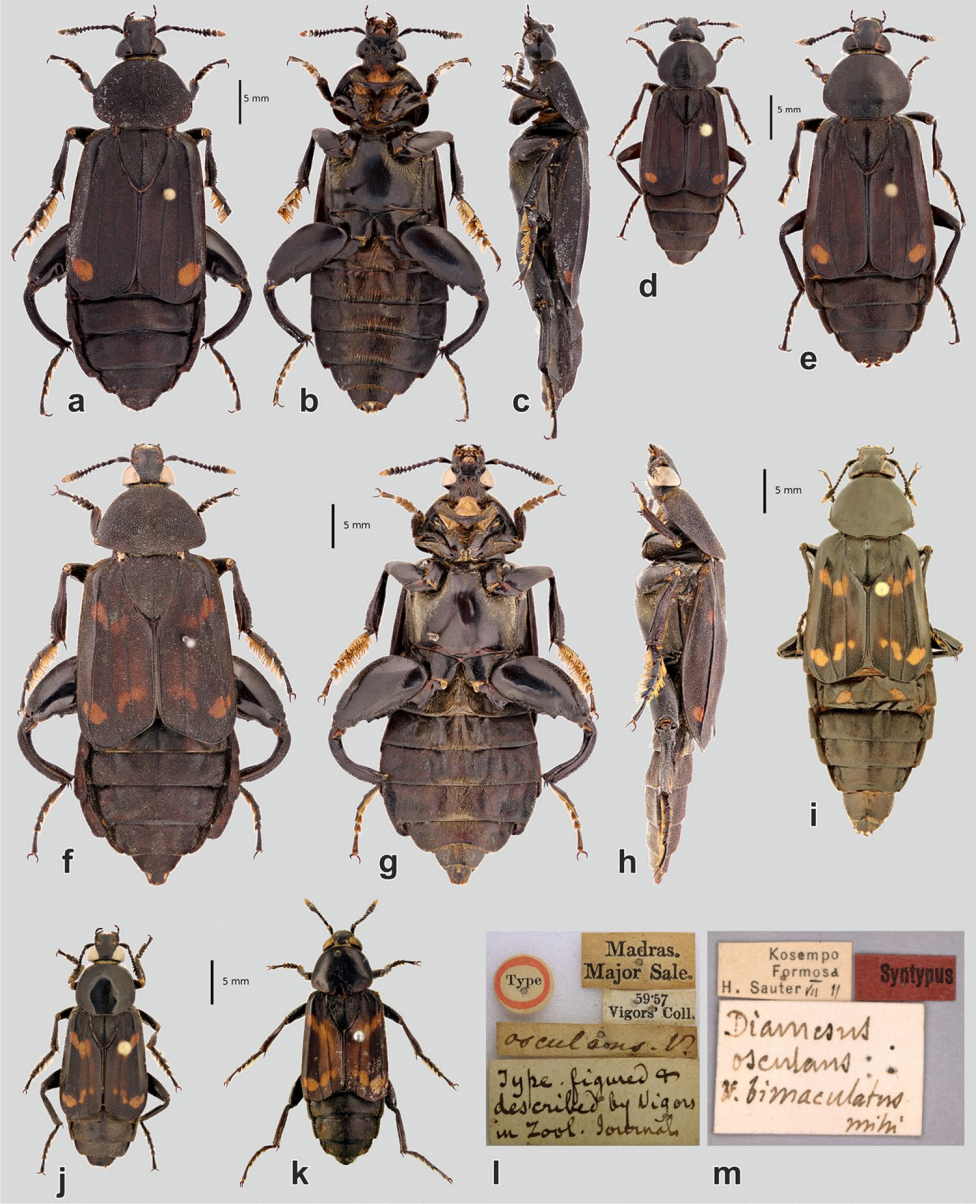
*Diamesus bimaculatus* Portevin, 1914, habitus of adults: (**a–c**) lectotype of *Diamesus osculans* var. *bimaculatus*, large male, dorsal, ventral, and lateral view; (**d**) paralectotype, small male, dorsal view; (**e**) paralectotype, female, dorsal view. *D. osculans* (Vigors, 1825), habitus of adults: (**f–h**) large male (Laos: Phu Pane Mt.), dorsal, ventral, and lateral view; (**i**) female (Laos: Nam Ha), dorsal view; (**j**) small male (Malaysia: Batu 25–26), dorsal view; (**k**) holotype of *Necrodes osculans*, small male, dorsal view. Labels: (**l**) holotype of *Necrodes osculans*; (**m**) lectotype of *D. osculans* var. *bimaculatus*.

**Figure 4:**
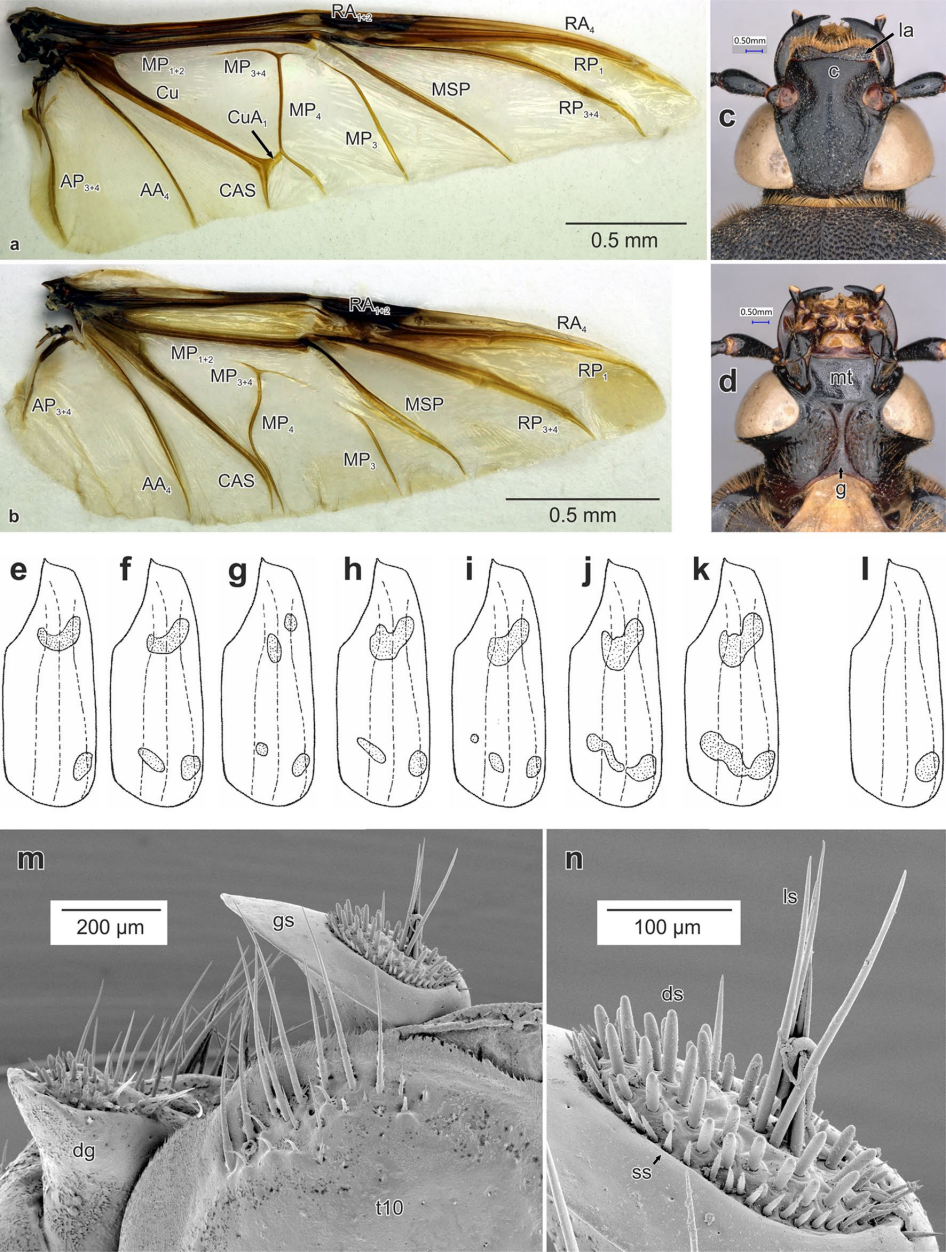
Morphological details of *Diamesus* Hope, 1840 and *Necrodes* Leach, 1815. Hind wings: (**a**) *D. osculans* (Vigors, 1825) (Vietnam: Alona forest), female; (**b**) *N. littoralis* (Linnaeus, 1758) (Czech Republic: Ladná env.), female. *Diamesus osculans*, head: (**c**) dorsal view; (**d**) ventral view. Variation in elytral maculation, dorsal view, schematized: (**e–k**) *D. osculans*; (**l**) *D. bimaculatus* Portevin, 1914. Female genitalia in dorsal view, *D. osculans* (Laos: 51 km N Sekong), SEM: (**m**) tergum 10, distal gonocoxite and gonostylus, arrangement of setae; (**n**) detail of gonostylus. Abbreviations: *c* clypeus, *dg* distal gonocoxite, *ds* digitiform setae, *g* gula, *gs* gonostylus, *la* labrum, *ls* long setae, *mt* mentum, *ss* small setae, *t10* tergum 10.

**Figure 5:**
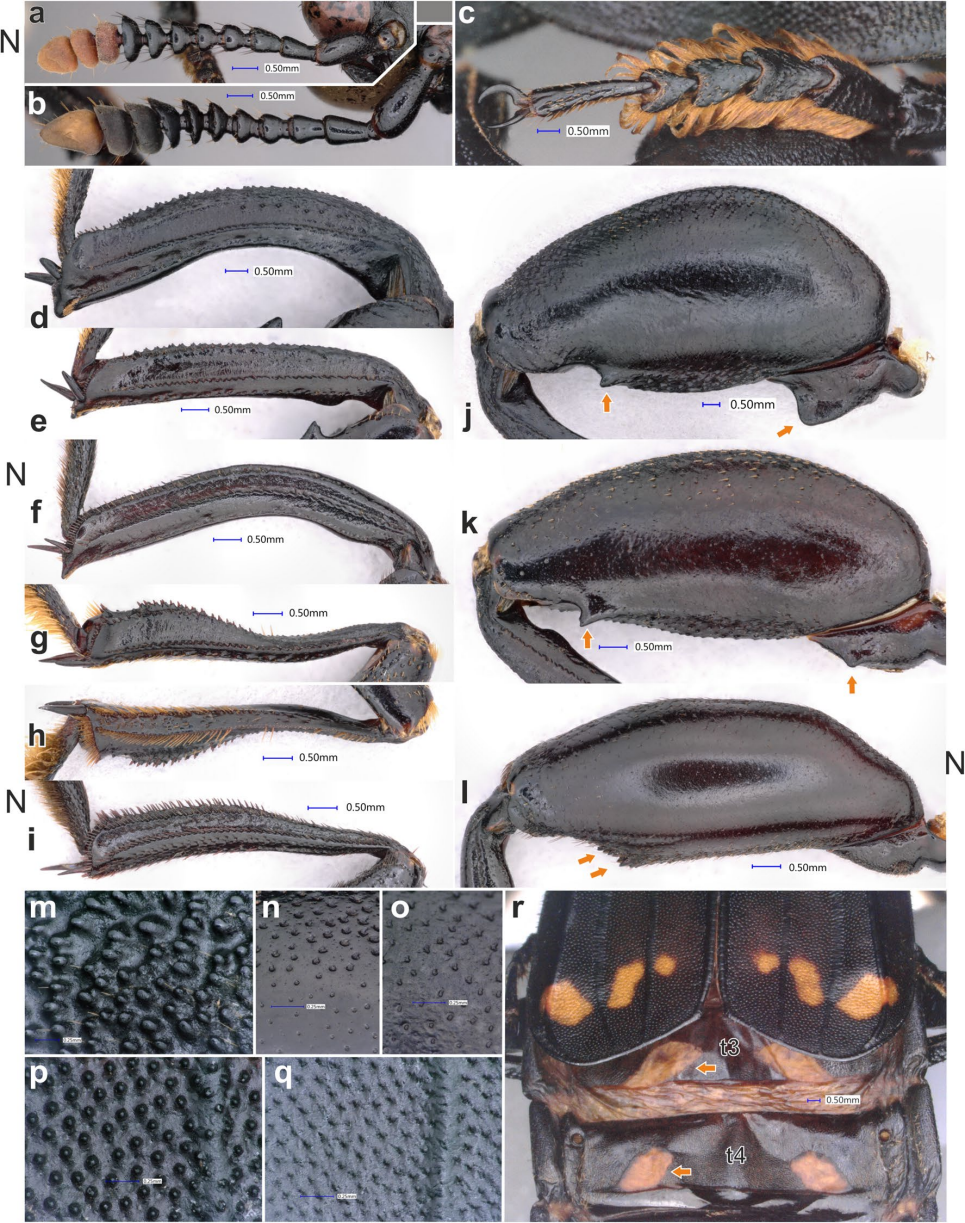
Morphological details of *Diamesus* Hope, 1840 and *Necrodes* Leach, 1815. Right antenna in dorsal view: (**a**) *N. littoralis* (Linnaeus, 1758) (Czech Republic: Praha-Háje); (**b**) *D. osculans* (Vigors, 1825) (Laos: Phu Pane Mt.). Male mesotarsus in dorsolateral view: (**c**) *D. osculans* (Laos: Phu Pane Mt.). Male metatibia in outer lateral view: (**d**) *D. osculans* (Indonesia: Tanah Labang env.), large male; (**e**) *D. osculans* (Indonesia: Mt. Talagaranu), medium male; (**f**) *N. littoralis* (Czech Republic: Praha-Háje), large male. Male mesotibia: (**g**) *D. osculans* (Indonesia: Mt. Talagaranu), outer lateral view, medium male; (**h**) the same, inner lateral view; (**i**) *N. littoralis* (Czech Republic: Praha-Háje), outer lateral view, large male. Male metafemur in outer lateral view: (**j**) *D. osculans* (Indonesia: Tanah Labang env.), large male; (**k**) *D. osculans* (Indonesia: Mt. Talagaranu), medium male; (**l**) *N. littoralis* (Czech Republic: Praha-Háje), large male. Pronotum of *D. osculans* in dorsal view, detail of surface: (**m**) large male (Laos: Phu Pane Mt.); (**n**) small male (Malaysia: Batu 25–26); (**o**) large female (Laos: Nam Ha). Elytra of *D. osculans* in dorsal view, detail of surface: (**p**) large male (Malaysia: Batu 25–26); (**q**) large female (Laos: Nam Ha). Detail of elytral apex and abdominal tergites 3 and 4 of *D. osculans* in postero-dorsal view: (**r**) large female (Laos: Nam Ha). Abbreviations: *t3* tergite 3, *t4* tergite 4.

**Figure 6:**
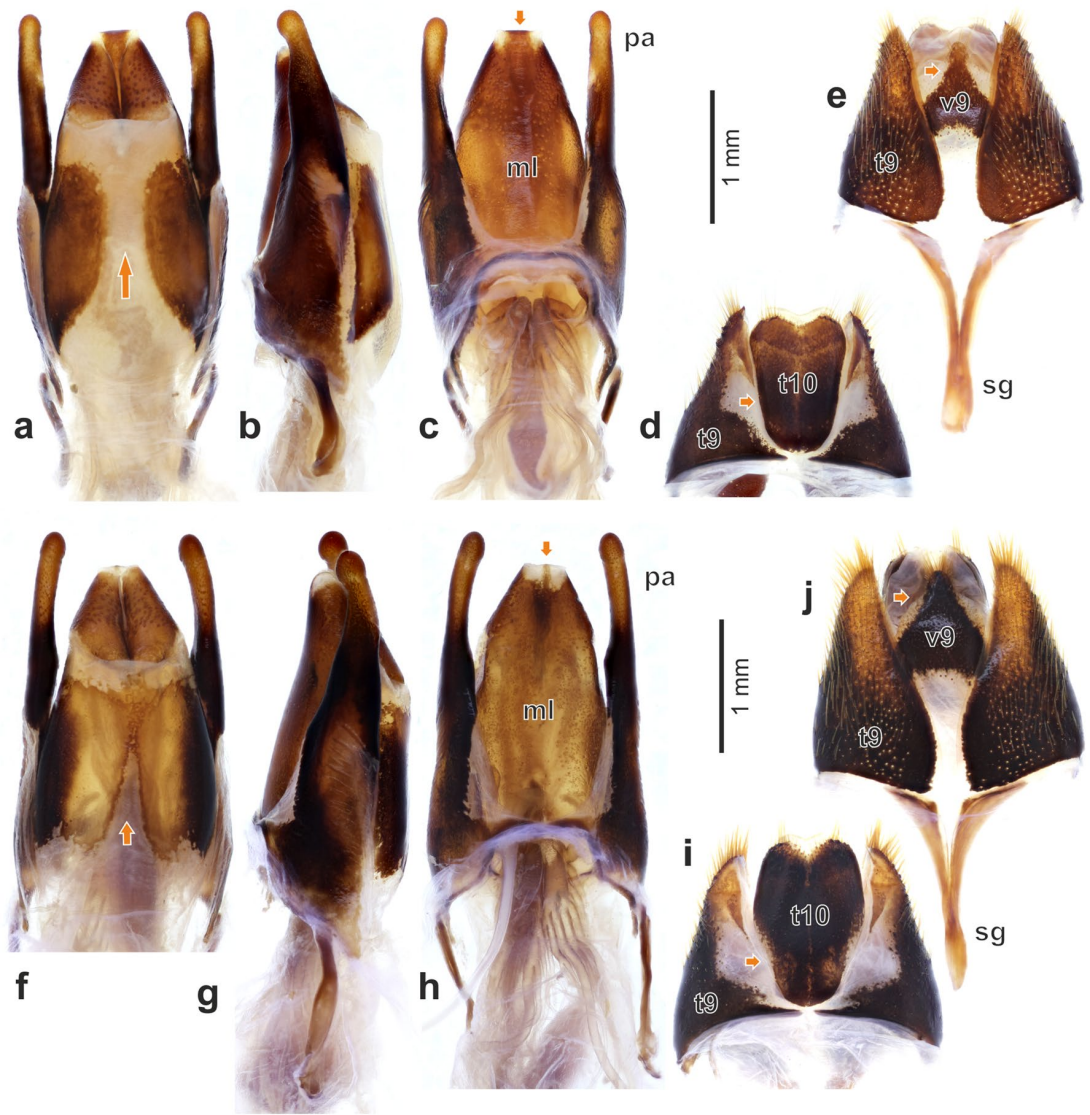
Male genitalia of *Diamesus* Hope, 1840: (**a–c**) *D. bimaculatus* Portevin, 1914 (Taiwan: Baling), aedeagus in dorsal, lateral and ventral view; (**d–e**) *D. bimaculatus* (Taiwan: Baling), male segments 9–10 in dorsal and ventral view; (**f–h**) *D. osculans* (Vigors, 1825) (Indonesia: Mt. Talagaranu), aedeagus in dorsal, lateral and ventral view; (**i–j**) *D. osculans* (Indonesia: Mt. Talagaranu), male segments 9–10 in dorsal and ventral view. Abbreviations: *ml* median lobe, *pa* paramere, *sg* spiculum gastrale, *t9* tergite 9, *t10* tergite 10, *v9* ventrite 9.

**Figure 7:**
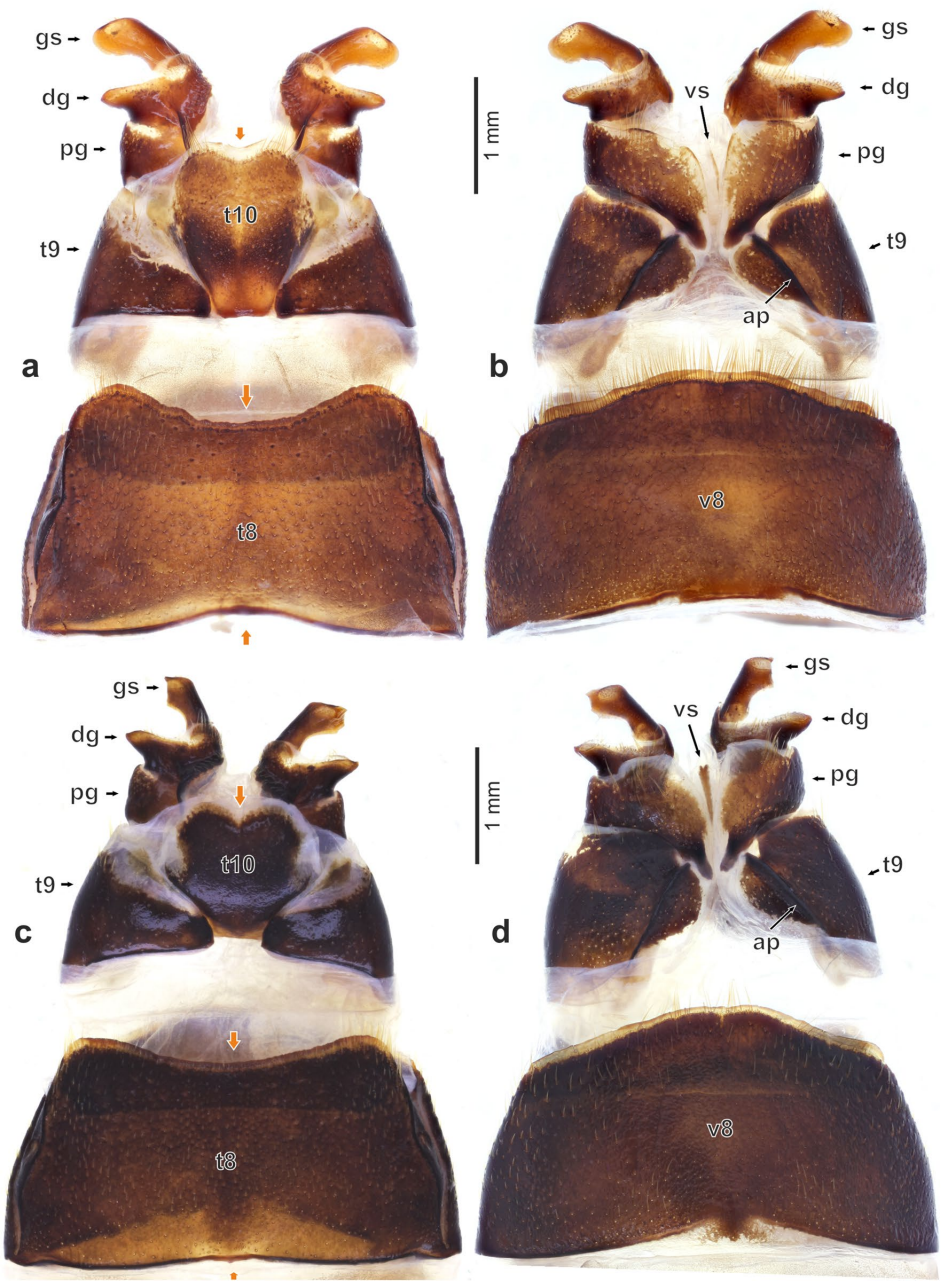
Female genitalia of *Diamesus* Hope, 1840: (**a–b**) *D. bimaculatus* Portevin, 1914 (Taiwan: Baling), dorsal and ventral view; (**c–d**) *D. osculans* (Vigors, 1825) (Indonesia: Mt. Talagaranu), dorsal and ventral view. Abbreviations: *ap* apodeme, *dg* distal gonocoxite, *gs* gonostylus, *pg* proximal gonocoxite, *t8* tergite 8, *t9* tergite 9, *t10* tergite 10, *v8* ventrite 8, *vs* ventral sclerite.

***Diamesus*** **Hope, 1840**

(Figs. 3a–m, 4a,c–n, 5b,c–e,g–h,j–k,m–r, 6a–j, 7a–d)

*Diamesus* Hope, 1840: 149 (description by indication, type species *Necrodes osculans* Vigors, 1825, by original designation).

*Diamesus* Hope: Kraatz 1876: 355 (redescription).

*Diamesus* Kraatz: Portevin 1926: 167 (redescription).

### Diagnostic description

Body large, robust, dorsolaterally flattened. Body length very variable, between 22 and 49 mm (48 specimens measured), generally considered to be the largest species of Silphinae^15^.

#### Colouration

Body brown to dark brown/black (pale brown in teneral specimens), with orange ultimate antennomere and orange spots or bands of various extent on elytra (Fig. 3a–k), two pairs of orange spots on tergum 3 and 4 (Fig. 5r) are usually covered by elytra, exposed probably only during flight.

#### Head

Clypeus with regularly rounded anterior margin (Fig. 4c, *c*). Antenna inserted in front of eyes, with elongate depression going to mid-length of eyes (Fig. 4c). Labrum anteriorly with wide medial emargination (Fig. 4c, *la*). Eyes large, prominent (Fig. 4c,d). Ventrally, head with large, flat, heavily sclerotized mentum, subtrapezoid in shape (Fig. 4d, *mt*), and narrow, hour-glass shaped gula (Fig. 4d, *g*).

#### Antenna

With 11 antennomeres, scapus large, long as three subsequent antennomeres (Fig. 5b). Antennal club loosely formed, antennomeres 6–8 transverse, 9–11 more robust; antennomeres 1–8 glossy, 9–11 with opaque surface; antenna black, antennomeres 9–10 sometimes greyish, only ultimate antennomere contrastingly coloured, orange (Fig. 5b).

#### Pronotum

Subconical, narrow anteriad and regularly extending posteriad, suddenly narrowing posteriorly (Fig. 3f); surface weakly, regularly vaulted; posterolaterally sometimes with a shallow, transverse pair of depressions. Anterior half sometimes in males with loosely indicated carina along medial line (Fig. 3a,d). Surface with distinct granulation in large males (Fig. 5m), or with coarse punctation in small males and females, very fine and superficial medially (Fig. 5n–o). Anterior margin bordered by dense, long, orange setation (Fig. 4c).

#### Scutellum

Very large (ca. 0.5 as wide as pronotum), triangular, with longitudinal, elevated keel along medial line, better indicated in males (Fig. 3i).

#### Elytra

Apically truncate, usually exposing abdominal tergites 5–8 in rest position (Fig. 3a,d–f,i–k). Outer costa raised, two inner costae flat (Figs. 3f, 5r). Surface finely granulate in large males (Fig. 5p) or coarsely punctate in small males and in females (Fig. 5p,q).

#### Hind wings

Well developed, ca. 1.7 times as long as elytra. Veins MP_3+4_ and MP_3_ more elongate than in *Necrodes* (Fig. 4a,b). Anal field broader, vein AP_3+4_ more elongated than in *Necrodes* (Fig. 4a,b).

#### Legs

Generally large and robust (Fig. 3a–k). Protibia straight, with two combs formed by large setae along inner and outer lateral margin. Protarsi with expanded tarsomeres 1–4 in males (Fig. 3a–d,f–h,j,k), unmodified in females (Fig. 3e,i). Mesotibia dorsoventrally flattened in basal part, suddenly expanded apically (Fig. 5g,h). External lateral surface of mesotibia with a longitudinal carina of small denticles (Fig. 5g), internal lateral surface with a comb of long setae along its length, and another transverse comb apically (Fig. 5h). Dorsal surface in apical part granulated, granules combined with robust, short setae (Fig. 5g,h). Large males with mesotarsomeres 1–4 expanded, flattened, with long, orange lateral and ventral setation (Figs. 3b,g, 5c), almost unmodified in smaller males and in females (Fig. 3d,e,i–k). Metatrochanter of large males flattened, apically broadened (Fig. 5j), in smaller males only with a small lateral, subapical denticle (Fig. 5k), in females unmodified. Metafemora greatly expanded in large males (Figs. 3a–b,f–g, 5j), less expanded in medium-sized males (Fig. 5k), unmodified in small males and females (Fig. 3d–e,i–k). Inner margin of metafemora with a single, subapical denticle in large and medium-sized males (Fig. 5j–k), unmodified, simple in small males and in all females. Metatibia expanded apically and distinctly bent in large males (Fig. 5d); only slightly expanded, straight, with proximal inner denticle in medium-sized males (Fig. 5e); unmodified, straight, in small males and in all females (Fig. 3d–e,i–k).

#### Abdomen

Dorsally, with distinctly developed lateral lobes, especially in large males (Fig. 3a,f). Tergum 3 and 4 each with a pair of orange spots (Fig. 5r). Ventrally, medially with a path of long, erected, orange to brown setae (Fig. 3b,g). Surface finely granulate in large males, or coarsely punctate in small males and in females.

#### Male genitalia

Tergum 9 separated into two separate, lateral sclerites, which are largely desclerotized on inner portion in dorsal view (Fig. 6d,i, *t9*). Basal part ventrally prolonged into long, anteriorly facing, V-shaped *spiculum gastrale* (Fig. 6e,j, *sg*). Ventrite 9 small, triangular, lateral parts at base covered by tergite 9 in ventral view (Fig. 6e,j, *v9*). Tergite 10 large, oval, narrowing basally and widely emarginate medially on posterior margin (Fig. 6d,i, *t10*). Aedeagus with short, broad, dorsoventrally slightly flattened median lobe (Fig. 6a–c,f–h, *ml*). Median lobe unsclerotized medially (in different extent, completely or only basally) in dorsal view (Fig. 6a,f). Apex of median lobe narrowly or more broadly desclerotized laterally in ventral view (Fig. 6c,h). Paramere robust, slightly longer than median lobe, apex slightly expanded (Fig. 6a–c,f–h, *pa*).

#### Female genitalia

Tergite 8 not or only weakly emarginate anteriorly; and very widely, distinctly emarginate medially on posterior margin (Fig. 7a,b, *t8*). Tergum 9 separate into two lateral sclerites (Fig. 7a–d, *t9*). Ventrally, tergite 9 with more sclerotized, almost longitudinal apodeme (Fig. 7b,d, *ap*). Tergite 10 oval, heart-shaped, laterally weakly sclerotized or partly unsclerotized, emarginate posteriorly in dorsal view (Fig. 7a,b, *t10*). Paired proximal gonocoxites developed mostly ventrally (Fig. 7a–d, *pg*), medially separated by narrow, longitudinal ventral sclerite (Fig. 7b,d, *vs*). Distal gonocoxites expanded into lateral lobes, with its posterior part flattened, desclerotized, bearing long setae on ventral margin and also centrally, and numerous short, digitiform setae (Fig. 4m, *dg*). Gonostylus with apical part expanded into elliptic, asymmetrical plate with extended external part (Figs. 4m, *gs*, 7a–d, *gs*). Its posterior part bears oval, desclerotized sensory field. Arrangement of setae consists of an outer circle of small, pointed setae (Fig. 4n, *ss*); numerous digitiform setae covering the sensory field (Fig. 4n, *ds*), and several long setae, located in the middle (Fig. 4n, *ls*).

### Variability and sexual dimorphism

Extremely variable species in body size and proportions. Large males with granulate surface (Fig. 5m,p), small males and all females with coarse punctures on surface (Fig. 5n,o,q). Large males also with more developed lateral lobes on abdomen (Fig. 3a,f). Large males with expanded, flattened mesotarsomeres bearing long, yellow setation (Fig. 5c) and with extremely expanded metafemora (Figs. 3a–b,f–g, 5j); medium-sized males with less expanded metafemora (Fig. 5k); small males and all females with unmodified mesotarsomeres and simple metafemora. Also, metatibia is expanded and bent in large males (Fig. 5d), slightly expanded but straight in medium-sized males (Fig. 5e) and unmodified in small males and in all females (Fig. 3d,e,i–k).

### Morphometry

We found significant differences between pronotum width to length ratio for males and females of *D. bimaculatus* (t value = 2.555, p value = 0.0144). Partial significance was found in elytra width to length ratio at species level (t value = 1.798, p value = 0.0796), but not between males and females (t value = 0.18, p value = 0.8578). For body length and scutellum length to width ratio we did not find any statistically significant difference at species level (t value = 0.74, p value = 0.463; t value = − 0.31, p value = 0.758 respectively) nor sex (t value = 0.603, p value = 0.55; t value = − 0.198, p value = 0.844 respectively). Therefore, our results do not support the observation of Portevin^10^ that the two species can be differentiated based on scutellum length to width ratio. For a graphical summary of the morphometric analysis see SM4 (a–d).

### Taxonomy

Hope^6^ published the name *Diamesus* in a table, and labelled “Nec.[rodes] osculans, Vigors”, the only included species, as “typical species”. Although this is the only included species name in *Diamesus*, according to Article 68.2 of the Code, *Necrodes osculans* was designated as the type species of *Diamesus* by original designation, not by monotypy^26^. As Hope^6^ provided no formal description of *Diamesus*, Kraatz^27^ provided its formal description. This also led Portevin^10^ to the conclusion that *Diamesus* Hope, 1840 is a *nomen nudum*, and he attributed this generic name to Kraatz. However, for a new genus-group name published before 1931, its publication in combination with available specific name is treated as an ‘indication’ and makes *Diamesus* Hope, 1840 available, according to Article 12.2.5 of the Code^26^.

### Phylogenetic analysis

In total, 37 specimens were used for the phylogenetic analysis, including outgroup (Table 1). Concatenated sequences resulted into an alignment of a total length of 1702 bp (COI 727 bp, 16S 451 bp, and 28S 524 bp). The monophyly of the two tribes within the subfamily Silphinae, Silphini (ML: Bootstrap value 94/MP: Bootstrap value 100/BI: Posterior probability 100) and Nicrophorini was supported (100/100/100) (Fig. 1). The two genera forming the sister clade to remaining members of the of the tribe Silphini—*Ptomaphila* Kirby & Spence, 1828 and *Oxelytrum* Gistel, 1848—were strongly supported (99/94/100) as well as the well-established genera *Thanatophilus* Leach, 1815 and *Oiceoptoma* Leach, 1815 (Fig. 1). The focal genus of this study—*Diamesus*—was represented by 3 individuals of *D. bimaculatus* (all from Taiwan) and 7 individuals of *D. osculans* (sampled throughout Asia and Australia, incl. Taiwan). The clade consisting of these two species was strongly supported (100/100/100) and the two species were reciprocally monophyletic with strong support (both 100/100/100). The support of the lineage containing both sister genera, *Necrodes* and *Diamesus*, was 59/54/94 (Fig. 1).

**Table 1:** Specimens used in the molecular analysis.

Species	Locality	COI mtDNA	16S rDNA	28S rDNA
*Dendroxena quadrimaculata*	Bulgaria	**OM802114**	**OM829821**	**OM829834**
*Dendroxena sexcarinata*	Japan	AB606653	AB285535	AB285567
*Diamesus bimaculatus (a)*	Taiwan	**OM802119**	**OM829826**	**OM829837**
*Diamesus bimaculatus (b)*	Taiwan	**OM802120**	**OM829827**	**OM829838**
*Diamesus bimaculatus (c)*	Taiwan	**OM802116**	**OM829823**	x
*Diamesus osculans*	Philippines	**OM802115**	**OM829822**	**OM829835**
*Diamesus osculans*	Papua New Guinea	**OM802117**	**OM829824**	**OM829836**
*Diamesus osculans*	Malaysia (b)	**OM802118**	**OM829825**	x
*Diamesus osculans*	Australia	JQ582730	JQ913546	JQ913534
*Diamesus osculans*	China	NC_045874	NC_045874	x
*Diamesus osculans*	Malaysia (a)	AB606432	AB285554	AB285586
*Diamesus osculans*	Taiwan	**OP985156**	**OP985127**	x
*Necrophila jakowlewi*	South Korea	AB761600	AB285547	AB285579
*Necrodes littoralis*	Greece	**OM802121**	**OM829828**	**OM829840**
*Necrodes littoralis*	Japan	AB606438	AB285536	AB285568
*Necrodes nigricornis*	Japan	AB606433	AB285544	AB285576
*Necrodes surinamensis*		KC977956	x	KJ845002
*Necrophila japonica*	Japan	AB606552	AB285539	AB285571
*Nicrophorus concolor*	Japan	EU147421	AB285555	AB285587
*Nicrophorus japonicus*	China	JN086494	AB285560	AB285592
*Nicrophorus maculifrons*		GQ343203	AB285556	AB285588
*Oiceoptoma nigropunctatum*	Japan	AB606470	AB285540	AB285572
*Oiceoptoma subrufum*	Japan	AB606486	AB285537	AB285569
*Oiceoptoma thoracicum*	Japan	AB606436	AB285549	AB285581
*Oxelytrum cayennense*		DQ222022	DQ202619	x
*Oxelytrum discicolle*	Ecuador	AB606431	AB285552	AB285584
*Phosphuga atrata*	Northern Ireland	**MW624525**	**MW642418**	**OM829841**
*Phosphuga atrata*	Japan	AB376111	AB285541	AB285573
*Ptomaphila lacrymosa*	Australia	KC977954	JQ913548	JQ913539
*Ptomaphila perlata*	Australia	JQ582719	JQ913551	JQ913540
*Silpha longicornis*	Japan	AB376125	AB285538	AB285570
*Silpha obscura*	Greece	**OM802122**	**OM829829**	**OM829842**
*Silpha perforata*	Japan	AB438997	AB285534	AB285566
*Silpha tristis*	Canada	AB376109	AB285542	AB285574
*Thanatophilus rugosus*	Japan	AB606434	AB285546	AB285578
*Thanatophilus sinuatus*	Japan	AB606435	AB285548	AB285580
*Hadesia asamo*	Bosnia and Herzegovina	KX223650	KX223615	KX223628

In bold, accession numbers of newly obtained sequences deposited in NCBI (https://www.ncbi.nlm.nih.gov/) and x represents missing sequence.

### Estimation of the divergence time

An alignment of 36 specimens was used for the estimation of the divergence time. Using three nodal priors resulted in an estimated median age of 81.5 mya for the clade consisting of the genera *Necrodes* and *Diamesus* (95% mean posterior interval age estimation of 62.6–103.0 mya) and estimated median age of 40.3 mya for the two species of *Diamesus—Diamesus osculans* and *Diamesus bimaculatus* (95% mean posterior interval age estimation of 27.0–56.1 mya).

### Species distribution models

**Figure 8:**
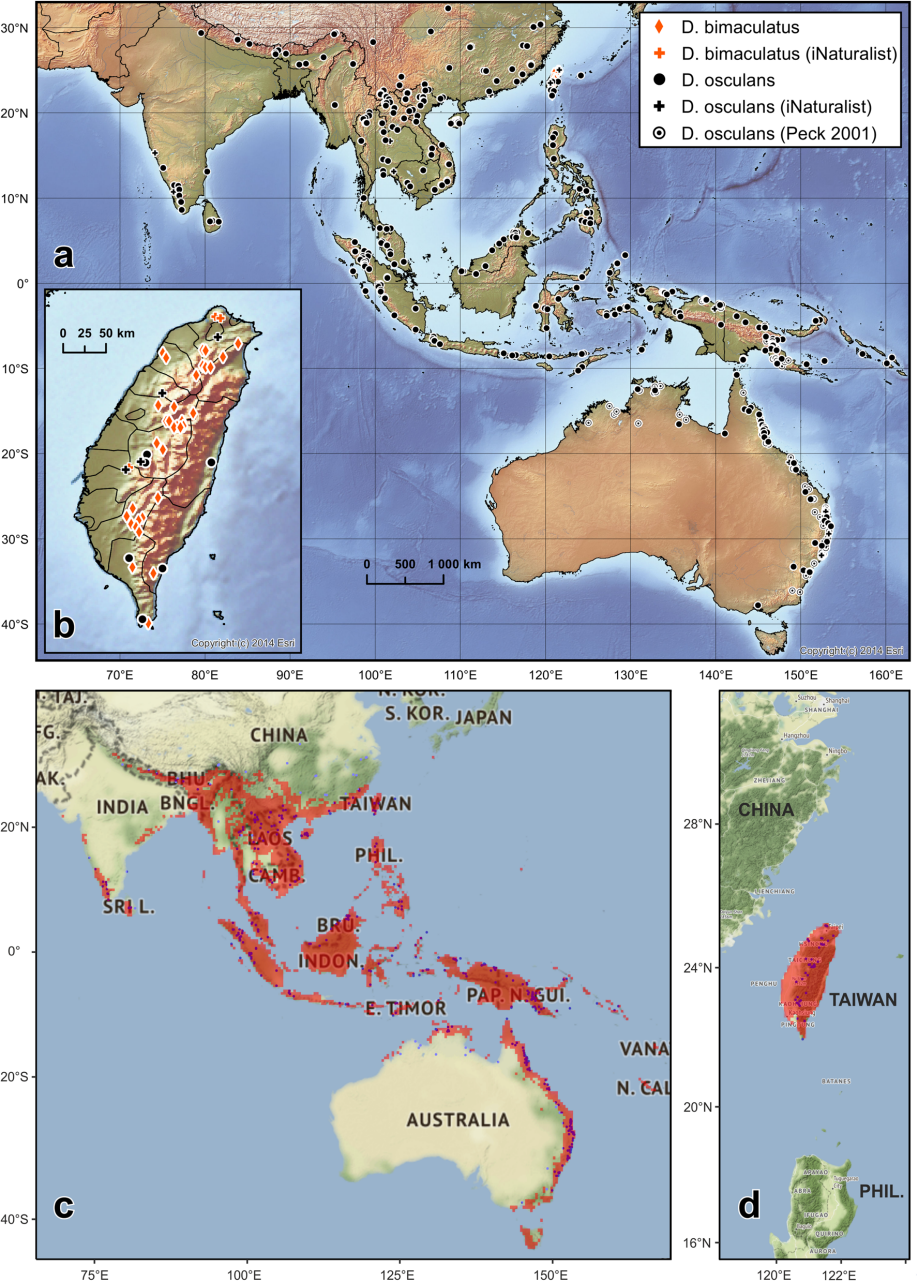
Distribution of *Diamesus bimaculatus* Portevin, 1914 and *D. osculans* (Vigors, 1825). (**a**) general distribution; (**b**) detailed distribution of both species in Taiwan, produced by ESRI ArcMap 10.8.1 of ArcGIS Desktop 10.8.1 suite (https://www.esri.com/en-us/arcgis). Species distribution models of *D. osculans* (**c**) and *D. bimaculatus* (**d**) were created using MaxEnt v.3.4.4, https://biodiversityinformatics.amnh.org/open_source/maxent/.

We used 40 observations of *D. bimaculatus* and 513 observations of *D. osculans* to model their distribution. Both models of *D. bimaculatus* and *D. osculans* showed high scores of area under curve (AUC) values (0.9887 and 0.8797 respectively). The maps were reclassified to binomial values (predicted presence/absence) using maximum training sensitivity plus specificity Cloglog thresholds, which were 0.2357 and 0.4005 respectively (see Fig. 8a,b). The maps of predicted presence behaved well in the case of *D. bimaculatus* and presence was not predicted outside of Taiwan (Fig. 8d). However, in the case of *D. osculans* (Fig. 8c), the model seemed to behave conservatively for some parts of China and Australia, where some localities were not considered probable by the model. Although, in other regions (e.g., Japan, New Caledonia) the model predicted the presence of this species was possible despite the separation of these regions from the rest of the distribution by large water masses and lack of specimen evidence.

### Key to adults of *Diamesus* Hope, 1840

1. Body dark brown to black (pale brown in teneral specimens), each elytron only with a single small orange spot near apex (Figs. 3a–e, 4l). *Male*: tergite 10 subtrapezoidal, weakly and widely emarginate on posterior margin (Fig. 6d, *t10*). Median lobe of aedeagus shorter, sclerotized parts in dorsal view 1.4 times as long as wide; in dorsal view broadly unsclerotized medially (Fig. 6a). *Female*: tergite 10 more elongate, 1.3 times as long as wide posteriorly (Fig. 7a, *t10*). (Endemic to Taiwan, Fig. 8a,b). … *D. bimaculatus* Portevin, 1914
2. Body brown (pale brown in teneral specimens), each elytron with two orange bands, variable in size and shape, bands sometimes split into 2–3 isolated spots (Figs. 3f–k, 4e–k). *Male*: tergite 10 oval, distinctly narrower in anterior part, deeply and narrowly emarginate on posterior margin (Fig. 6i, *t10*). Median lobe of aedeagus longer, sclerotized parts in dorsal view 1.6 times as long as wide; in dorsal view narrowly unsclerotized medially only in anterior part, sclerotization medially fused in central part (Fig. 6f). *Female*: tergite 10 less elongate, only 1.1 times as long as wide posteriorly (Fig. 7c, *t10*). (Widely distributed from India to Australia, incl. Taiwan, Fig. 8a,b). … *D. osculans* (Vigors, 1825)

***Diamesus bimaculatus*** **Portevin, 1914**

(Figs. 3a–e,m, 4l, 6a–e, 7a–b, 8a–b)

*Diamesus osculans* var. *bimaculatus* Portevin, 1914: 6 (description, type locality: Kosempo).

*Diamesus bimaculatus*: Portevin 1922: 3 (elevated to species).

### Type material examined

LT ♂ (SDEI) (here designated), labelled (Fig. 3m) “Kosempo [= Chiahsien or Jiaxian, Kaohsiung hsien county, ca. 23°05′N 120°35′E]/Formosa [= Taiwan]/H. Sauter [leg.] [p] VII [19]11 [hw]//Syntypus [p, red label]//Diamesus/osculans/v. bimaculatus/mihi [hw, Portevin’s MS]//LECTOTYPE ♂/Diamesus/osculans var. bimaculatus/Portevin, 1914/Jan Růžička design. 2021 [p, red label]//Diamesus/bimaculatus/Portevin, 1914/Jan Růžička det. 2021 [p]”; PLT 12 ♂♂, 5 ♀♀ (SDEI), “Kosempo/Formosa/H. Sauter [leg.] [p] VII [19]11 [hw]//Syntypus [p, red label]//Portevin det. [hw]//PARALECTOTYPE ♂ [or ♀]/Diamesus/osculans var. bimaculatus/Portevin, 1914/Jan Růžička design. 2021 [p, red label]//Diamesus/bimaculatus/Portevin, 1914/Jan Růžička det. 2021 [p]”; PLT 1 ♀ (SDEI), labelled “Portevin det. [p]//Syntypus [p, red label]//PARALECTOTYPE ♀/Diamesus/osculans var. bimaculatus/Portevin, 1914/Jan Růžička design. 2021 [p, red label]//Diamesus/bimaculatus/Portevin, 1914/Jan Růžička det. 2021 [p]”; PLT 1 ♀ (SDEI), labelled “Kosempo/Formosa/H. Sauter [leg.] [p] VII [19]11 [hw]//Syntypus [p, red label]//Diamesus/osculans Vig./v. bimaculatus mihi [hw, Portevin’s MS]//Portevin det. [hw]//PARALECTOTYPE ♀/Diamesus/osculans var. bimaculatus/Portevin, 1914/Jan Růžička design. 2021 [p, red label]//Diamesus/bimaculatus/Portevin, 1914/Jan Růžička det. 2021 [p]”;

PLT 2 ♂♂ (MNHN), labelled “Kosempo/Formosa/H. Sauter [leg.] [p] VII [19]11 [hw]//TYPE [p, red label]//Muséum Paris/Coll. M. Pic [p]//bimaculatus/var. tibialis/m. [hw, Portevin’s MS, manuscript name, ony under first ♂]//PARALECTOTYPE ♂/Diamesus/osculans var. bimaculatus/Portevin, 1914/Jan Růžička design. 2021 [p, red label]//Diamesus/bimaculatus/Portevin, 1914/Jan Růžička det. 2021 [p]”;

PLT 1 ♀ (MNHN), labelled “Kosempo/Formosa/H. Sauter [leg.] [p] VII [19]11 [hw]//Muséum Paris [p]/G. Portevin 1920 [hw]//TYPE [p, red characters on white label]//PARALECTOTYPE ♀/Diamesus/osculans var. bimaculatus/Portevin, 1914/Jan Růžička design. 2021 [p, red label]//Diamesus/bimaculatus/Portevin, 1914/Jan Růžička det. 2021 [p]”;

PLT 1 ♀  (NMNH), labelled “Kosempo/Formosa/H. Sauter [leg.] [p] VII [19]11 [hw]//PARATYPUS [p, red label]//Diamesus/osculans var/bimaculatus Port [hw]//Loan from/USNMNH/2026393 [p]//PARALECTOTYPE ♀/Diamesus/osculans var. bimaculatus/Portevin, 1914/Jan Růžička design. 2021 [p, red label]//Diamesus/bimaculatus/Portevin, 1914/Jan Růžička det. 2021 [p]”;

PLT 1 ♀ (NMNH), labelled “Kosempo/Formosa/H. Sauter [leg.] [p] VII [19]11 [hw]//COTYPUS [p, red label]//Carl F Baker/Collection [p]//Diamesus os-/culans v. bima-/culatus Port. [hw]//Diamesus/osculans Vigors/v. bimaculatus/Port. [hw, red label with black frame]//Loan from/USNMNH/2026393 [p]//PARALECTOTYPE ♀/Diamesus/osculans var. bimaculatus/Portevin, 1914/Jan Růžička design. 2021 [p, red label]//Diamesus/bimaculatus/Portevin, 1914/Jan Růžička det. 2021 [p]”;

PLT 1 ♀ (NMNH), without locality label, labelled only “COTYPUS [p, red label]//Carl F Baker/Collection [p]//Loan from/USNMNH/2026393 [p]//PARALECTOTYPE ♀/Diamesus/osculans var. bimaculatus/Portevin, 1914/Jan Růžička design. 2021 [p, red label]//Diamesus/bimaculatus/Portevin, 1914/Jan Růžička det. 2021 [p]”;

PLT 1 ♀ (BMNH), labelled: “Co-/type [p, round label with yellow margin]//PARATYPUS [p, pink label]//Formosa./H. Sauter. [leg.]/Brit. Mus./1923-61. [p]//Kosempo/Formosa/H. Sauter [leg.] [p] VII. [19]11 [hw]//Diamesus/osculans/bimacula-/tus Portev. [hw, Portevin’s MS?]//PARALECTOTYPE ♀/Diamesus/osculans var. bimaculatus/Portevin, 1914/Jan Růžička design. 2021 [p, red label]//Diamesus/bimaculatus/Portevin, 1914/Jan Růžička det. 2021 [p]”.

### Description

#### Measurements

Body length: male 27–44 mm (median 40 mm), female 33–38 mm (median 37 mm). Pronotum width to length ratio: male 1.19–1.35 (median 1.27), female 1.18–1.28 (median 1.20). Scutellum length to width ratio: male 1.02–1.29 (median 1.19), female 1.09–1.24 (median 1.16). Elytra width to length ratio: male 1.15–1.27 (median 1.22), female 1.15–1.29 (median 1.20).

#### External morphology

Body dark brown to black (pale brown in teneral specimens), each elytron only with a single small orange spot near apex (Figs. 3a–e, 4l).

#### Male genitalia

Ventrite 9 triangular, extending into slender posterior apex (Fig. 6e, *v9*). Tergite 10 subtrapezoidal, weakly and widely emarginate on posterior margin (Fig. 6d, *t10*). Median lobe shorter, sclerotized parts in dorsal view 1.4 times as long as wide. Median lobe in dorsal view broadly unsclerotized medially (Fig. 6a). Apex of median lobe widely sclerotized along median line (Fig. 6c).

*Female genitalia*. Tergite 8 with weak emargination anteriorly and distinct, deep emargination posteriorly (Fig. 7a, *t8*). Tergite 10 more elongate, 1.3 times as long as wide posteriorly (Fig. 7a, *t10*).

### Variability

Body length varies between 27 and 44 mm. Elytral colouration uniform (Fig. 3a–e).

### Bionomics

Necrophagous species, also frequently collected using UV and mercury-vapour light traps (see SM1). Label data indicate that adults have been collected at elevations from 260 to 1600 m (SM1). Seasonality: available data confirm occurrence in majority of the year (except February, November, and December), with a peak in July, declining until September (Table 2).

**Table 2:** Seasonality of *Diamesus* Hope, 1840.

Species/month	I	II	III	IV	V	VI	VII	VIII	IX	X	XI	XII	Total
*D. bimaculatus*	2	0	6	15	5	3	48	19	15	2	0	0	115
*D. osculans*	14 (2)	11 (2)	85 (1)	42 (0)	5 (0)	8 (0)	18 (3)	47 (0)	37 (0)	54 (4)	20 (0)	24 (3)	365 (15)

Data for *D. bimaculatus* Portevin, 1914 represent all records from Taiwan with a full date. Data for *D. osculans* (Vigors, 1825) are data from Bishop Museum Field Station, Wau, Papua New Guinea, taken from voucher specimens deposited in BPBM. Number of specimens (unsexed), number in brackets indicates the proportion of teneral specimens (only available for *D. osculans*).

### References on distribution

**Taiwan:** 六亀 [= Liugui District, ca. 23°00′N 120°38′E] (Nishikawa 2014)^28^; Kosempo [= Chiahsien or Jiaxian, Kaohsiung hsien county, ca. 23°05'N 120°35'E] (Portevin 1914)^7^; 日月澤 [= 日月潭 (Riyuedan), ca. 23°51′N 120°54′E] (Nishikawa 2014)^28^; Hori [= Puli, ca. 23°58′N 120°58′E] (Nishikawa 2014)^28^; Wushe [霧社, ca. 24°02′N 121°07′E] (Nishikawa 2014)^28^; ムシヤ [霧社 (Wushe), ca. 24°02′N 121°07′E]) (Nishikawa 2014)^28^; Taihoku [= Taipei, ca. 25°04′N 121°31′E] (Miwa 1931)^29^.

**Distribution.** Endemic species to Taiwan (Fig. 8a–b).

### *Diamesus osculans* (Vigors, 1825)

(Figs. 3f–l, 4a,c–k,m–n, 5b–e,g,h,j,k,m–r, 6f–j, 7c,d, 8a,b).

*Necrodes osculans* Vigors, 1825: 537 (description, type locality: Indiâ Orientali).

*Necrodes bifasciatus* Dejean, 1833: 118 (unavailable, no diagnosis, locality: Java).

*Diamesus osculans*: Hope 1840: 149 (new combination).

*Diamesus osculans* var. *reductus* Pic, 1917: 2 (description, type locality: Sumatra).

*Diamesus osculans* ab. *diffusus* Portevin, 1926: 172 (unavailable, infrasubspecific name).

*Dimesus osculans* var. *reductus*: Růžička & Schneider 2004: 230 (synonymy with *D. osculans*).

### Type material examined

HT ♂ of *Necrodes osculans* (BMNH), labelled (Fig. 3l) “Type [p, round label with red border]//Madras. [ca. 13°05′N 080°16′E]/Major Sale. [p]//59.57/Vigors’ Coll. [p]//osculans. V. [hw]//Type. figured &/described by Vigors/in Zool. Journal [hw]”.

HT ♀ of *Diamesus osculans* var. *reductus* (MNHN), labelled “Palembang [Sumatera Selatan province, ca. 03°00′S 104°45′E]/Sumatra [p]//420 [p]//v. reductus/Pic [hw, Pic’s MS]//TYPE [p, red label]//type [hw, Pic’s MS]//Muséum Paris/Coll. M. Pic [p]//Diamesus/osculans/(Vigors, 1825)/Jan Růžička det. 2002”.

### Description

#### Measurements

Body length: male 22–49 mm (median 38 mm), female 28–44 mm (median 41 mm). Pronotum width to length ratio: male 1.18–1.38 (median 1.24), female 1.16–1.28 (median 1.21). Scutellum length to width ratio: male 0.89–1.20 (median 1.14), female 1.05–1.33 (median 1.13). Elytra width to length ratio: male 1.14–1.54 (median 1.29), female 1.17–1.32 (median 1.26).

#### External morphology

Body brown (pale brown in teneral specimens), each elytron with two orange bands, variable in size and shape, bands sometimes split into 2–3 isolated spots (Figs. 3f–k, 4e–k), rarely also the surface of elytron between bands is light brown to orange, probably mostly in subteneral specimens (Fig. 3k).

#### Male genitalia

Ventrite 9 triangular, extending into broad, regularly rounded posterior apex (Fig. 6j, *v9*). Tergite 10 oval, distinctly narrower in anterior part, deeply and narrowly emarginate on posterior margin (Fig. 6i, *t10*). Median lobe longer, sclerotized parts in dorsal view 1.6 times as long as wide. Median lobe in dorsal view narrowly unsclerotized medially only in anterior part, sclerotization medially fused in central part (Fig. 6f). Apex of median lobe narrowly sclerotized along median line (Fig. 6h).

#### Female genitalia

Tergite 8 without emargination anteriorly and with shallow emargination posteriorly (Fig. 7c, *t8*). Tergite 10 less elongate, only 1.1 times as long as wide posteriorly (Fig. 7c, *t10*).

### Variability

Body length varies between 22 and 49 mm. Elytra with variable colour pattern (Fig. 4e–k), as described above. Colour and size variation sometimes widely variable even in individuals from the same population. Similar variation in elytral pattern of *Necrodes surinamensis* (Fabricius, 1775) was described by Ratcliffe^30^.

### Taxonomy

The female holotype of *D. osculans* var. *reductus* falls within the intraspecific variation of *D. osculans*. The maculation on the elytra is less easily observed because the specimen is generally darkened, but the extent of the orange spot is similar to typical specimens. Consequently, we follow Růžička and Schneider^13^ who considered *D*. *osculans* var. *reductus* Pic, 1917 as a junior subjective synonym of *D. osculans* (Vigors, 1825).

*Necrodes bifasciatus* was only listed by Dejean^31^ atributed to Spinola and reported from Java. Following Bousquet and Bouchard^32^, all newly introduced species names in this work are unavailable, as they are only listed without any description.

*Diamesus osculans* ab. *diffusus* was introduced by Portevin^10^ to describe an individual variation in elytra colouration, when median part of elytron between anterior and posterior macula is brownish orange. This situation is generally present in subteneral adults. This name is not available, as it describes only individual colour aberration, and should be considered infrasubspecific.

### Bionomics

Necrophagous species, frequently collected on large carrion, with reported forensic significance (see below). However, adults of *Necrodes* feed primarily on fly larvae during the active decay stage of a carcass^30,33^, and the situation is probably similar for *Diamesus*. Most frequently collected using UV and mercury-vapour light traps^12^ (see SM1). According to Peck^12^, preferred habitats are rainforest, but also some disturbed or secondary open or closed canopy forest sites. Label data confirm the information from Peck^12^, that adults have been collected at elevations from sea level up to 1500 m in Papua New Guinea; we have seen records from altitude around 800 m in Australia: Queensland, 1500 m in Malaysia: Pahang, 1700 m in China: Guangdong and Thailand, 1800 m in Indonesia: Sumatra, over 1900 m in northern Vietnam, and even a single record at 2800 m in Nepal (see SM1). Seasonality: as the species is widely distributed from India to Australia, we select abundant available data from Bishop Museum Field Station, Wau, Papua New Guinea (ca. 07°19′S), to summarize the seasonality: available data confirm occurrence of adults in all months of the year, with peaks in March–April and August–October, with teneral adults evenly distributed throughout the year (Table 2). This is similar to pattern observed in Australia, again with the main peak in February–April and a smaller peak also in September–December^34^.

#### Defensive behaviour

Adult *D. osculans* in a laboratory colony were observed showing defensive behaviour—rapid movement, combined with ejection of a malodorous fluid from the mouth and anus, and even spraying it from the abdomen for a distance of several centimetres (J. Růžička & P. Jakubec, unpubl.). This is similar to the behaviour pattern described for *Necrodes surinamensis* by Ratcliffe^30^ and Eisner and Meinwald^35^. The chemical substances secreted by *N. surinamensis* were later identified as α-and β-necrodols, and their repellence was tested on ants and some other insects^36^. In *Necrodes*, these substances are probably used to monopolize the carrion and also to provide heat to increase beetle fitness^37^. The chemical composition of defensive substances in *Diamesus* is probably similar.

The orange colouration on the apices of antennae, elytra and abdominal tergites 3–4 in combination with dark brown to black body colour probably play a role as an aposematic signal warning predators that *Diamesus* is defended. As the colour pattern on tergites 3–4 is normally covered by the elytra in the resting position, we speculated that it is only exposed during flight. A similar bicoloured pattern of body dorsum was mentioned for other carrion beetles by Jones^38^ for *Nicrophorus* Fabricius, 1775 and Fisher and Tuckerman^39^ for *Necrophila* Kirby & Spence, 1828. No information is available for *Diamesus*. Future studies should also focus on this phenomenon of aposematic signaling.

#### Pollination

Two series of specimens from Indonesia (Sumatera Barat province: Palupuh and Bukittinggi) were found to be associated with Titan arum (*Amorphophallus titanum* (Becc.) Becc. ex Arcangeli (Araceae)). Titan arum is known to emit a unique rotting animal-like odour from its inflorescence that attracts insects for pollination; the odor consists of several sulphur-containing volatile organic compounds including dimethyl trisulfide (DMTS)^40^. DMTS is part of the odour of carrion and was also found to be attractive to two species of Central European *Nicrophorus* (Coleoptera: Staphylinidae: Silphinae) using electroantennography^41^. *Diamesus* is also mentioned as a pollinator of Titan arum in the review by Davis et al.^42^, where this association of carrion beetles and flies with Titan arum is called sapromyophily.

#### Phoresy

Halliday^43^ reported *Macrocheles agilis* Halliday, 2000 (Acari: Gamasida: Macrochelidae) as phoretic mite on *Diamesus osculans* in Papua New Guinea and Australia.

### Forensic significance

Wang et al.^44^, Eddie et al.^45^ and Magni et al.^46^ reported *D. osculans* on pig carrion in southern China, Malaysia and Western Australia, and briefly pointed out its forensic significance. Zhang et al.^15^ also mentioned *D. osculans* as a forensically important species in southern China. However, the larva of *Diamesus* is not described (J. Růžička et al. in prep.), and thermal summation models for its developmental stages are currently not available.

### References on distribution

**India:** “Indiâ Orientali” (Vigors 1825)^5^, South India (Arrow 1909)^47^; **Nepal:** Annapurna mts, Poon Hill, 28°34′N 083°50′E (Schawaller 2003)^48^; **Cambodia:** R’leak Korng Cherng village, 11°46.731′N 103°46.592′E (Sin et al. 2021)^49^; Prey Lang Wildlife Sanctuary, 13°14.705′N, 105°37.278′E (Sin et al. 2021)^49^; **China:** Guangdong province: Zhongshan, 22°31′N 113°22′E (Wang et al. 2008)^44^; Chaozhou [ca. 23°39′N 116°37′E] (Zhang et al. 2020)^15^; Chongqing province: Chongqing (Růžička et al. 2002)^50^; list of provinces, without precise localities (Ji 2012)^51^; **Taiwan:** Kôshun [= Pingtung County, Hengchun, ca. 22°00′N 120°45′E] (Miwa 1931)^29^, “Taiwan” (Ji 2012)^51^; **Thailand:** Chiang Mai, Doi Suthep [ca. 18°48′N 98°53′E] (Nishikawa 2014)^28^; **Japan:** Yaeyama Islands (Kurosawa 1974; Matoba 1975; Shoyama 2020)^52–54^; **Philippines:** *Luzon Island*: Imugan, Nueva Vicaya (Arnett 1950)^11^; *Cebú Island*: Bugó [= Bogo, ca. 11°03′N 124°00′E] (Arnett 1950)^11^; **Malaysia:** Sarawak (Arrow 1909)^47^; Sabah: Tombongon [ca. 06°00′N 116°14′E] (Eddie et al. 2016)^45^; **Indonesia:** Papua Barat and Papua provinces [6 unnamed localities] (Peck 2001^12^: fig. 11); **Papua New Guinea:** Woodlark Is. [= Muyua Island] (Arrow 1909)^47^; New Britain Island: Gazelle Peninsula, Yalom [ca. 04°25′S 151°45′E] (Mroczkowski 1966)^55^; Wau [ca. 07°20′S 146°43′E] (Halliday 2000)^43^; Amboin [ca. 04°37′S 143°29′E] (Halliday 2000)^43^; Erap [ca. 06°32′S 146°42′E] (Halliday 2000)^43^; [12 unnamed localities] (Peck 2001^12^: fig. 11), **Australia:** Queensland (Arrow 1909)^47^; Northern Territory, Mudginberri [ca. 12°37′S 132°52′E] (Halliday 2000)^43^; Northern Territory, Cape Crawford, 16°34′S 135°41′E (Halliday 2000)^43^; Queensland, Kirrama Ra. [ca. 18°06′S 145°42′E] (Halliday 2000)^43^; Queensland, 15 km S Biloela [ca. 24°32′S 150°31′E] (Halliday 2000)^43^; Queensland, Binna Burra [ca. 28°11′S 153°11′E] (Halliday 2000)^43^; Queensland, Ingham [ca. 18°39′S 146°09′E] (Halliday 2000)^43^; New South Wales, Huonbrook [ca. 28°32′S 153°23′E] (Nishikawa 2014)^28^; New South Wales, Bonville [ca. 30°23′S 153°04′E] (Halliday 2000)^43^; [51 unnamed localities] (Peck 2001^12^: fig. 11).

### Distribution

Widely distributed species, known from India (Assam, Karnataka, Kerala, Meghalaya, Sikkim, Tamil Nadu, Uttarakhand and West Bengal), Sri Lanka, Nepal, Bhutan, China (Anhui, Chongqing Municipality, Fujian, Guangdong, Guangxi Autonomous Region, Hainan, Hunan, Jiangxi, Shaanxi, Xizang (Tibet) Autonomous Region, Yunnan and Zhejiang), Taiwan, Japan (Ryukyu Islands: Iriomote-jima Is. and Ishigaki-jima Is.), Myanmar, Thailand, Laos, Cambodia, Vietnam, Philippines, Malaysia (Johor, Kedah, Malacca, Pahang, Perak, Sabah and Sarawak), Brunei, Indonesia (Aceh, Bali, Bengkulu, Jakarta, Jawa Barat, Lampung, Maluku, Nusa Tenggara Barat, Nusa Tenggara Timur, Papua, Papua Barat, Riau, Sulawesi Selatan, Sulawesi Tengah, Sulawesi Utara, Sumatera Barat, Sumatera Selatan and Sumatera Utara), Papua New Guinea, Solomon Islands and Australia (Australian Capital Territory, New South Wales, Northern Territory, Queensland, Victoria and Western Australia). First records from India: Uttarakhand State, China: Jiangxi and Shaanxi Provinces and Xizang (Tibet) Autonomous Region, and Australia: Victoria (Fig. 8a,b).

## Discussion

### Forensic significance

From anecdotal evidence based on locality data it seems that the ecology of both species of the genus *Diamesus* is similar to their close relatives from the genus *Necrodes*, which breeds on dead bodies of larger vertebrates and can also be attracted to light^33,56^. This is in line with field observations and experiments where typically *D. osculans* was present (see^12,46,49,57^). Like other genera of the subfamily Silphinae e.g., *Necrodes*^58,59^, *Necrophila* Kirby & Spence, 1828^60^*, Oxelytrum*^61^ or *Thanatophilus*^4,62^, species of the genus *Diamesus,* therefore might be used in forensic entomology to estimate time of colonization, which is crucial information for medico-legal casework^63^. This is further supported by observations of the species (*D. osculans*) feeding as adults and larvae on human corpses^64^.

### Distribution

Peck^12^ summarized the distribution of *D. osculans* in Eastern Australia, and commented on the absence of records of this species from Australia in Portevin^10^ and speculations of introduction into northern Queensland in Tillyard^65^. He also mentioned an imprecisely localized specimen from “Australia” collected in 1896 and other specimens collected in 1909 (Queensland: Cairns) and before 1917 (Queensland: Normanton). The collection of J. Kořenský (NMPC) houses two additional specimens from Melbourne and “Australia” (see SM1). According to Kořenský^66^, he visited Australia and collected insects there in 1900–1901. All these findings support the idea of the autochthonous occurrence of *D. osculans* in Australia.

*Diamesus osculans* was already listed from Taiwan by Miwa^29^. These beetles may be based on specimens from the “Shiraki specimens”, which includes non-Taiwanese material, labelled with added fake Taiwanese localities^67–70^. This was ignored in recent catalogues^13,14^ along with a series of other clear misidentifications of carrion beetles reported from Taiwan by Miwa^29^. However, recent data on occurrence of both *D. bimaculatus* and *D. osculans* show that both species live in sympatry in Taiwan. Fang-Shuo Hu (pers. comm.) mentioned that *D. osculans* is rarely collected in Taiwan, compared to *D. bimaculatus*, which is much more abundant. Future research should study in detail possible competition between the two species of *Diamesus*, and examine if the reason for the relative rarity of *D. osculans* on Taiwan can be explained by repeated invasions or unintentional introductions of *D. osculans* from mainland Asia, or if there is an established population in Taiwan. The species is present on even quite remote and isolated islands in Indonesia, Papua New Guinea, and the Solomon Islands (Fig. 8a, SM1).

Similarly, there are only four collecting records (in three short communications) on *D. osculans* from two islands (Iriomote-jima Is. and Ishigaki-jima Is.) of Yaeyama Islands, in the most south-western part of Ryukyu Islands, Japan^52–54^. Most probably, *D. osculans* has not established stable population there, and these specimens collected on Yaeyama Islands were unintentionally introduced by human activities (S. Nomura, pers. comm.). However, the occurrence of *D. osculans* in this region is also predicted by the MaxEnt model. Similar case of a recent introduction of *Cicindela batesi* Fleutiaux, 1894 from Taiwan to Iriomote-jima Is. and *C. chinensis* *okinawana* Nakane, 1957 (both Coleoptera: Carabidae) from Okinawa-jima Is. to Ishigaki-jima Is. is described by Osozawa et al.^71^ Other exotic insect species from Philippines, Indochina or even New Guinea were accidentally found in the Yaeyama Islands, but these appear to have not established permanent populations (S. Nomura, pers. comm.).

### Species distribution models

These models mostly agree with the published works of Portevin^10^ and Peck^12^. The model for *D. bimaculatus* did not find it plausible that the species would occur outside of Taiwan. On the other hand, the presence of *D. osculans* on Tasmania and New Caledonia was suggested. Both islands were investigated in the past by a number of entomologists without recording the species, and we think its presence there is very unlikely due to its size and tendency to occur in large numbers on cadavers and its ease of being attracted to lights, including light traps. The model simply suggest that suitable conditions are present on these islands without taking into consideration the past biogeography and distance from the nearest source population.

The species distribution model of *D. osculans* suggested a possible presence of the species in parts of the World where it was so far not reported, or the reports were not considered as likely. In the last category certainly belongs Japan, where the species was observed only on the south-west part of Ryukyu Islands, but the models suggest occurrence also on Yakushima Is. Other parts where we consider the presence of the species less likely is Vanuatu. This archipelago is separated by large water bodies and its fauna is relatively well known, so the presence of a large necrophagous beetle would surely be recorded. On other hand, presence of the beetle on Tasmania could be possible as well as on other small islands in the Maritime Southeast of Asia due to their position close to previously confirmed populations of the species.

### Phylogeny

The molecular phylogeny strongly agrees with the previously published phylogenies of the subfamily Silphinae and the tribe Silphini^1,23,72,73^. However, this is the first analysis including both species of the genus *Diamesus*. The phylogenetic analysis confirmed the monophyly of the genus *Diamesus* and supports the genus as a sister lineage to *Necrodes*. The specimens of *Diamesus osculans* used for the phylogenetic analysis represent populations from a wide distribution area, as well as the specimens of *D. bimaculatus* collected in Taiwan, which remains its only known area of distribution.

Plate tectonics models suggest that colonization pathways from Asia to Australia could occur approximately only in the last 10 mya^74^. Also, according to these models, the occurrence of *D. osculans* on the islands of Sumatra, Java, Borneo and others might be relict from this time period as these islands were probably repeatedly interconnected with continental Asia^74,75^. Suggesting, that the clade consisting of the genera *Necrodes* and *Diamesus* originated in the northern hemisphere. This idea is further supported by the existence of a Beringian land bridge between Asia and North America approximately ca. 65.5 and ca. 58 mya when the colonization of North America by *Necrodes* might have occurred^76^.

*Diamesus osculans* show generally only small intraspecific variation in compared sequences, based on individuals collected throughout its range. This, in combination with phylogenetic analysis can suggest quite high historical genetic connectivity between separate populations within its range.

### Estimation of the divergence time

The estimated split of both species of *Diamesus* (Palaeocene–Eocene, 26.9–54.1 mya) is much older than the proposed appearance of Taiwan as a continental island, emerging above sea level only at the Miocene-Pliocene boundary (ca. 5 mya), owing to the collision of the Philippine Sea plate and the Eurasian plate^77^. Obviously, endemic occurrence of *D. bimaculatus* on Taiwan can be relictual, and the original distribution range could have been much broader, with extirpation throughout its former range.

## Material and methods

### Specimen sampling, museum abbreviations

Specimens examined in this study were loaned from the following 52 museums and private collections (acronyms listed according to Arnett et al.^78^):

AMNH: American Museum of Natural History, New York, U.S.A. (L. Herman)
BHHC: collection of Bin-Hong Ho, Taipei, Taiwan
BMNH: Natural History Museum, London, United Kingdom (M.V.L. Barclay)
BPBM: Bernice Pauahi Bishop Museum, Honolulu, Hawaii, U.S.A. (†J. Boone, N. Evenhuis)
CAS: California Academy of Sciences, San Francisco, California, U.S.A. (D. Kavanaugh)
CAU: China Agricultural University, Beijing, China (Xin-Li Wang)
CMNH: The Carnegie Museum of Natural History, Pittsburgh, Pennsylvania, U.S.A. (R.L. Davidson)
CYTC: collection of Cheng-Yan Tu, Taipei, Taiwan
DSSC: collection of Derek S. Sikes, Fairbanks, U.S.A.
FMNH: Field Museum of Natural History, Chicago, U.S.A. (A.F. Newton, M. Thayer)
FSHC: collection of Fang-Shuo Hu, Yilan, Taiwan
HNHM: Magyar Természettudományi Muzeum, Budapest, Hungary (†O. Merkl, Gy. Makranczy)
IRSNB: Institut royal des Sciences naturelles de Belgique, Bruxelles, Belgium (W. Dekoninck, A. Drumont)
IZ-CAS: Institute of Zoology, Chinese Academy of Sciences, Beijing, China (Hong-Zhang Zhou)
JCLC: collection of Jincheng Liu, Beijing, China
JHAC: collection of Jiří Háva, Prague, Czech Republic
JRUC: collection of Jan Růžička, Prague, Czech Republic
JSCC: collection of Jan Schneider, Prague, Czech Republic
JVAC: collection of Jiří Vávra, Ostrava, Czech Republic
KHAC: collection of Keitaro Harusawa, Osaka, Japan
LDVC: collection of Libor Dvořák, Tři Sekery, Czech Republic
MHNG: Muséum d’Histoire Naturelle, Genève, Switzerland (G. Cuccodoro)
MNHN: Museum national d’Histoire naturelle, Paris, France (Azadeh Taghavian)
MNIC: collection of Masaaki Nishikawa, Ebina, Japan
MZLU: Biological Museum, Lund University, Lund, Sweden (Ch. Fägerström)
MZSP: Museu de Zoologia da Universidade de São Paulo, São Paulo, Brasil (†U.R. Martins)
NHMB: Naturhistorisches Museum, Basel, Switzerland (Eva Sprecher-Uebersax, M. Borer)
NHMW: Naturhistorisches Museum, Wien, Austria (H. Schillhammer)
NHRS: Naturhistoriska riksmuseet, Stockholm, Sweden (J. Bergsten)
NMNH: National Museum of Natural History, Smithsonian Institution, Washington, U.S.A. (F. Shockley, G.F. Hevel)
NMNS: National Museum of Natural Science, Taichung City, Taiwan (Jing-Fu Tsai)
NMPC: Národní museum, Prague, Czech Republic (J. Hájek, M. Fikáček)
NSMT: National Museum of Nature and Science, Tsukuba, Japan (S. Nomura)
RMNH: Naturalis Biodiversity Center, Leiden, Netherlands (A. von Assen)
ROM: Royal Ontario Museum, Toronto, Canada (C. Darling)
SDEI: Senckenberg Deutsches Entomologisches Institut, Müncheberg, Germany (Marianna Simões, L. Behne)
SEMC: Shanghai Entomological Museum, Chinese Academy of Sciences, Shanghai, China (Hai-Sheng Yin)
SHNU: Department of Biology, Shanghai Normal University, China (Liang Tang, Zi-Wei Yin)
SMFD: Forschungsinstitut Senckenberg, Frankfurt am Main, Germany (Andrea Vesmanis, D. Kovac)
SMNS: Staatliches Museum für Naturkunde, Stuttgart, Germany (W. Schawaller, A. Faille)
SMTD: Museum für Tierkunde, Dresden, Germany (O. Jäger)
SNUC: Insect Collection of Shanghai Normal University, Shanghai, China (Liang Tang, Zi-Wei Yin)
SYSU: Institute of Entomology, Sun Yat-sen University, Guangzhou, China (Feng-Long Jia)
UCDC: The Bohart Museum of Entomology, University of California, Davis, U.S.A. (S. Heydon)
WBAC: collection of Wolfgang Barries, Wien, Austria (deposited in NHMW)
YFTC: Yunnan Forestry Technological College, Kunming, China (Guo-Feng Li)
YJIC: collection of Yun Ji, Beijing, China
ZMAN: Zoölogisch Museum Amsterdam, Amsterdam, the Netherlands (joined and transferred to RMNH)
ZMAS: Zoological Museum, Academy of Sciences, St. Petersburg, Russia (M.G. Volkovitsh)
ZMHB: Museum für Naturkunde, Berlin, Germany (J. Frisch, B. Jäger)
ZMUC: Zoological Museum, University of Copenhagen, Copenhagen, Denmark (A. Solodovnikov)
ZYCC: collection of Zhen-Yi Chen, Taipei, Taiwan

Exact label data are cited only for the type material, using the following set of abbreviations: coll. —collection of (not collector); leg.—collected by; MS—manuscript, HT—holotype, LT—lectotype, PLT—paralectotype(s). Authors’ remarks and addenda are enclosed in square brackets; [p]—the preceding data are printed; [hw]—preceding data are hand-written. Separate lines on labels are indicated (only for primary types) by “/”, separate labels by “//”. The lectotype and paralectotypes of *D. osculans* var. *bimaculatus* are designated in order to preserve stability of nomenclature in this group, according to the Article 74.7.3 of the Code^26^.

The following abbreviations are used for the determiners of the material: AFN—Alfred F. Newton, DSS—Derek S. Sikes, JH—Jiří Háva, JS—Jan Schneider, KH—Keitaro Harusawa, MN—Masaaki Nishikawa, SBP—Stewart B. Peck, WB—Wolfgang Barries, WS—Wolfgang Schawaller. If otherwise not mentioned, Jan Růžička determined or revised the material of adult beetles.

### Morphological analysis

The morphological terminology used in this paper follows Lawrence and Ślipiński^79^. Hind wing venation was homologised following Kukalová-Peck and Lawrence^80^ and Lawrence et al.^81^. Male and female terminalia of *Diamesus* were studied after short clearing in a hot 10% solution of KOH and photographed submerged in alcohol, using a Canon macro photo lens MP-E 65 mm or EF-S 60 mm on a Canon 750D. Habitus pictures of adults (incl. types) and details of wings were based on photography of dry, manually cleaned specimens, using the same setup. For each structure, multiple layers of focus were combined in the Zerene Stacker 1.04 software (http://www.zerenesystems.com/cms/stacker). Other morphological characters were measured and documented using a Keyence VHX-6000 digital microscope.

To observe fine structures on female genitalia, we used a scanning electron microscope. Preparation of samples follows the methodology of Novák et al.^62^. Selected samples were dehydrated using a graded series of ethanol (75%, 80%, 90%, 95%, 100%) and left in each concentration for approximately 30 min. before transferring to acetone overnight. Dehydrated samples were dried using the critical point drying method. Dry samples were then attached to an aluminium disk target using copper foil tape and coated with gold in Bal-Tec Sputter Coater SCD 050. Samples were observed and documented with a JSM-6380LV (JEOL) scanning electron microscope.

All pictures were digitally enhanced using Adobe Photoshop CS4 or CorelPHOTO-PAINT 2018, plates were arranged in CorelDRAW 2018.

### Measurements

Morphological characters were measured using a software available in Keyence VHX-6000 digital microscope. The following measurements were taken: body length, pronotum: median length and maximum width, scutellum: length and width, elytra: length (measured as length from posterior margin of scutellum to perpendicular distance of apex of both elytra) and combined width of both elytra. Body length was measured with precision of 1 mm, the other measurements with precision of 0.1 mm. In total, 11 males and 12 females of *D. bimaculatus* and 13 males and 12 females of *D. osculans* were measured, individuals were selected from dry mounted material to cover maximum variation in body size. For *D. bimaculatus*, mostly lectotype and paralectotypes were measured; in *D. osculans*, all non-type specimens are vouchers deposited in JRUC. Measured data are available in SM4.

We used linear regression with normal distribution of errors to model the effect of species identity and sex on four major morphological characteristics, body length, elytra width to length ratio, pronotum width to length ratio and scutellum length to width ratio. The significance level was set at 5%. Data management and all analyses were carried out using the R statistical program ver. 4.1.1^82^. Graphical outputs were created using ggplot2^83^.

### Maps

Localities were interpreted from locality labels, unified (except primary types) and georeferenced with the help of several gazetteers and map sources: Microsoft Encarta Premium 2008^84^, NGA GEOnet Names Server^85^, Google Earth Pro^86^ and during the initial phase, also Fuzzy Gazetteer^87^. For large cities without precise locality data, just centroids are reported in SM1 and used in distribution maps. These data were combined with published records of Peck^12^, from Papua, Papua New Guinea and Australia (in total, 68 unnamed localities). These were georeferenced from map 11, as published by Peck (2001: 97)^12^. Additionally, distribution data of *Diamesus* (119 observations, downloaded on 26 April, 2021) were mined from iNaturalist^88^, all records were verified by J.R., based on habitus pictures available for each record. Additional five observations of *D. osculans* from Taiwan were downloaded from iNaturalist on 23 October, 2022.

The distribution maps were produced by ESRI ArcMap 10.8.1 of ArcGIS Desktop 10.8.1 suite. For map layers, free level 0 data from Global Administrative Areas (http://www.gadm.org, ver. 2.8), Natural Earth (http://naturalearthdata.com, Natural Earth I with Shaded Relief, Water, and Drainages) (with 30% transparency and 15% brightness) over World Shaded Relief (https://www.arcgis.com/home/item.html?id=9c5370d0b54f4de1b48a3792d7377ff2) were used.

### Species distribution models

Species distribution models (SDM) were established using the machine learning algorithm MaxEnt^89^ for both species (*D. bimaculatus* and *D. osculans*) based on presence only occurrence data. As explanatory variables we used values of four bioclimatic shapefiles (WorldClim 2.1)^90^ (annual mean temperature (Bio 1), mean diurnal range (Bio 2), temperature annual range (Bio 7), and precipitation of warmest quarter (Bio 18)) and map of terrestrial ecoregions of the World^91^. Due to large disparities between distribution ranges for both species, we used two resolutions of the explanatory shapefiles, 30 arc seconds for *D. bimaculatus* and 2.5 arc minutes for *D. osculans*. The quality of the models was assessed using area under curve (AUC) metric and resulting SDM models were binomially reclassified based on maximum training sensitivity plus specificity Cloglog thresholds.

### Molecular analysis

DNA was extracted from hind-leg muscle tissue of specimen stored in 96% EtOH and dry specimens using commercial kit Geneaid Tissue & Blood Kit (Geneaid, New Taipei City, Taiwan) following provided protocol. Three genes were amplified—cytochrome oxidase I (COI, mtDNA), 16S (rDNA) and 28S (nDNA) using previously published C1-J-2183 (alias Jerry) & TL2-N-3014 (alias Pat)^92^, LR-J-12887 (alias 16Sbr) & LR-N-13398 (alias 16Sar)^92^, and Rd3.2a & Rd5b^93^. The PCR reactions were carried out at 25 µl based on provided PPP Master Mix protocol (Top Bio) (12.5 µl of 1 × PPP Master Mix, 9.5 µl PCR H_2_O, 0.4 μM of forward and 0.4 μM reverse primer) under the conditions shown in Table 3. PCR products were visualized by electrophoresis on 1% agarose gel and subsequently purified using ExoSAP-IT (Applied Biosystems) following the protocol provided, bi-directional sequencing was carried out in BIOCEV (Vestec, Czech Republic) using the Sanger sequencing method with the same primers as for the amplification.

**Table 3:** Cycling conditions used in the presented study.

Step	COI (Jerry and Pat)	16S rDNA (16Sar and 16Sbr)	28S rDNA (Rd3.2a and Rd5b)
Initial denaturation	95 °C for 3 min	95 °C for 3 min	95 °C for 3 min
Number of cycles	40	40	40
Denaturation	95 °C for 30 s	95 °C for 30 s	95 °C for 30 s
Annealing	50 °C for 30 s	47 °C for 30 s	56 °C for 30 s
Extension	72 °C for 45 s	72 °C for 45 s	72 °C for 45 s
Final extension	72 °C for 10 min	72 °C for 10 min	72 °C for 10 min

### Phylogenetic analysis

Obtained sequences for each gene were visualised in Chromas v2.6.6 (Technelysium Pty Ltd, Brisbane, Australia), visually compared, and trimmed. The newly generated sequences were deposited in GenBank (http://www.ncbi.nlm.nih.gov) and combined with previously published sequences from the GenBank database (http://www.ncbi.nlm.nih.gov) (Table 1). Alignment was generated using MAFFT^94^ using Guidance2 server^95^. Alignment was visualized, manually edited, and trimmed to an equal length in BioEdit v7.0.5.3^96^. Prior to analysis, each gene alignment was checked for the best fitting model, using ModelFinder^97^ implemented in IQ-TREE webserver^98^ separately according to the Akaike Information Criterion—COI GTR + F + I + G4, 16S K3Pu + F + I + G4, and 28S GTR + F + I + G4. Subsequently, the alignments were concatenated using software MEGA-X v10.1.8.^98^. For phylogeny reconstruction the Maximum Likelihood (ML), Maximum Parsimony (MP), and Bayesian Inference (BI) methods were applied. The ML method was conducted in IQ-TREE webserver^99^ under 1000 Ultrafast bootstrap (UFBoot)^100^ iterations. The BI method was performed in MrBayes v3.2.7^101^ (mcmcp ngen = 10,000,000, samplefreq = 1000, printfreq = 1000, nchains = 4, savebrlens = yes, nst = 6 rates = invgamma), first 25% of trees were discarded. The MP method was conducted in PAUP* v4.0a169^102^ nreps = 10, swap = TBR nchuck = 100 chuckscore = 1, bootstrap nreps = 10,000, search = heuristic), results were visualised in FigTree v1.4.4^103^. The concatenated alignment and generated trees of each method are available at https://github.com/KarolinaMahlerova/Alignment-and-phylogenetic-trees-Diamesus_.

### Estimation of the divergence time

The estimation of the divergence time of the selected genera of the subfamily Silphinae was conducted using BEAST2 software v2.6.6.0^104^. The concatenated alignment of mitochondrial genes COI and 16S of 36 individuals (excluding *Necrodes surinamensis* from the original dataset due to missing sequence of 16S) was used. Based on previously published divergence time estimates of the subfamily Silphinae^105,106^, the following parameters were set using BEAUti2 v2.6.6.0^104^: GTR substitutional model, frequencies: All Equal, Clock Model: Relaxed Clock Log Normal, Chain Length 10,000,000, and sampled every 1000 generations to generate .xml input file for estimation of the divergence time. Three priors with normal distribution were used for calibration of the model—Silphinae 175 mya (184.9–165.1; sigma 0.95)^105,107^, Nicrophorini 135 mya (144.9–125.1; sigma 0.95)^105,108^, and *Necrodes* 48.25 mya (53.0–47.5; sigma 0.95) (fossil from Green River formation; J. Růžička, unpublished), 25% of trees was discarded as burn-in. To assess the convergence Tracer v1.7.2 was used. TreeAnnotator v2.6.6.0 was used to generate maximum clade credibility tree, median ages and their 95% highest posterior density. The output file was visualised in FigTree v1.4.4^103^.

## Supplementary Information

Supplementary Information 1.

Supplementary Information 2.

Supplementary Information 3.

Supplementary Information 4.

## Data Availability

We provide following data used in our study—list of examined non-type material of *Diamesus* (SM1) and georeferenced data used for production of distribution map and species distribution models (SM2), and raw data of measured structures of external morphology of *Diamesus* (SM3).
