# Supplementary material for: Integrative taxonomy and species distribution models of the genus Diamesus Hope, 1840 (Coleoptera: Staphylinidae: Silphinae)
Source: Sci Rep. 2023 Feb 23;13:3192. doi: 10.1038/s41598-023-30019-x (PMC9950127; doi:10.1038/s41598-023-30019-x)
Supplement: Supplementary file 1 — Supplementary Information 1. [file 41598_2023_30019_MOESM1_ESM.pdf]

SM1. List of examined additional (non-type) *Diamesus* material. For collection acronyms, see Material and methods.

Diamesus bimaculatus **Portevin, 1914** (87 specimens)

Taiwan: Taipei City: Pittu [probably Shicao Village (石嘈里)], 24°54.0'N 121°45.5'E, 450 m, 4.–5.iv.1997, L. Peregovits & A. Kun leg., 1 male (HNHM);

Taipei City: “Formosa, Taihoku” [= Taipei], 4.viii.1927, F.C. Hadden leg., 1 female (BPBM);

Taoyuan City: 14 km E Fuhsing [= Fuxing (復興區) env., probably Xiayun Village (霞雲里)], 24°50'N 121°23'E, 800 m, 18.v.1995, M. Hreblay & P. Stéger leg., 1 female (HNHM);

Taoyuan City: Fuxing Township, Dongyan Mt. [東眼山, ca. 24.825°N 121.417°E], 22.viii.2020, H. Ho leg., light trap, Fang-Shuo Hu det., 1 male, 1 female (FSHC);

Taoyuan City: Paling [= Baling (巴陵), ca. 24°40'N 121°23'E], 17.vii.1992, J.J. Chen leg., KH det., 1 female (KHAC); Baling, 24°39.23'N 121°24.38'E, 863 m, 27.iv.2016, light trap, E. Ruzzier & A. Serafin leg., 2 females (JRUC);

Taoyuan City: Szuling [= Siling (四稜), ca. 24°39'N 121°25'E], 17.vii.1993, N. Okuda leg., KH det., 1 female (KHAC);

Hsinchu county: Chienshih [= Jianshi Township (尖石鄉), ca. 24°34'N 121°19'E], 26.vii.1992, J.J. Chen leg., KH det., 1 male (KHAC);

Hsinchu county: Xin-Zhu [= Hsinchu (新竹), ca. 24°48'N 120°58'E], 26.i.2003, H. Wenbe leg., 1 male, 1 female (BMNH);

Hsinchu county: Baoshan [(寶山), ca. 24°45'N 121°00'E], 29.v.1993, W.I. Chou leg., 1 male (NMNS);

Yilan county: Paling env., Chituan [= Chiduan (池端), ca. 24°39'N 121°28'E], 8.vii.1992, J.J. Chen leg., at light, KH det., 1 male, 2 females (KHAC); same locality, 22.viii.1992, J.J. Chen leg., at light, KH det., 1 male, 1 female (KHAC); same data, 2 males, 2 females (JRUC); same data, 1 male, 1 female (JSCC);

Yilan county: Fu Shan Botanical Garden [= Fushan Botanical Garden (福山植物園)], LTER site, 24°45.5'N 121°35.8'E, 700 m, 4.–7.viii.1999, A. Kun leg., 1 female (HNHM); same locality, 700 m, 25.–27.ix.2000, L. Papp, L. Peregovits & L. Ronkay leg., at light, 2 females (HNHM); same locality, 24°45.361'N 121°35.761'E, 16.ix.2007, D. Redei & J.-F. Tsai leg., 1 female (HNHM);

Yilan county: Mingchyr [= Mingchi (明池)], 24°39'N 121°28'E, 1150 m, 11.–12.ix.1999, G. Csorba & B. Herczig leg., at light, 1 female (HNHM);

Miaoli county: 21 km E Tungshih [= Dongshi District (東勢), ca. 24°15'N 120°55'E], 1335 m,

22.iii.1996, T. Csővári & P. Stéger leg., 1 female (HNHM);

<u>Taichung City</u>: Chingshan [青山, ca. 24.235°N 121.085°E], 2.ix.1987, I.S. Hsu leg., sweeping net, 1 male (NMNS); same locality, 9.–10.ix.1993, W.T. Yang & M.L. Chan leg., UV light, 1 female (NMNS);

<u>Taichung City</u>: 25 km N Puli [埔里], Nisun forest area [= Huisun Forest Area, ca. 24°05'N 121°02'E], 500 m, 9.vii.1996, G. Csorba & L. Nemetu leg., 1 female (HNHM);

<u>Nantou county</u>: Riyuedan [= Sun Moon Lake (日月潭), ca. 23°51'N 120°54'E], 23.x.1971, K. Masumoto leg., MN det., 1 spec. (MNIC); Sun Moon Lake, 5.–6.v.1973, S. Tsuyuki leg., 1 female (NSMT);

<u>Nantou county</u>: Nanshanxi [= Nanshanxi (南山溪), ca. 24°01'N 121°05'E], 6.vi.1972, Q.-L. Du leg., MN det., 1 spec. (MNIC);

<u>Nantou county</u>: Lushan spa [廬山溫泉, ca. 24°02'N 121°11'E], 1200 m, 9.viii.1984, A. Kanbe leg., MN det., 1 spec. (MNIC); Lu Shan, 9.vii.2007, A. Kun leg., 1 male (HNHM);

<u>Nantou county</u>: 16 km E Kuohsing [= Guoxing (國姓)], Ursun F.[orest?] [= Huisun Experimental Forest Station (惠蓀林場)], 24°05'N 121°00'E, 560 m, 21.viii.1996, T. Csővári & L. Mikus leg., 1 female (HNHM);

<u>Nantou county</u>: 3 km E Tili [地利], 23°47'N 120°58'E, 555 m, 17.iii.1996, T. Csővári & P. Stéger leg., 1 female (HNHM); same locality, 31.iii.1996, 1 female (HNHM); same data, 1 male (JRUC);

<u>Nantou county</u>: Mt. Hohuangshan [= Mt. Hehuan (合歡山), ca. 24°10'N 121°17'E], 1600 m [erroneous?, should be around 3000 m (Fang-Shuo Hu, pers. comm.)], 23.vii.1998, N. Okuda leg., KH det., 1 male, 1 female (KHAC);

<u>Nantou county</u>: Jenai [= Jenai Township (仁愛鄉)], Meifeng [Mt. (梅峰), ca. 24°05'N 121°10'E], 30.viii.1990, C.S. Lin leg., UV light trap, 2 males (NMNS); same locality, 10.–12.vi.1996, C.S. Lin & W.T. Yang leg., 1 male (NMNS);

<u>Nantou county</u>: Jenai [= Jenai Township (仁愛鄉)], Huishun [= Huisun (惠蓀), ca. 24.091°N 121.031°E], 27.vi.–1.vii.1994, W.T. Yang leg., mercury light, 1 male (NMNS);

<u>Nantou county</u>: Wushe [霧社, ca. 24°02'N 121°07'E], 4.vii.1991, C.S. Lin leg., UV light trap, 1 male (NMNS); same locality, 30.ix.1992, C.S. Lin leg., UV light, 2 males (NMNS);

<u>Nantou county</u>: Chunyan [= Chunyang (春陽), ca. 24°01'N 121°09'E], 27.–29.vii.1993, C.S. Lin & W.T. Yang leg., UV light, 2 males (NMNS); Jenai [= Ren'ai env.], Chunyan, 7.–9.ix.1998, C.S. Lin leg., mercury light, 1 male (NMNS);

<u>Nantou county</u>: Luku [= Lugu Township], Hsitou observatory [= Xitou (溪頭), ca. 23°40.4'N 120°47.8'E], 20.–21.ix.2006, Chan, Lan, Liang, Wu & Huang leg., mercury light, 1 female

(NMNS);

Kaohsiung City: Kosempo [= Chiahsien or Jiaxian District (甲仙), ca. 23°05'N 120°35'E], vii.1911, H. Sauter leg., 2 females (ZMHB);

Kaohsiung City: 10–11 km NE Chiahsien [= Jiaxian (甲仙), ca. 23°10'N 120°39'E], ca. 300 m, 3.–8.vii.1980, D.R. Davis leg., forest, 1 male (NMNH);

Kaohsiung City: Lukuei, Tunchi [= Tungzhi or Tengzhi (藤枝), ca. 23°04'N 120°45'E], 6.–8.ix.1989, C.S. Lin leg., UV light, 1 male, 1 female (NMNS);

Kaohsiung City: Liukuei [= Liugui District (六龜), ca. 23°00'N 120°38'E], 17.viii.1986, W. Chen leg., MN det., 1 spec. (MNIC);

Kaohsiung City: Kaohsiung env., Tenghsi [not located], 10.vii.1983, W.-L. Chen leg., MN det., 1 spec. (MNIC);

Kaohsiung City: Shanping [扇平, ca. 22°58'N 120°41'E], 640 m, 1.–10.iv.1988, R. Davidson, J. Rawlins & C. Young leg., 1 male (CMNH); same data, 11.–20.iv.1988, leg., 4 males, 2 females (CMNH); same locality, 21.–30.iv.1988, C. Young, R. Davidson & J. Rawlins leg., 2 males (CMNH); same locality, 750 m, 11.–15.iv.1988, Lin & Huang leg., light trap, 1 female (NMNS); Shanpin [= Shanping], 750 m, 18.–23.iv.1988, Lin & Huang leg., light trap, 1 female (NMNS); same locality, 4.ix.1989, C.C. Chiang leg., sweeping net, 1 male (NMNS); same locality, 4.–6.ix.1989, K.W. Huang leg., light trap, 1 male (NMNS);

Kaohsiung City: 15 km SE Shanping, near Tuona [= Duona (多納), ca. 22°55'N 120°43'E], 1050 m, 12.–13.v.1988, R.L. Davidson leg., 1 male (CMNH);

Kaohsiung City: Tengchih [天池, ca. 23.277°N 120.915°E], 7.ix.1989, C.C. Chiang leg., sweeping net, 1 male (NMNS); Taoyuan [district], Tengchih, 9.viii.2004, W.C. Tsao leg., on light, 1 female (NMNS);

Kaohsiung City: Paosan [= Baoshan, 寶山, ca. 23.038°N 120.708°E], 11.–15.x.1988, C.S. Lin leg., UV light trap, 1 female (NMNS);

Taitung county: 7 km N Tupan [土坂], 22°29'N 120°52'E, 500 m, 20.iii.1996, T. Csővári & P. Stéger leg., 1 female (HNHM); same data, 1 female (JRUC);

Pingtung county: Taiwu Township, Xinzhi Mt. (新置山), 22.547027°N 120.644868°E, 15.iv.2017, Cheng-Yan Tu leg. & det., 2 spec. (CYTC);

Pingtung county: Kenting Botanical Garden [= Kenting Forest Recreation Area (墾丁森林遊樂區), ca. 21.959°N 120.811°E], 260 m, 22.–25.vii.1980, D.R. Davis leg., subtropical forest, 1 male (NMNH);

Pingtung county: Kontei Park [= Kenting National Park], 22.v.1984, S. Yoshimatso leg., 1 male (NSMT);

Not or imprecisely located: “Formosa, 3”, without date, T. Kano leg., 1 female (NSMT); Titsugatsutau [not located], 4.vii.1978, K. Akiyama leg., 4 males, 3 females (NSMT);

***Diamesus osculans* (Vigors, 1825)** (2173 specimens)

India: Uttarakhand State: Bhim Tal [ca. 29°21'N 079°33'E], 1500 m, 28.vii.2000, N. Delahaye leg., JH det., 1 male (JHAC);

Sikkim state: without more detailed locality [centroid 27°20'N 088°37'E] and collector’s name, 1920, 1 female (JRUC); without more detailed locality, date and collector’s name, 1 female (SMTD); same data, 1 male (SDEI);

Assam state: Brahmaputra river, 120 v. [= verst, ca. 128 km] above Gaukhati [= Guwahati, ca. 26°36'N 092°48'E], 12.i.1912, von Vik leg., 1 female (ZMAS);

Assam state: Nambor Reserve Forest, Garampani, 26°30'N 093°55'E, 100 m, 21.–29.xi.1997, V. Siniaev & V., S. & M. Murzin leg., JS det., 2 males, 2 females (JSCC);

West Bengal state: Darjeeling [ca. 27°02'N 088°15'E], without date [but before 1965], without collector’s name, ex coll. Otterstedt, 1 female (SMTD); same locality, without date and collector’s name, 1 female (NHRS);

West Bengal state: Darjeeling, road Siliguri-Kurseong [= Karsiyang] [ca. 26°52'N 088°18'E], x.1986, J. Plante leg., 1 female (MHNG);

West Bengal state: “Bengal”, without more detailed locality, date and collector’s name, AFN det., 1 spec. (FMNH);

West Bengal state: “Bengale”, without more detailed locality, date and collector’s name, ex coll. H. d’Oedekem d’Acoz, 1 female (IRSNB); “Bengale”, without more detailed locality, without date, coll. Mniszech, ex coll. R. Oberthür, 1 male (MNHN); “Bengalen”, without more detailed locality, without date and collector’s name, 1 male (SDEI);

Meghalaya state: Shilong env., Umran [ca. 25°46'N 091°52'E], 29.vi.–2.vii.1995, Werner leg., JS det., 1 female (JSCC); same locality, 29.vi.1995, without collector’s name, WB det., 6 spec. (WBAC);

Meghalaya state: W Meghalaya, Cero Hills, Nokrek Nat. Park, 25°40'N 091°04'E, 1150 m, 10.vii.1997, Afonin & Siniaev leg., 2 females (NMPC);

Karnataka state: Mysore, Shimoga distr., Agumbe Ghat [ca. 13°30'N 075°06'E], 2000 ft. [ca. 609 m], v.1974, T.R.S. Nathan leg., 1 female (MNHG);

Kerala state: Kumili, Peryiar [ca. 09°31'N 077°12'E], 23.–26.x.1997, Werner leg., JS det., 1 female (JSCC);

Kerala state: Trivandrum distr., Poonmudi Range [ca. 08°36'N 077°15'E], 3000 ft. [ca. 915 m], ix.1971, T.R.S. Nathan leg., 1 male (MNHG);

Kerala state: Travancore [historical state], Wallardi [ca. 09°35'N 077°06'E], 5.ix.1905, R. P. Favré leg., ex coll. R. Oberthür, 2 males, 1 female (MNHN);
Tamil Nadu state: Madras [= Chennai, ca. 13°05'N 080°16'E], x.1960, P.S. Nathan leg., AFN det., 1 spec. (FMNH);
Tamil Nadu state: Nilgiri Hills, Devala [ca. 11°28'N 076°23'E], 3200 ft. [ca. 975 m], v.1994, Nathan leg., 1 female (ZMAN);
Tamil Nadu state: Kotagiri env. [ca. 11°25'N 076°52'E], 27.–29.x.1997, Werner leg., JS det., 2 females (JSCC);
Tamil Nadu state: Coimba [= Coimbatore, ca. 11°00'N 076°58'E], without date and collector's name, 1 male (BMNH);
Tamil Nadu state: Anaimalai Hills, Cinchona [= Cinkona, ca. 10°17'N 076°59'E], 1067 m, xi.1959, P.S. Nathan leg., 1 male, 4 females (BPBM); Anamalai [= Anaimalai] Hills, Chinchona, 3500 ft. [ca. 1067 m], xi.1959, P.S. Nathan leg., 20 spec. (MZSP); same data, on dead cow, AFN det., 3 spec. (FMNH); Anamalai Hills, Chinchona, v.1962, P.S. Nathan leg., AFN det., 6 spec. (FMNH); same locality, without date and collector's name, ex coll. J. Negre, 3 f*f* (MNHN); Cinchona, vi.1959, dead cow, without collector's name, 1 female (AMNH); Cinchona, vii.1959, without collector's name, 1 female (AMNH);
Unprecise record: "Inde", without date, ex coll. R. Oberthür, 1 female (MNHN);
"India orientalis", without date, ex coll. Leonhardt, 1 female (SDEI);
Sri Lanka: Kandy district: Thawalamtenne, milepost 30 on Kandy – Mahiyangana road [ca. 07°20'N 080°56'E], 820 m, 18.ii.1977, K.V. Krombein, P.B. Karunaratne, P. Fernando & D.W. Balasporiya leg., black light, 1 male, 1 female (NMNH);
Kandy district: Madugoda [ca. 07°18'N 080°52'E], ca. 2600 ft. [ca. 790 m], 1.iv.1973, Baumann & Cross leg., at black light, 2 females (NMNH);
Kandy district: Candy [= Kandy, ca. 07°17'N 080°37'E], without date and collector's name, 1 female (HNHM); Kandy [ca. 07°17'N 080°37'E], 20.iii.1902, Uzel leg., 1 female (NHMW); same locality and collector, 5.iv.1902, 1 female (NHMW); same locality and collector, 15.iv.1902, 1 male (NHMW); same locality and collector, 6.v.1902, 1 male (NMPC); same locality and collector, 8.v.1902, 2 females (NHMW); same locality, 1.i.1990, Rautenstrauch leg., 1 female (SMNS);
Kandy district: Peradeniya [ca. 07°14'N 080°35'E], xii.1900, E.E. Green leg., ex coll. L. Bedel, 2 females (MNHN); same locality, 20.v.1902, Uzel leg., 1 female (NHMW); same locality, 24.i.1971, Piydasa & Somapala leg., 1 male (NMNH);
Ampara district: Ekgal Aru tank [near Ekgalara, ca. 07°11'N 081°37'E], 100 m, 19.–23.ii.1977, K.V. Krombein, P. Fernando, D.W. Balasporiya & V. Gunawardane leg., black light, 1 male, 4 females (NMNH);

Unprecise records: “Nord Ceylon”, without more precise locality, vi.1889, H. Fruhstorfer leg., 1 male, 2 females (SDEI); “Ceylon”, without more precise locality, without date, Nietner leg., 1 female (ZMHB); “Ceylan”, without more precise locality, without date, Ferrière leg., 3 females (MHNG); “Ceylon”, without more precise locality, 12.ix.1914, ex Mus. Hauschildt, 1 female (ZMUC);

Nepal: Kaski district: Mt. Annapurna, Poon Hill, 28°34'N 083°50'E, 2800 m, 12.–15.vii.1995, E. Afonin & V. Siniaev leg., JS det., 1 male (JSCC);

Rasuwa district: Ganesh Himal, ca. 20 km NE Trisuli, Mailung Khola, 28°04.5'N 085°12.5'E, 1040 m, 23.ix.1995, B. Herczig & Gy.M. László leg., 1 male, 1 female (HNHM);

Bhutan: Paro district: Gedu [ca. 26°56'N 089°30'E], 2100 m, 17.–26.vi.1988, C. Holzschuh leg., JS det., 1 female (JSCC);

China: Anhui province: Jixi env. [= Huayang, ca. 30°05'N 118°35'E], Mt. Qiyun Shan, 1200 m, vi.2005, Jin et al. leg., MN det., 4 spec. (MNIC);

Chongqing municipality: Chunking [= Chongqing, ca. 29°32'N 106°31'E], without date and collector’s name, ex coll. Em. Reitter, 1 male (NMPC);

Fujian province: Mt. Wuyishan, Xianfengling [ca. 27°49'N 117°50'E], 1200 m, 25.vii.2000, Ping-Fei Zhang & Zheng-Qing Fei leg., 1 male (IZ-CAS);

Fujian province: Mt. Liangyeshan [ca. 25°06'N 116°06'E], Yunshicai, 720 m, 9.xi.2008, Feng Yuan leg., 19 males, 20 females (IZ-CAS);

Fujian province: Nanjing County, Huboliao, Xiangxi [ca. 24°31'N 117°17'E], 24.xi.2008, Feng Yuan leg., 1 female (IZ-CAS);

Fujian province: Dehua County, Quanzhou City, Daiyushan NNR, Leifeng Station [ca. 25°42.6'N 118°13.1'E], 1.ix.2013, M.L. Jeng leg., Mercury light trap, 1 male (NMNS);

Fujian province: Dehua County, Quanzhou City, Daiyushan NNR, Houzhai Station [ca. 25°34.3'N 118°18.0'E], 2.ix.2013, M.L. Jeng leg., Mercury light trap, 1 male, 1 female (NMNS); same data, 6.ix.2013, 2 males, 1 female (NMNS); same data, 7.ix.2013, 1 male (NMNS); same data, 8.ix.2013, 1 male, 1 female (NMNS);

Fujian province: Dehua County, Quanzhou City, Nancheng, Zian Village, Zhanglingjiao [ca. 25°42.6'N 118°17.1'E], 5.ix.2013, M.L. Jeng leg., Mercury light trap, 1 female (NMNS);

Guangdong province: Dadongshan [ca. 24°56'N 112°43'E], 27.viii.1990, Z. Chen leg., 1 female (SYSU); same locality, 4.ix.1992, F. L. Jia leg., 1 female (SYSU); same locality, 4.ix.1992, W. Lin leg., 1 female (SYSU); same locality, 5.ix.1992, R. Dai leg., 1 female (SYSU); same locality, 10.ix.1993, C. Yang leg., 1 female (SYSU); same locality, 14.ix.1993, D. Yang leg., 1 female (SYSU); same locality, 5.ix.2015, without collector’s name, 1 male (SYSU);

Guangdong province: Ruyuan env., Shikankong [= Shikengkong, ca. 24°55'N 113°00'E], 1700 m,

ix.2003, Ying leg., MN det., 4 spec. (MNIC);
Guangdong province: Longmen county [ca. 23°44'N 114°15'E], 23.vii.2011, without collector's name, 2 males (SYSU);
Guangxi autonomous region: Fangchenggang City, Fulong Township [ca. 21°48'N 107°57'E], 240 m, 15.iii.1998, Ge-Xia Qiao leg., light trap, 1 female (IZ-CAS);
Guangxi autonomous region: Fangchenggang City, Banba Township [ca. 21°40'N 107°39'E], 500 m, 4.vi.2000, Yan-Zhou Zhang, Jian Yao & Wen-Zhu Li leg., 5 males, 3 females (IZ-CAS);
Guangxi autonomous region: Napo County, Defu [ca. 23°19'N 105°48'E], 1350 m, 19.vi.2000, Chao-Dong Zhu leg., 1 female (IZ-CAS); same locality, 1300 m, 14.–15.viii.2000, Wen-Zhu Li & Fu-Sheng Huang leg., 2 males (IZ-CAS);
Guangxi autonomous region: Rongshui [= Miao Autonomous County], Wangdong Township, Pingshi Village [ca. 25°15'N 108°41'E], 450–900 m, 4.–5.viii.2003, Xiu-Juan Yang leg. 1 male (IZ-CAS);
Hainan province: Tongshen City, Maoyang Town [ca. 18°56'N 109°30'E], 28–31.x.1992, Zhu-Yao Liu, Tian-Qi Wang & Hai-Sheng Yin leg., 33 spec. (SEMC);
Hainan province: Limu Shan Mts., 20.v.1984, without collector's name, 1 male, 1 female (SYSU); same data, 24.v.1984, 2 males (SYSU); same data, 25.v.1984, 1 male (SYSU); same data, 26.v.1984, 4 females (SYSU); Limushan Township, Limushan Nature Reserve, 19°10'25"N 109°43'46"E, 652 m, without date, J. C. Liu leg., Yun Ji det., 2 females (JCLC);
Hainan province: Hainan Island, Mt. Wuzhi Shan, 1500 m, 18°53'N 109°43'E, 20.ii.–10.iv.2001, without collector's name, MN det., 5 spec. (MNIC); same data, 1 male (JRUC); same locality, 18°54'N 109°40'E, 730 m, 7.xii.2007, Jian Yao leg., 3 females (IZ-CAS); same locality, gate, 18.90°N 109.67°E, 708 m, 8.iv.2010, Mei-Ying Lin leg., light trap, 1 male (IZ-CAS); same locality, 9.iv.2010, Mei-Ying Lin leg., light trap, 1 male (IZ-CAS);
Hainan province: Ledong County, Jianfengling, Summer resort, 18°45'N 108°50'E, 930 m, 17.xii.2007, Jian Yao leg., light trap, 1 male, 1 female (IZ-CAS);
Hainan province: Ledong County, Jianfengling, Tianchi summer resort, 18.74363°N 108.84311°E, 982 m, 25.–26.xi.2008, Hong-Liang Shi leg., light trap, 1 female (IZ-CAS);
Hainan province: Ledong County, Jianfengling, Mingfenggu, 18.74357°N 108.84288°E, 983 m, 6.xii.2009, Xin-Lei Huang leg., 1 female (IZ-CAS);
Hainan province: Hainan Island, Ledong county, Jianfengzhen, Mt. Jianfengling [ca. 18°43'N 108°53'E], 19.iv.–21.vi.2014, Liu Bin leg., light trap, 2 females (JRUC);
Hainan province: Baisha County, Hongxin Village [ca. 19°04'N 109°33'E], 500 m, 16.xi.2008, Zong-Yi Zhao leg., mountain forest, light trap, 1 male (IZ-CAS);
Hainan province: Baisha County, Nankai Township, Yinggeling, 19.08001°N 109.41058°E, 259 m,

19.xi.2008, Hong-Liang Shi leg., light trap, 1 female (IZ-CAS);

Hainan province: Baisha County, Hongkan Reservoir, 19.08188°N 109.49985°E, 553 m, 4.v.2009, Xin-Lei Huang leg., 1 male, 1 female (IZ-CAS);

Hainan province: Baisha County, Nankai Township, Nanmaola, 18.95355°N 109.38460°E, 1261 m, 14.v.2009, Xin-Lei Huang leg., 1 female (IZ-CAS);

Hainan province: Qiongzhong County, Yinggen Town, Baihualing, 19.00°N 109.81°E, 420 m, 26.xi.2009, Xin-Lei Huang leg., 1 male (IZ-CAS);

Hainan province: Lingshui County, Diaoluoshan Mt., 18°43'N 109°51'E, 1000 m, 24.iv.2012, Peng & Dai leg., 1 male (SNUC); Lingshui County, Diaoluoshan Mt. Reservoir, 18°43'N 109°53'E, 600–1000 m, 26.iv.2012, Peng & Dai leg., 1 male (SNUC);

Hunan province: Xinhua env., Mt. Tianlong Shan [ca. 27°42'N 111°07'E], 1500 m, vii.2005, Yi et al. leg., MN det., 7 spec. (MNIC);

Jiangxi province: Wuyi Shan Mts., Xipaihe vill., 27°54'N 117°20'E, 1500 m, vii.2003, team of V. Siniaev leg., 1 male, 2 females (JSCC);

Shaanxi province: Daba Shan Mts., 1–15 km S Shou-Man, 32°14'N 108°34'E, 1000–1600 m, vi.–vii.2003, V. Siniaev et al. leg., 1 male (JRUC);

Xizang autonomous region: Motuo, 80K [= Medog, ca. 29°19'N 095°20'E], 2100 m, 20.–23.viii.2011, Wei Hu leg., 1 male (SNUC);

Xizang autonomous region: Linzhi Pref., Metog [= Motuo] County, Dêxing [= Dexing] Township [ca. 29°20'N 095°18'E], 25.viii.2013, X.D. Yang leg., Yun Ji det., 1 male (YJIC);

Xizang autonomous region: same locality, Baibung [= Beibeng] Township [ca. 29°14'N 095°10'E], 27.vii.2014, J.Y. Wang leg., Yun Ji det., 3 females (CAU); Motuo County, Beibengxiang, 29.2431°N 095.1700°E, 799 m, 19.viii.2015, Hong-Bin Liang leg., 2 males, 1 female (IZ-CAS); same data, 20.viii.2015, 1 male, 1 female ((IZ-CAS);

Xizang autonomous region: Motuo County, Ximohe bridge, 29.3519°N 095.3417°E, 707 m, 17.viii.2015, Hong-Bin Liang leg., 2 males (IZ-CAS);

Xizang autonomous region: Motuo County, Lagongchachung, 29.31879°N 095.31570°E, 1250 m, 17.viii.2015, Hong-Bin Liang leg., 2 males, 3 females (IZ-CAS);

Yunnan province: Jinghong, Nabanhe Reserve, Benglong Village env., 22.16°N 100.65°E, 858 m, 2.x.2010, Mei-Ying Lin leg., 2 males, 2 females (IZ-CAS);

Yunnan province: Mengla County, Menglun Town [ca. 21°56'N 101°14'E], 800 m, 10.iv.1981, Fa-Xian Li leg., 1 male, 1 female (CAU);

Yunnan province: Yuanyang County [ca. 23°13'N 102°50'E], 1630 m, 5.v. 1982, Gen-Tao Jin leg., 1 female (SEMC);

Yunnan province: Mengla County, Xishuangbanna State, 1.5 km E Shangyong [ca. 21°14'N

101°44'E], 6.x.2013, M.L. Jeng leg., light trap, 1 male, 1 female (NMNS);
Yunnan province: Nabanhe Nat. Reserve, Guomenshan [ca. 22°18'N 100°39'E], 1100 m, 23.vii.2005, Li & Li leg., 1 male (SNUC); same locality, 20.xi.2008, J.-Y. Hu & L. Tang leg., 1 male (SNUC);
Yunnan province: Menghai County, Nabanhe Reserve, Guomenshan Station, 22.24°N 100.60°E, 1120 m, 4.–5.x.2010, Mei-Ying Lin leg., light trap, 12 males, 17 females (IZ-CAS);
Yunnan province: Jinping County, Fenshuiling [ca. 24°14'N 103°03'E], 1870 m, 27.v.2009, Liu, Wu, Zhu & Bi leg., light trap, 1 female (SEMC);
Yunnan province: Menghai County, Nabanhe Reserve, Guomenshan, Hongdoushanyakou, 22.24°N 100.62°E, 1435 m, 5.x.2010, Mei-Ying Lin leg., 7 males, 2 females (IZ-CAS);
Yunnan province: Mengla County, Xishuangbanna Nature Reserve [ca. 21°53'N 101°18'E], viii.2012, Guo-Feng Li leg., 1 male (YFTC);
Yunnan province: Puer City, Laiyanghe National Forest Park [ca. 22°36'N 101°00'E], viii.2013, Guo-Feng Li leg., light trap, 2 males, 1 female (YFTC);
Yunnan province: Xishuangbanna, Bubang Village [ca. 21°35'N 101°35'E], 700 m, 15.ix.1993, Long-Long Yang leg., light trap, 1 female (IZ-CAS); same locality, 9.–10.x.2017, carrion of *Herpestes urva*, without collector’s name, 1 female (IZ-CAS);
Zhejiang province: Lin’an env., Mt. [West] Tianmu Shan [ca. 30°21'N 119°25'E], 1300 m, viii.2005, Li et al. leg., MN det., 1 spec. (MNIC).
Taiwan: Nantou county: Lugu Township, Fenghuang valley (鳳凰谷), 23.73059°N 120.79989°E, 3.ix.2020, Bin-Hong Ho, K.W. Chan & Y. Ho leg., light trap, 1 female (BHHC);
Hualien county: Guangfu Township, Manuo [瑪娜], 23.6491°N 121.4773°E, 19.viii.2020; Bin-Hong Ho et al. leg., light trap, 1 male (FSHC);
Nantou county: Zhushan Township, Yingfengbao (迎風堡), 23.6449°N 120.7820°E, 4.ix.2021, Zhen-Yi Chen et al. leg., light trap, 1 male (ZYCC);
Pingtung county: Neipu Township, Laopi Village (老埤村), 22.6426°N 120.6097°E, 11.vii.2016, Cheng-Yan Tu leg. & det., 1 spec. (CYTC);
Taitung county: Taimali Township, Jinlun (金崙) [ca. 22.533°N 120.963°E], 08.ix.2021, W.C. Liao leg., light trap, 1 spec. (FSHC);
Japan: Okinawa prefecture: Nansei-shoto [= Ryukyu] Islands: Yaeyama-retto Islands: Ishigaki Island, Banna-dake Mt. [ca. 24°22.3'N 124°09.8'E], 22.v.1973, K. Akiyama leg., 1 female (NSMT) [attracted to a mercury lamp of the TV relay station at the summit of Mt. Banna-dake (Kurosawa 1974)];
Myanmar: Kachin state: 40 km N Myitkyina, Chanc Kand [ca. 25°44'N 097°23'E], 235 m, 23.–

24.iv.1998, S. Murzin & V. Siniaev leg., JS det., 1 male, 1 female (JSCC);
Mandalay region: Mt. Popa [ca. 20°55'N 095°15'E], 30.viii.1976, N. Yoshida leg., 1 female (NSMT);
Kayin state: Dawna [= Dawna Range, ca. 16°41'N 098°20'E], ix.1990, without collector's name, 1 female (SMNS);
Dalona [not located], ix.1990, ex coll. K.-G. Bernhardt, 1 male (NMNH);
Unprecise record: "SE Burma" without more precise locality, 20.viii.1990, without collector's name, 1 male (NHMW);
Thailand: Chiang Mai province: Chiang Mai env., Mae Ai [ca. 20°02'N 099°18'E], vii.1990, N. Koyana leg., MN det., 1 spec. (MNIC);
Chiang Mai province: Doi Chiang Dao [ca. 19°24'N 098°52'E], 1150 m, 2.iv.1986, P. Schwendinger leg., on light, 1 female (MHNG); 100 km N Chiang Mai, Chiang Dao Hill Reserve, 600 m, 1.–7.iii.2010, S. Murzin leg., 2 females (JRUC); same locality, 10.–23.iii.2010, S. Murzin leg., 2 males, 2 females (JRUC);
Chiang Mai province: Huai Nam Dang Nat. Park [ca. 19°16'N 098°36'E], 1500–1700 m, 25.–27.x.1984, Karsholt, Lomholdt & Nielsen leg., 1 female (ZMUC);
Chiang Mai province: Doi Pui [= Doi Pui Nat. Park, ca. 18°49'N 098°53'E], 29.vi.1984, A.M. Cotton leg., 2 females (NSMT); same locality, 20., 29.vii.1984 & 3.viii.1984, without collector's name, MN det., 5 spec. (MNIC); same locality, 13.ix.1987, without collector's name, 3 spec. (SMNS);
Chiang Mai province: Chiang Mai [ca. 18°47'N 099°00'E], viii.1996, W.T. Fatt leg., 1 male, 1 female (HNHM);
Chiang Rai province: Doi Mae Salong [ca. 20°18'N 099°32'E], 27.–28.vii.1999, K. Masumoto leg., MN det., 2 spec. (MNIC);
Chiang Rai province: Yanae, 28 km W Mae Suai [ca. 19°39'N 099°17'E], 24.xi.2003, UV light, L. Pergovits, M. Foldvári, Á. Kőrösi, A. Szappanos & B. Maklári-Kis leg., 2 females (HNHM);
Chon Buri province: Sriracha env. [= Si Racha, ca. 13°09'N 100°56'E], Nong Khor, 23.ix.1925, H.M. Smith leg., 1 male, 2 females (NMNH);
Nakhon Nayok province: Kao Yai Nat. Park [ca. 14°20'N 101°30'E], 16.xi.1988, M. Jäch & Kukol leg., 1 male (NHMW);
Nan province: Doi Phu Kha Nat. Park [ca. 19°10'N 100°56'E], 34.4 km from Ha, 1685 m, 1992, I. Kitching leg., 1 female (BMNH);
Nan province: Pua [= Ban Pua, ca. 18°46'N 100°48'E], 1700 m, 16.vii.2001, P. Henschel & G. Petráni leg., 2 males, 1 female (HNHM);
Narathiwat province: Narathiwat, km 17 on Ban Tabing Tingngi to Sri Sakhon Rd. [ca. 06°25'N

101°49'E], 250 m, 1.xii.1991, I. Kitching leg., 1 female (BMNH);
Phetchabun province: 22 km Lom Sak to Khon Kaen [ca. 16°45'N 101°13'E], Hwy 12, 620 m, xi.1991, I. Kitching leg., 1 female (BMNH);
Ranong province: Ranong [ca. 09°58'N 098°38'E], 20.vii.1990, without collector’s name, 2 males, 5 females (NHMW); same data, WB det., 2 spec. (WBAC);
Ranong province: TV relay Stn. 7 km N of Ranong, 300–500 m, 28.–29.xi.1991, I. Kitching leg., 2 males, 4 females (BMNH);
Rayong province: 1 mi E Kilosip [ca. 12°41'N 100°57'E], 10.xi.1972, without collector’s name, DSS det., 1 male (UCDC);
Saraburi province: Saraburi [ca. 14°31'N 100°55'E], iv.1988, Steinke leg., 1 male, 2 females (JRUC); same data, 1 male (JVAC); same data, 1 male, 1 female (NHMW); same locality, iv.1989, without collector’s name, WB det., 48 spec. (WBAC);
Yala province: Khao Pok Yo [ca. 06°21'N 101°21'E], 340 m, 1 km from summit, 6.xii.1991, I. Kitching leg., 1 male (BMNH);
Chiang Mai province: 15 km Samoeng to Hang Dong Rd. [ca. 18°48'N 098°50'E], 460 m, 3.i.1992, I. Kitching leg., 1 male (BMNH); Samoeng, vi.1988, without collector’s name, 1 male (NSMT);
Laos: Louang Namtha province: 20 km NW Louang Namtha, 21°09.2N 101°18.7E, 900 m (± 100 m), 5.–11.v.1997, E. Jendek & O. Šauša leg., JS det., 1 female (JSCC);
Louang Namtha province: 5 km SW Muang Sing, Chiang Tung Stupa [ca. 21°10'N 101°08'E], 750 m, 26.iii.–5.iv.2010, S. Murzin leg., 3 females (JRUC);
Louang Namtha province: 65 km NW Luang Nam Tha, Nam Tha National Protected Area [= Nam Ha, ca. 20°49'N 101°15'E], 1050 m, 8.–15.iv.2010, S. Murzin leg., 2 males, 6 females (JRUC);
Louang Phrabang province: 5 km W of Ban Song Cha, 20°33–34'N 102°14'E, ca. 1200 m, 24.iv.–16.v.1999, C. Holzschuh leg., JS det., 2 males, 9 females (JSCC); same locality and date, V. Kubáň leg., JS det., 1 male, 1 female (JSCC); same locality, 1.–16.v.1999, V. Kubáň leg., 4 males, 7 females (NHMB); same data, 1 male, 1 female (JRUC);
Louang Phrabang province: 240 km N Vientiane, 10 km N Louang-Phrabang [ca. 19°58'N 102°08'E], near Mekong, hills ca. 250 m, vi.1993, I. Somsy leg., at light, 1 male, 3 females (ZMHB);
Houa Phan province: Ban Kangpabang env., 25 km SE of Vieng Xai, 20°19'N 104°25'E, 14.–18.v.2001, by road, D. Hauck leg., JS det., 1 female (JSCC);
Houa Phan province: Mt. Phu Pane, 20°12'N 103°59'E, 900–1600 m, 10.–21.vi.2010, S. Jakl and Lao collectors leg., 2 males, 2 females (JRUC);
Xiang Khoang province: Xieng Khouang [= Xiang Khoang, ca. 19°19'N 103°22'E], vi.1996 & vi.1997, without collector’s name, MN det., 3 spec. (MNIC);

Sainyabuli province: Puu Soai [ca. 17°44'N 101°00'E], 9.viii.1996, without collector's name, JS det., 2 females (JSCC);
Sekong province: 51 km N Sekong, Ho Chi Minh trail, 15°48.1'N 106°39.4'E, ca. 580 m, 13.–15.v.2010, on light, S. Jakl leg., 2 males, 3 females (JRUC); same data, J. Hájek leg., 1 male (NMPC);
Attapu province: Bolaven Plateau, 15 km SE of Ban Houaykong, Nong Lom (lake) env., 15°02'N 106°35'E, 800 m, 18.–30.iv.1999, E. Jendek & O. Šauša leg., JH det., 1 male (JHAC);
Vientiane prefecture: Ban Van Eue [= Ban Van Heue, ca. 18°19'N 102°01'E], 30.ii.1967, native collector, 1 female (BPBM); Ban Van Heue, 20.xi.1968, [J.] Rondon leg., 1 female (BPBM);
Vientiane prefecture: Vientiane [ca. 17°58'N 102°36'E], x.1959, R. Bandon leg., 1 male, 1 female (MNHN);
Unprecise and unlocated records: "Annam highlands", without date and collector's name, 1 male (HNHM);
"Ban Kum" [not located], 20.xi.1968, J. Rondon leg., 3 males, 3 females (BPBM);
Cambodia: Kirirom Nat. Park, 11°21'23"N 104°04'38"E, 780 m, 2.–17.i.2000, M. Murzin & S. Murzin leg., JS det., 1 female (JSCC);
Vietnam: Cao Bang province: Cao Bang [ca. 22°41'N 106°17'E], 3.vii.1997, native collector's leg., KH det., 2 males (KHAC); Mt. Pia Oac [ca. 22°37'N 105°52'E], 1200 m, 25.v.1999, S. Nomura leg., light trap, 1 male, 1 female (NSMT);
Lao Cai province: Sapa [= Sa Pa, ca. 22°20'N 103°50'E], v.1985, without collector's name, MN det., 2 spec. (MNIC); same locality, 19.vi.1990, without collector's name, WB det., 4 spec. (WBAC); same locality, v.1991, J. Krček leg., JS det., 6 males, 7 females (JSCC); same locality, 1530 m, 25.v.–9.vi.1991, J. Strnad leg., WB det., 1 spec. (WBAC); same locality, 22°20N 103°30E, 25.v.–10.vi.1991, E. Jendek leg., 5 males, 16 females (NHMW); same data, WB det., 4 spec. (WBAC); same locality, 1500 m, 18.vi.2002, S. Nomura leg., light trap, 1 male (NSMT); same locality, 1500 m, 21.vi.2002, S. Nomura leg., light trap, 1 male (NSMT); same locality, 1500 m, 18.v.2003, S. Nomura leg., light trap, 1 female (NSMT);
Lao Cai province: Hoang Liang Nature Park, Tram Ton, 22.3494°N 103.7704°E, 1915 m, 8.–11.iv.2010, L. Papp, L. Peregovits & Z. Soltész leg., forest edge, at light, 1 female (HNHM);
Lao Cai province: trickle along trail Sapa – Muong Hoa [= Mong Hoa, ca. 22°19'N 103°50'E], Ho river rice paddies, 11.v.1995, D. Currie & J. Susann leg., UV light, SBP det., 1 spec. (ROM);
Lao Cai province: Sa Pa district, Cat-Cat village, 22°19'36.4″N 103°49'46.1″E, 1250 m, 4.–9.viii.1998, A. Kun leg., at light, 1 female (HNHM);
Lao Cai province: Fan-Si-Pan [= Phan Si Phang, ca. 22°18'N 103°46'E], vii.1994, V. Siniaev leg., JH det., 2 males, 3 females (JHAC); same locality, 7.v.1997, native collector's leg., KH det., 1

female (KHAC); same locality, vi.1997, native collector's leg., KH det., 1 male (KHAC); same locality, ix.1997, native collector's leg., KH det., 1 female (KHAC); Deo Tram Ton pass near Sa Pa, 27.vi.1997, S. Nomura leg., light trap, 2 females (NSMT); same locality, 3.vi.1999, S. Nomura leg., light trap, 1 female (NSMT);

Son La province: Deo Pha Din [ca. 21°34'N 103°32'E], 24.vi.1997, S. Nomura leg., light trap, 2 female (NSMT);

Vinh Phu province: Tam Dao mountain range [ca. 21°27'N 105°38'E], 900 m, 26.v.1985, L. Medvedev leg., 1 male (SMNS); same locality, 26.v.–3.vi.1986, J. Strnad leg., 2 females (JRUC); same locality, 20.–28.iv.1990, J. Strnad leg., WB det., 1 spec. (WBAC); same locality, v.1990, J. Picka leg., JH det., 2 males, 4 females (JHAC); same locality, 6.–20.v.1990, M. Kalabza & Z. Košťál leg., 1 female (JRUC); same locality, 8.–22.v.1990, M. Dudycha leg., 1 female (JRUC); same locality, v.–vi.1990, R. Plch leg., 1 female (JRUC); same locality, 20.vi.1990, M. Dvořák leg., 2 females (JRUC); same locality, 23.vi.1990, Makovsky leg., WB det., 1 spec. (WBAC); same locality, 900 m, 6.–10.ix.1990, J. Horák leg., 1 male (JRUC); same locality, xi.1990, G. Csorba leg., 1 female (HNHM); same locality, 900 m, 16.v.1991, J. Strnad leg., JS det., 3 males, 16 females (JSCC); same locality, 15.v.–16.vi.1991, 1 female (NHMW); same locality, xii.1991, without collector's name, 1 female (JRUC); same locality, 10.–25.vi.1992, V. Seidl leg., 1 male (NMPC); same locality, 23.–31.v.1993, without collector's name, MN det., 1 spec. (MNIC); same locality, 930 m, 23.–26.ix.1994, S. Ueno leg., 3 males (NSMT); same locality, station in primary forest, 21°27'N 105°39'E, 930 m, 11.–14.x.1994, D.C. Currie leg., SBP det., 1 spec. (ROM); same locality, v.1995, native collector's leg., KH, 1 male (KHAC); same locality, vii.1996, A. Monastyrskii leg., AFN det., 7 spec. (FMNH); same locality, vi.1997, native collector leg., 1 male (NSMT); same locality, 14.–18.v.1999, S. Nomura leg., light trap, 1 male, 2 females (NSMT);

Vinh Phu province: Me Linh District, Ngoc Thanh commune, Me Linh Station for Biodiversity, 21.3843°N 105.7122°E, 80 m, 1.–4.x.2016, O. Merkl & Phu Pham Van leg., secondary forest, at light, 1 female (HNHM);

Hoa Binh province: "Reg. de Hoa Binh" [ca. 20°50'N 105°19'E], 1928, A. de Cooman leg., 1 male, 1 female (MNHN);

Tuyen Quang province: Tonkin centr. [historical region], Tuyen-Quan env. [= Tuyen Quang, ca. 21°49'N 105°12'E], vii.–ix.1901, A. Weiss leg., ex coll. A. Grouvelle, 1 male (NMNH);

Ninh Binh province: Cuc Phuong [National Park, ca. 20°19'N 105°37'E], 11.–13.x.1995. S. Nomura leg., light trap, 1 male (NSMT); same locality, 7.iii.1999, A. Shinohara leg., 2 females (NSMT);

Nghe An province: Con Cuong, 18°56'N 104°49'E, 31.x.1994, D.C. Currie leg., edge of town, UV light, SBP det., 2 spec. (ROM); Po Phuong, 19°36.442'N 104°58.302'E, 280 m, 17.–26.x.1999, F. Kassai leg., 2 males, 1 female (HNHM);

Thua Thien-Hue province: Alona forest, Ho Chi Minh Road [= Highway] [not located], 600–800 m, 11.vi.2009, A. Luoi leg., 7 males, 4 females (JRUC);

Thua Thien-Hue province: Bach Ma Mt. [ca. 16°13'N 107°51'E], 1400 m, iv.2016, Thanh Le & team leg., 2 males, 1 female (LDVC); same data, 1 female (JRUC);

Gia Lai province: An Khe [ca. 13°57'N 108°40'E], without date, G.B. Marshall leg., DSS det., 1 male (DSSC);

Gia Lai province: Pleiku, Nui Hon Cong, 13°59'N 108°38'E, 700 m, v.1967, G.W. Ulrich leg., light trap, 1 male CAS);

Lam Dong province: 12 km N of Dalat [= Da Lat], Lang Bian, 12°03'N 108°27'E, 1580–1750 m, 17.–21.iv.1995, Dembický & Pacholátko leg., 1 female (JRUC); Da Lat [ca. 11°55'N 108°27'E], 14.x.1988, S. Mahunka & T. Vásárhelyi leg., 1 male, 1 female (HNHM); same data and collectors, 15.x.1988, 3 females (HNHM); same data and collectors, 17.x.1988, 2 males, 3 females (HNHM); same data and collectors, 18.x.1988, 1 male (HNHM); same data and collectors, 19.x.1988, 1 female (HNHM); same data and collectors, 14.–20.x.1988, 3 females (HNHM); Annam highlands, Agric. Station of Blao [= Bao Loc, ca. 11°31'N 107°47'E], 800 m, 9.iv.1933, M. Poilane leg., 1 female (NMNH); Bao Lac, Dei Binh stream [= Da Binh stream, ca. 11°30'N 107°51'E], 12.x.1988, S. Mahunka & T. Vásárhelyi leg., 1 female (HNHM);

Dong Nai province: 30 miles NE Saigon, Trang Bom [ca. 10°57'N 107°00'E], 7.ii.1932, M. Poiane leg., 4 males, 6 females (NMNH);

Unprecise records: "Tonkin" [historical region, ca. 22°00'N 105°00'E], without date, ex coll. R. Oberthür, 1 female (MNHN); same locality, 1903–1906, P. Lamée leg., ex coll. R. Oberthür, 1 female (MNHN);

Philippines: Luzon Island, Kalinga-Apayao province, Cordillera centr. mts, Saltan Upper valley, 17°30'N 121°08'E, 750 m, 22.iii.2000, L. Dembický leg., 2 males, 4 females (JSCC);

Luzon Island, Mountain province, Sagada [ca. 17°05'N 120°53'E], 1550 m, 21.–23.vii.1985, M. Owada leg., 1 male, 1 female (NSMT);

Luzon Island, Imugan [ca. 16°12'N 120°52'E], N. Vicaya, without date, Baker leg., 1 female (NMNH);

Luzon Island, Manila [ca. 14°35'N 120°59'E], without date and collector's name, 1 male (ZMHB); same locality, without date [but before 1942], W. Schultze leg., 2 females (SMTD);

Leyte Island, Leyte, Mahapag, Brgy: Tagpili, 600 m, 10.8°N, 124.8°E, 15.vi.2004, D. Mohagan leg., DSS det., 2 spec. (DNA-voucher), 2 males, 5 females (DSSC); same data, 1 male (MZLU);

Leyte Island, 11.iii.1984, T. Borromeo leg., MN det., 2 spec. (MNIC);

S Leyte Island, iv.2002, without collector's name, MN det., 4 spec. (MNIC);

Mindanao Island, Bukidnon [province], Santa Fe [ca. 08°21'N 124°47'E], without date, L.H.

Phillips leg., AFN det., 2 spec. (FMNH); same locality, 2000 ft [ca. 610 m], 22.v.1932, F.C. Hadden leg., 4 males, 4 females (CAS); same data, 1.vi.1932, 1 male (CAS); Mindanao Island, Bukidesa [= Bukidnon province], Santa Fe [ca. 08°21'N, 124°47'E], 619 m, 5.x.1933, without collector's name, DSS det., 1 male (UCDC);

Mindanao Island, Bukidnon [province], Silipon [= Sil-Ipon, ca. 08°14'N 124°49'E], 10.vi.1932, L. Phillips leg., ex coll. Van Dyke, 1 male (CAS);

Mindanao Island, 30 km E Malaybalay, Busdi [ca. 08°16'N 125°15'E], 1000 m, 5.–9.v.1996, Bolm leg., 1 female (SMNS);

Mindanao Island, Mt. Apo [ca. 07°01'N 125°16'E], v.2006, without collector's name, 1 spec. (MNIC);

Mindanao Island, Parang [ca. 07°20'N, 124°14'E], vi.1945, J.J. Marquis leg., 1 male (CAS);

Mindanao Island, Malapang [ca. 07°09'N, 124°40'E], v.1945, J.J. Marquis leg., 1 male (CAS);

Mindanao Island, Davao City, Marilog District, Malambo Range, Busay Resort, 07°29.0′N 125°15.7′E, 1200 m, 23.–27.ii.2017, P. Šípek & D. Vondráček leg., montane forest, UV+white light traps, 9 males, 11 females (JRUC);

Malaysia: <u>Kedah state</u>: without more precise locality, 1986, without collector's name, MN det., 4 spec. (MNIC);

<u>Kedah state</u>: Talan Sintok [ca. 06°26'N, 100°31'E], iv.–vii.1938, A.J. Slater leg., ex mass of trop. vegetation, 3 males, 5 females (BMNH);

<u>Perak state</u>: Bukit Larut, Maxwell hill, 04°51.72'N 100°47.99'E, 1100 m, 19.vii.2000, M. Niehuis & O. Niehuis leg., 1 male (SMNS);

<u>Perak state</u>: Trong [= Terung, ca. 04°42'N 100°42'E], without date, W.L. Abbott leg., 1 male (NMNH);

<u>Perak state</u>: Cameron Highlands, i.1985, Wong leg., 1 male (SMNS);

<u>Perak/Pahang state</u>: Cameron Highlands, viii.1982, T. Lander leg., 1 male, 1 female (MHNG); same locality, 1986, without collector's name, MN det., 7 spec. (MNIC); same locality, vi.1986, without collector's name, 1 female (NSMT); same locality, v.2002, without collector's name, MN det., 5 spec. (MNC);

<u>Pahang state</u>: Cameron Highlands, without date and collector's name, WB det., 1 spec. (WBAC);

<u>Pahang state</u>: Cameron Highlands, Habu [ca. 04°26'N 101°23'E], 1190 m, 7.xii.2014, without collector's name, 2 males, 3 females (JRUC);

<u>Pahang state</u>: Cameron Highlands, Tanah Rata [ca. 04°28'N 101°23'E], 5000 ft. [ca. 1525 m], 20.–21.xii.1967, E.W. Classey leg., 2 males (BMNH); same locality, 7.iv.1976, M. Hata leg., KH det., 1 male (KHAC); same locality, iii.1977, T. Jaccoud leg., 2 females (MHNG); same locality, 4700 ft. [ca. 1430 m], 3.x.–13.xi.1980, without collector's name, ex coll. A. Harman, 1 male, 1 female

(BMNH); same locality, 23.vi.1982, without collector's name, MN det., 2 spec. (MNIC); same locality, 14.–18.iii.1988, Tams Expedition, G. Hangay & T. Ralph leg., 1 female (HNHM); same locality, 1330 m, 1989, ex coll. E.S. Ross, 2 males (CAS); same locality, 25.i.1991, A. Elferink leg., ex coll. J. Roggeman, 1 male, 1 female (IRSNB); same locality, 21.iii.–2.iv.1995, O. Merkl leg., edge of degradated rainforest, at light, 1 female (HNHM); same locality, 7.viii.1997, M. Kon leg., DSS det., 1 male (DNA-voucher) (DSSC);

Pahang state: Cameron Highlands, 2 km S Tanah Rata, on Tapah road [ca. 04°27'N 101°23'E], 29.iii.1995, O. Merkl & I. Szikossy, montane rainforest, at light, 5 males, 6 females (HNHM);

Pahang state: Fraser's Hill [= Bukit Fraser, ca. 03°43'N 101°44'E], 1300 m, 16.iii.1966, light trap, J. Sedlacek leg., 1 male (BPBM); same locality, 1300 m, 16.iii.1966, mercury vapor light trap, J. & M. Sedlacek leg., 1 male, 4 females (BPBM); same locality, gap road, 1190 m, 8.–10.viii.1986, G.S. Robinson leg., 1 female (BMNH); 110 km N Kuala Lumpur, Fraser's Hill [= Bukit Fraser, ca. 03°43'N 101°44'E], 1500 m, 7.–10.i.1995, S. Bečvář & J. Bečvář leg., JH det., 1 male, 1 female (JHAC); same data, 3 females (JRUC); same locality, 10.v.1996, S. Snäll leg., JS det., 3 females (JSCC); same locality, 25.iii.2012, B. Makovský leg., 1 male (JRUC);

Pahang state: Genting Highlands [ca. 03°24'N 101°46'E], iv.1991, Chlopčík leg., 1 female (NMPC);

Malacca state: Malacca [= Melaka, ca. 02°12'N 102°15'E], without date, ex coll. M. Pic, 1 female (MNHN);

Johor state: Endau Rompin Nat. Park [ca. 02°30'N 103°20'E], Salendang, 100 m, 28.ii.–12.iii.1995, Štrba & Hergovits leg., 1 male, 1 female (NHMW);

Sarawak state: Niabi Pare [= Niah National Park, ca. 03°50'N 113°43'E], 19.viii.1972, without collector's name, MN det., 3 spec. (MNIC);

Sarawak state: Kapit [ca. 02°00'N 112°56'E], 30.vii.1986, K. Maruyama leg., MN det., 1 spec. (MNIC);

Sarawak state: Semongok [= Semengok, ca. 01°24'N 110°20'E], 4.viii.1967, G.H.L. Rothschild leg., light trap, 2 females (BMNH); same locality and collector, xi.1967, 1 female (BMNH); same locality, 29.xi.1967, S. Juses leg, light trap, 1 male (BMNH);

Sarawak state: Bau district, Bidi [ca. 01°23'N 110°08'E], 90–240 m, 2.ix.1958, T.C. Maa leg., 1 female (BPBM);

Sarawak state: Taber Longhouse [?= Tabau, ca. 01°04'N 111°50'E], 5.–12.viii.1977, without collector's name, MN det., 10 spec. (MNIC);

Sarawak state: [without detailed locality], vi.–ix.1958, T.C. Maa leg., 1 female (BPBM);

Sabah state: Kundasang [= Kundassang, ca. 05°59'N 116°34'E], 1000 m, 5.v.1984, M. Nishikawa leg., at light, MN det., 2 spec. (MNIC);

Sabah state: near Ranau [ca. 05°58'N 116°41'E], ca. 300 m, 1.–2.viii.1985, M. Nishikawa leg., at

light, MN det., 3 spec. (MNIC);

Sabah state: 50 km E Kota Kinabalu, Crocker mts, Gunung Emas [ca. 05°55'N 116°27'E], 16.–27.iv.1993, Štrba & Jeniš leg., 1 male (NHMW); Crocker Mt., Bunong Emas [= Gunung Emas], 500–1900 m, 6.–21.v.1995, J. Stolarczyk leg., JH det., 1 male (JHAC); same locality and date, I. Jeniš leg., JS det., 1 female (JSCC);

Sabah state: Sandakan [env.], Sepilok [ca. 05°52'N 117°57'E], 8.ix.1982, S. Nagai leg., 1 female (NSMT); same locality, 27.v.1987, S. Nagai leg., 1 male (NSMT);

Sabah state: SW slope of Mt. Trus Madi [ca. 05°35'N 116°29'E], 1200 m, 29.iii.1993, M. Sawai leg., at light, KH det., 2 females (KHAC); Mt. Trus Madi, 1200 m, 8.–20.iv.2002, without collector’s name, MN det., 1 spec. (MNIC); same locality, iv.2003, without collector’s name, MN det., 1 spec. (MNIC); same locality, 6.–19.iv.2004, without collector’s name, MN det., 2 spec. (MNIC);

Sabah state: Bunsit [ca. 05°26'N 116°10'E], Bandukan area, 3.–4.ix.1995, C. Adrian leg., pitfall traps, rotten organic matter, KH det., 1 male (KHAC);

Sabah state: Gn. Alab [ca. 05°20'N 116°32'E], 29.iv.1988, K. Maruyama leg., MN det., 2 spec. (MNIC);

Sabah state: Keningau area, Tenom Agic. Res. Station [ca. 05°07'N 115°56'E], 230 m, 23.xi.1987, garden, at light, Krikken & Rombaut leg. (sa43), 1 male (RMNH);

Sabah state: Keningau [env.], Bunsit [ca. 05°26'N 116°09'E], iii.–v.1984, M. Itoh leg., 1 male, 1 female (NSMT);

Brunei: Lamunin, Bukit Sulang, 04°38'N 114°34'E, 6.viii.–12.ix.1982, N.E. Stork leg., 1 female (BMNH);

Indonesia: Aceh province: 30 km W Peureulak, Krueng toan [ca. 04°49'N 097°37'E], 80 m, 29.–30.viii.1981, at light, Wiesner leg., 2 spec. (SMNS);

Aceh province: Mt. Bandahara, Bivouac Two, Serbolangit Range, 03°44'N 097°43'E, 1430 m, 5.–10.vii.1972, multistratal evergreen forest, J. Krikken leg., 1 male (RMNH);

Aceh province: Alas valley, Balelutu, ca. 03°43'N 097°38'E, 320 m, 3.–8.viii.1972, cultivated space in lowland multistratal evergreen forest, at light, J. Krikken leg. (nos. 36–40), 1 male (RMNH);

Aceh province: Alas valley, Ketambe, ca. 03°43'N 097°37'E, 17.–18.vi.1972, E.W. Diehl leg., 1 female (RMNH);

Sumatera Utara province: Deli [ca. 03°46'N 098°42'E], without date and collector’s name, 2 males, 3 females (ZMHB); same locality, without date, L. Martin leg., 1 male, 1 female (ZMHB); Dehli [= Deli], without date and collector’s name, 1 female (NHRS);

Sumatera Utara province: “Sumatra’s O. K.” [= Oostkust], Bedagei int. [= Sungai Bedagei, ca. 03°30'N 099°12'E], ca. 600 ft. [ca. 183 m], 1889, without collector’s name, 1 female (HNHM);

same locality, 2nd half of 1889, without collector’s name, 1 female (MNHN); same locality, 2nd half of 1889, without collector’s name, ex coll. R. Oberthür, 1 female (MNHN); same locality, 2nd half of 1889, without collector’s name, ex coll. A. Grouvelle, 5 females (MNHN); same locality, 2nd half of 1889, I. Z. Kannegieter leg., ex coll. R. Oberthür, 2 females (MNHN); same locality, 2nd half of 1889, I. Z. Kannegieter leg., ex coll. A. Grouvelle, 2 males, 2 females (MNHN); same locality, 2nd half of 1889, I. Z. Kannegieter leg., 2 males, 4 females (SMTD);

<u>Sumatera Utara province</u>: Brastagi [= Berastagi, ca. 03°11'N 098°31'E], Radiostat. Panorama, 1700 m, 2.viii.1972, Erber leg., light trap, 1 male, 1 female (SMNS); Brastagi [= Berastagi, ca. 03°11'N 098°31'E], 15.viii.1972, Erber leg., 1 spec. (SMNS); same locality, 9.iii.1973, Diehl leg., 2 spec. (SMNS);

<u>Sumatera Utara province</u>: Dolok Merangir [ca. 03°08'N 099°08'E], 1969, without collector’s name, 1 male (NHMW); same locality, 6.ix.1969, E.W. Diehl leg., 1 female (SMFD); same locality, 24.i.–2.ii.1971, E.W. Diehl leg., 1 female (SMFD); same locality, 11.iv.1971, E.W. Diehl leg., 1 male (MHNG); same locality, iv.1976, without collector’s name, 3 spec. (SMNS); same locality, 6.–30.v.1982, Diehl leg., 1 spec. (SMNS); same locality, “Holzweg”, 27.iii.1984, G. Hangay leg., 1 female (HNHM);

<u>Sumatera Utara province</u>: 18 km W Merek [ca. 02°56'N 098°31'E], 1800 m, 19.iv.1998, Cate & Nagy leg., WB det., 11 spec. (WBAC);

<u>Sumatera Utara province</u>: 8 km W Sidikalang [ca. 02°45'N 098°15'E], 1250 m, 12.v.1998, Cate & Nagy leg., WB det., 10 spec. (WBAC);

<u>Sumatera Utara province</u>: 8 km S Prapat [ca. 02°36'N 098°56'E], Sitahoan, 1400 m, 15.xi.1973, Diehl leg., 1 male (SMNS);

<u>Sumatera Utara province</u>: Tele, Dairi mts [ca. 02°27'N 098°43'E], 30.viii.1970, Diehl leg., 1 female (MHNG); same locality, 1500 m, 22.viii.1981, at light, Wriesres leg., 1 spec. (SMNS);

<u>Sumatera Utara province</u>: [Danau Toba lake, Lintong mts], Mt. Sanggul [ca. 02°15'N 098°44'E], 1250 m, iii.–iv.2005, without collector’s name, MN det., 2 spec. (MNIC);

<u>Sumatera Utara province</u>: 20 km NE P.-Siantar [ca. 02°08'N 098°46'E], 1000 m, 24.ix.1986, D. Erber leg., at light, 3 spec. (SMNS);

<u>Sumatera Utara province</u>: Siantar env. [ca. 01°58'N 098°47'E], Pasar Manduge, 30.viii.1979, Erber leg., 1 female (SMNS);

<u>Sumatera Utara province</u>: Sipirok [ca. 01°37'N 099°16'E], 20.ii.1996, M. Lödl leg., 1 male (NHMW);

<u>Sumatera Utara province</u>: Nias Island, Hili Madjedja [= Hili Maziaya, ca. 01°24'N 097°24'E], 1895, L.Z. Kannegieter leg., 1 male, 1 female (IRSNB); same data, ex coll. R. Oberthür, 1 female (MNHN); same data, ex coll. L. Bedel, 2 females (MNHN); same data, ex coll. A. Grouvelle, 2

males (MNHN);

Riau province: Sumatra, Rumbai-Minas [ca. 00°42'N 101°26'E], 1972–1977, W.C. Harrington leg., 1 female (CAS);

Riau province: Sumatra, Rumbai [ca. 00°35'N 101°26'E], 5.ix.1974, W.C. Harrington leg., 1 male (CAS);

Sumatera Barat province: “Sumatra’s W.K.”, Batang Paloepoeh [= Palupuh, ca. 00°08'S 100°18'E], 900 m, 23.xii.1931, on *Amorphophallus titanum* Becc., E. Jacobson leg., 15 males, 18 females (ZMAN); same locality, 900 m, 23.xii.1931, E. Jacobson leg., 47 spec. (RMNH);

Sumatera Barat province: Pajakoemboeh [= Payakumbuh, ca. 00°13'S 100°37'E], ix.1939, J. Kool leg., 1 male (ZMAN); Payakumbuh, v.1995, native collector, JS det., 1 female (JSCC);

Sumatera Barat province: Payakumbuh env., Harau valley [ca. 00°07'S 100°38'E], 500–800 m, 3.–4.2005, without collector’s name, 1 male (JRUC);

Sumatera Barat province: Fort de Kock [= Bukittinggi, ca. 00°18'S 100°21'E], 920 m, xii.1931, Jacobson leg., in flowers of *Amorphocephallus titanum*, 2 males, 1 female (SMTD); same locality, 1935, M. E. Walsch leg., 1 female (MNHN); same locality, 1000 m, vi.1939, J. Kool leg., 1 female (ZMAN);

Sumatera Barat province: Annai valley Natural Reserve, Mt. Singgalang [ca. 00°23'S 100°20'E], 500–1000 m, iv.2005, without collector’s name, 2 males, 1 female (JRUC);

Sumatera Barat province: Padang Panjang [ca. 00°27'S 100°24'E], v.1995, native collector, JS det., 1 female (JSCC); same locality, viii.1995, native collector, JS det., 1 female (JSCC);

Sumatera Barat province: Solok district [ca. 00°48'S 100°38'E], Mt. Intan, 900 m, ii.2006, S. Jakl leg., 1 female (JRUC); Solok district, Mt. Talang, 1200–1500 m, iii.2006, S. Jakl leg., 1 female (JRUC);

Sumatera Barat province: Kepulauan Mentawai islands, Siberut island, N part [ca. 00°56'S 098°51'E], 50–100 m, iii.–iv.2005, without collector’s name, 5 males, 5 females (JRUC);

Sumatera Barat province: NE Barus [= Batangbarus, ca. 01°01'S 100°38'E], Pakkat, 600 m, 12.ix.1972, Erber leg., light trap, 1 spec. (SMNS);

Sumatera Barat province: N Korintji vallei [= Kerinci, ca. 01°39'S 101°08'E], 5000 [ft.] [ca. 1525 m], ix.–x.1921, F.J. Pratt leg., 7 males, 12 females (ZMAN); Kerinci [= Kerinci Seblat National Park, ca. 01°48'S 101°07'E], ix.1995, native collector, JS det., 1 female (JSCC);

Bengkulu province: Benkoelen [= Bengkulu, ca. 03°47'S 102°15'E], Lebong Tanday, vi.1927, C.J. Louwerens leg., 1 male (RMNH);

Sumatera Selatan province: Palembang [ca. 03°00'S 104°45'E], without date and collector’s name, 1 female (IRSNB); same locality, without date, ex coll. R. Oberthür, 2 females (MNHN); same locality, without date, ex coll. L. Bedel, 2 females (MNHN); same locality, without date, M.

Knappert leg., ex coll. Veth, 1 male, 1 female (RMNH);
Lampung province: Lampongs [= Bandar Lampung, ca. 05°27'S 105°16'E], without date and collector’s name, 1 female (SDEI);
Special Capital Region of Jakarta: Tandjong [= Groeneveld, ca. 06°18'S 106°51'E], without date, Morawa leg., 1 male (SDEI);
Jawa Barat province: Buitenzorg [= Bogor, ca. 06°35'S 106°47'E], viii.1931, from dead pig, W.C. van Heurn leg., 7 males, 5 females (RMNH);
Jawa Barat province: Mt. Pangrango [ca. 06°46'S 106°58'E], 3000 m, without date and collector’s name, 2 females (SDEI);
Jawa Barat province: Gede [= Gede Pangrango Nat. Park, ca. 06°47'S 106°58'E], without date and collector’s name, MN det., 2 spec. (MNIC);
Jawa Barat province: Malabar Geb., Kina-Ondern. distr., Tjinjiroean [= Cinyiruan, ca. 07°10'S 107°36'E], 1700 m, xii.1909, ex coll. H.W. v.d. Weele, 1 male (RMNH);
Bali province: Bedugul Lag., Tamblingan lakes env. [ca. 08°15'S 115°06'E], 1200 m, 18.–22.ii.2004, S. Jakl leg., 1 female (JRUC); Tamblingan lake, 1200 m, iii.2004, without collector’s name, 2 males, 2 females (JRUC); Tamblingan lakes Natural Reserve, 1200–1450 m, vi.2009, 1 female (JRUC);
Nusa Tenggara Barat province: Lombok Island, Boun Pusuk [ca. 08°29'S 116°05'E], 200 m, 29.–30.iv.1986, trap (maggots bred in a trap), M. Nishikawa leg., MN det., 1 spec. (MNIC); Lombok Island, i.2000, without collector’s name, MN det., 1 spec. (MNIC);
Nusa Tenggara Barat province: Lombok Island, S Bentek vill., N slopes of Mt. Rinjani, Pusuk hill [ca. 08°28'S 116°30'E], 700–800 m, 16.–22.iii.2009, S. Jakl leg., 2 males, 3 females (JRUC);
Nusa Tenggara Timur province: Alor Island, Moru env. [ca. 08°15'S 124°30'E], 500 m, 22.iii.–3.iv.2006, S. Jakl leg., 7 males, 4 females (JRUC);
Nusa Tenggara Timur province: Pantar Island, Tanah Labang env. [ca. 08°19'S 124°16'E], 350 m, 9.–21.iii.2006, S. Jakl leg., 3 males (JRUC);
Nusa Tenggara Timur province: Flores Island, v.1930, W.C. van Heurn leg., 1 male, 4 females (RMNH); same data, ex coll. S.J. v. Ooststroom, 2 males, 1 female (RMNH); same data, ex coll. C. v. Nidek, 2 males (ZMAN); “N.O. Indië, Eiland Flores”, v.1930, W.C. van Heurn leg., 1 male, 3 females (RMNH); Flores Island, Ruteng area [ca. 08°36'S 120°27'E], Mt. Kuwus, 400–800 m, xii.2004, without collector’s name, 1 female (JRUC);
Nusa Tenggara Timur province: Timor Island, 50 km S Kupang, Buraen [ca. 10°17'S 123°50'E], 350 m, 26.i.–9.ii.2006, S. Jakl leg., 2 males (JRUC); same locality, 300 m, 7.–14.iv.2007, S. Jakl leg., 1 female (JRUC); 60 km S Kupang, Buraen, 350 m, 10.–21.ii. 2006, S. Jakl leg., 2 males, 1 female (JRUC);

Nusa Tenggara Timur province: Lesser Sundas, West Timor, Soe Region [ca. 09°51'S 124°17'E], Kolon vill. env., iv.2014, local collector leg., 1 male, 1 female (JRUC);
Sulawesi Utara province: Gng. Ambang F. R. near Kotamobagu [ca. 00°43'N 124°17'E], 1200 m, xi.1985, R. Entomol. Soc. London "Project Wallace", 1 female (BMNH); 20 km E of Kotamobagu, Mt. Ambang Nat. Reserve, 1190 m, 3.–8.xi.1985, scrub and shortgrass, at light, J. Krikken leg. (pw6), 1 male (RMNH);
Sulawesi Utara province: Dumoga-Bone Nat. Park [= Bogani Nani Wartabone National Park, ca. 00°33'S 123°40'E], Edwards Camp, 644 m, 8.viii.1985, "Project Wallace", Chen Young leg., 1 female (CMNH); same locality, Hog's Back' camp, 492 m, ix.1985, R. Entomol. Soc. London "Project Wallace", 2 males, 2 females (BMNH); same locality, 560 m, 17.–18.xi.1985, multistratal evergreen forest, at light, J. Krikken leg. (pw26), 1 male, 1 female (RMNH); same locality, xii.1985, lowland forest, R. Entomol. Soc. London "Project Wallace", 1 male, 2 females (BMNH); same locality, xii.1985, R. Entomol. Soc. London "Project Wallace", at light, 2 males, 6 females (BMNH);
Sulawesi Tengah province: Morowali env. [ca. 01°52'S 121°30'E], Ranu river area, 27.i.–20.iv.1980, M.J.D. Brendell leg., lowland rain forest, at UV light, 5 males, 3 females (BMNH);
Sulawesi Selatan province: E of Mamuju Makki [ca. 02°41'S 118°52'E], v.1999, native collector, JS det., 2 females (JSCC);
Sulawesi Selatan province: Rantepao [ca. 02°59'S 119°54'E], x.1989, on light, T. Baer leg., 1 female (MHNG); Rantepao, T. Jaya, 4.vi.1984, M. Tao leg., 3 male, 1 female (NSMT);
Sulawesi Selatan province: Palopo [ca. 03°05'S 120°14'E], 1400 m, ii.1995, [V.] Siniaev leg., JS det., 1 male (JSCC);
Sulawesi Selatan province: Punchak [ca 05°17'S 120°07'E], v.2000, native collector, JS det., 1 female (JSCC);
Maluku province: Bacan Island, SE slopes of Mt. Sibela, 5 km SE Makian [ca. 00°44'S 127°34'E], 500–750 m, 2.–12.v.2008, S. Jakl leg., 2 males, 3 females (JRUC);
Maluku province: Halmahera Island, "Noord Halmaheira", without date, Bernstein leg., 1 male (RMNH); Halmahera Island, 15 km SE Baru, Mt. Talagaranu, 01°12'N 127°32'E, 600 m, 22.–31.i.1996, primary forest, V. Siniaev & E. Tarasov leg., JS det., 6 males, 11 females (JSCC); same data, 1 male, 2 females (JRUC);
Maluku province: Morotai Island [ca. 02°19'N 128°26'E], without date, Bernstein leg., 1 male (RMNH);
Maluku province: Seram Island, 12 km SE Wahai Solea [ca. 02°48'S 129°22'E], 31.x.–4.xi.1998, J. Horák leg., JS det., 1 female (JSCC);
Maluku province: Seram Island, Manusela Nat. Park, Wae Mual plain [ca. 02°48'S 129°36'E],

25.vii.–2.ix.1987, Op.[eration] Raleigh, M.J.D. Brendell leg., 2 females (BMNH);

Maluku province: C Moluccas, Seram Is., West Seram Regency, Sahulau env. [ca. 03°02'N 128°43'E], 400 m, 5.–17.v.2013, local collector leg., 1 female (JRUC);

Maluku province: Buru [= Buru Island], 18.ix.–2.x.1921, station 14, rotting babirusa [*Babyrousa babyrussa* (Linnaeus, 1758), Mammalia: Suidae], L.J. Pratt leg., 2 males, 29 females (ZMAN); Buru, 25.ix.1921, station 14, rotten ananas and starfruit, L.J. Toxopeus leg., 3 males, 1 female (ZMAN); Buru, Mada Range [=Kapalamadan Mt., ca. 03°16'N 126°12'E], 850–1100 m, iv.–vi.1922, Pratt leg., 2 males, 1 female (ZMAN); E Buru, Ilath env., Remaja Mt. [ca. 03°33'S 127°11'E], 200–350 m, 5.–18.i.2013, St. Jakl leg., 1 male, 3 females (JRUC); same locality, 200 m, 7.–14.i.2013, J. Horák leg., 1 male (JRUC); same locality, 200–350 m, iii.2013, St. Jakl leg., 1 male (JRUC); same locality, 50–350 m, iii.2014, local collector leg., 1 female (JRUC); same locality, 0–400 m, i.2016, local collector leg., 1 male, 9 females (JRUC);

Maluku province: Ambelau Island [ca. 03°50'S 127°11'E], vii.2017, local collector leg., 1 female (JRUC);

Maluku province: Ambon Island, Ambaina [= Ambon, ca. 03°42'S 128°09'E], without date and collector's name, 1 male (ZMAN);

Maluku province: Ambon Island, Waai [ca. 03°33'S 128°18'E], 8.vii.1962, light trap, A.M.R. Wegner leg., 1 female (BPBM);

Maluku province: Tanimbar Islands, Yamdena Island, 5 km NW Lorulun village [ca. 07°50'S 131°18'E], 150 m, 6.ii.–30.iii.2007, S. Jakl leg., 1 male (JRUC);

Papua Barat province: Sorong [ca. 00°52'S 131°17'E], 29.ix.–6.x.1992, B. Balázs leg., 1 male, 1 female (HNHM);

Papua Barat province: Manokwari Regency, Arfak Mts., Maibri village [ca. 01°07'S 133°54'E], 1570 m, 6.–19.xi.2012, J. Horák leg., 2 males (JRUC); Maibri env., 1670 m, 28.i.–5.ii.2014, local collector leg., 1 female (JRUC);

Papua Barat province: 30 km S Manokwari [ca. 01°08'S 134°03'E], Arfak mts, Ngat Biep river, Ngat valley, 950 m, 18.–19.xii.1993, R. Brechlin & K. Černý leg., JS det., 1 female (JSCC);

Papua Barat province: Manokwari Regency, Arfak mts [ca. 01°09'S 133°59'E], ca. 20 km S of Warmere, Duebei, 1190 m, 21.i.–8.ii.2008, S. Jakl leg., 2 males, 2 females (JRUC);

Papua Barat province: S of Manokwari, NW Oransbari [ca. 01°20'S 134°15'E], xii.1962, L. Richards leg., 1 male, 1 female (BPBM);

Papua Barat province: Fakfak, airport [ca. 02°56'S 132°18'E], 17.vii.1996, Schüle & Stüben leg., 1 male (SMNS); Fakfak, xii.2004, without collector's name, 1 male (JRUC);

Papua province: "Nouv.-Guinée", I.[sland] Mefoor [= Numfoor Island, ca. 01°00'S 134°53'E], 1878, Raffray & Maindron leg., 5 males, 10 females (MNHN); same data, ex coll. L. Bedel, 1 male

(MNHN);

Papua province: Bodem, Sarmi Area [ca. 01°51'S 138°45'E], 10.vii.1959, T.C. Maa leg., 5 females (BPBM);

Papua province: Bodem, 11 km SE of Oerberfaren [ca. 01°58'S 138°44'E], 100 m, 7.–17.vii.1959, T.C. Maa leg., 5 females (BPBM);

Papua province: "Dutch N. Guinea", Maffin Bay [ca. 01°58'S 138°52'E], 20.viii.1944, E.S. Ross leg., 1 male (CAS);

Papua province: River Tor (mouth), 4 km E of Hol Maffen [ca. 01°58'S 138°54'E], 2.vii.1959, at light, T.C. Maa leg., 1 female (BPBM);

Papua province: Cyclops Mts., Depapre [ca. 02°27'S 140°21'E], 500 m, 26.ix.1993, H.J.G. van Mastricht leg., 1 male (ZMAN);

Papua province: Ifar, Cyclops Mts. [ca. 02°32'S 140°31'E], 300–500 m, 23.–25.vi.1962, light trap, J.L. Gressitt & J. Sedlacek leg., 7 males, 3 females (BPBM); same locality, 300 m, 26.vi.1962, Malaise trap, Gressitt & Maa leg., 2 males, 1 female (BPBM); same locality, 350 m, 28.vi.1962, mercury vapor light trap, J. Sedlacek leg., 11 males, 15 females (BPBM);

Papua province: Hollandia [= Jayapura, ca. 02°32'S 140°42'E], 1911, Ned. Nw. Guinea Exped., P.N. v. Kampen leg., 1 female (RMNH); "Dutch New Guinea", Humboldt Bay distr. [= Yos Sudarso Bay near Jayapura], 1937, W. Stüber leg., 1 male, 1 female (BMNH);

Papua province: Sentani [ca. 02°34'S 140°30'E], 90 m, 22.vi.1959, mercury vapor light trap, Gressitt & Maa leg., 1 male (BPBM);

Papua province: Nabire, Pusppensaat (base camp 54 km) [ca. 03°24'S 135°20'E], without date, A. Riedel leg., KH det., 1 male (KHAC);

Papua province: Naribe, Irian Jaya highway, km 45, 03°29'52"S 135°43'84"E, 745 m, 22.–24.xi.1997, primary forest, K. Černý leg., JS det., 1 female (JSCC);

Papua province: Dogyiai Regency, Mapia env. [ca. 03°55'S 135°52'E], xii.2006, local collector leg., 1 male, 1 female (JRUC);

Papua province: "Irian Jaya", Star mountains, Mabilabol, Kec. Oksibil [ca. 04°54'S 140°37'E], 1240 m, 17.–21.ix.1990, H.J.G. van Mastrigt leg., 2 males (ZMAN);

Papua province: Dofura [not located], 8.x.1959, E.R. Leach leg., 1 male (CAS);

Not or imprecisely located: "Irian Jaya" [= Papua Barat], viii.–ix.1944, M. Marquis leg., 1 male (CAS);

"Battaklanden [= part of Northern Sumatra] deb. 2276", without date and collector's name, 1 female (SDEI);

Papua New Guinea: East Sepik province: Ulop [= Ulap, ca. 03°53'S 142°27'E], vii.–viii.1965, H. Pyka leg., 1 male (SMNS);

East New Britain province: New Britain, Ralum [Gazelle Peninsula, ca. 04°19'S 152°14'E], without date, F. Dahl leg., 4 males, 4 females (ZMHB); same locality, 30.v.1896, F. Dahl leg., 1 male (ZMHB);

East New Britain province: Bismarck Archipelago: New Britain Island, Yalom [ca. 04°25'S 151°45'E], 1000 m, 9.v.1962, Noona Dan Expedition 61–62, M. Mroczkowski det., 1 male (ZMUC);

East Sepik province: Amboin [ca. 04°36'S 143°29'E], without date and collector's name, 1 female (NHRS);

Madang province: Astrolabe-B[ay] [ca. 05°14'S 145°47'E], without date, Rhode [leg.?], 1 male (ZMHB); Astrolabe Bay, without date (but before 1917), ex coll. A. Grouvelle, 1 female (MNHN);

Madang province: Friedrich-Wilhelms-hafen [= Madang, ca. 05°14'S 145°47'E], 1896, Biró leg., 1 male (HNHM);

Madang province: Baiteta [ca. 05°01'S 145°45'E], 15.vi.1996, O. Missa leg., canopy mission, light trap, AR22, 1 male, 1 female (IRSNB);

Madang province: 10 km S Siniap, 5.22757°S 145.07997°E, 160 m, 26.xi.–5.xii.2019, O. Nakládal leg., on light, 1 male, 1 female (JRUC);

Eastern Highlands province: Kainantu env. [ca. 06°17'S 145°52'E], Onerunka leg., vii.1981, 1 male (MHNG);

Morobe province: Finschhafen [ca. 06°36'S 147°51'E], Heldsbach, xi.1979, W.G. Ullrich leg., 6 females (MHNG);

Morobe province: Buso river [ca. 06°40'S 147°12'E], 4.x.1979, J.H. Martin leg., 1 female (BMNH); same locality, 13.x.1979, 1 male (BMNH);

Morobe province: Lae env., Gurakor [= Gurukor, ca. 06°50'S 146°38'E], 7.vii.1965, H. Pyka leg., 2 spec. (SMNS);

Morobe province: Bulow [= Bulowat, ca. 07°05'S 146°38'E], without date and collector's name, WB det., 1 spec. (WBAC);

Morobe province: Bulolo [ca. 07°12'S 146°37'E], 700 m, without date, Sedlacek leg., 1 male, 1 female (HNHM); same locality, without date and collector's name, WB det., 1 spec. (WBAC); same locality, 1934, ex coll. M. Vaněk, 1 female (NMPC); same locality, 1967, W.J. Smith leg., JH det., 1 male (JHAC); same data, 1 male, 1 female (JRUC);

Morobe province: Wau, Mt. Missim [ca. 07°19'S 146°48'E], 880–1050 m, 8.–9.ii.1963, J. Sedlacek leg., 1 male (BPBM); same locality, 950–1300 m, xii.1965, J. Sedlacek leg., 4 females (BPBM);

Morobe province: Mt. Kaindi [ca. 07°20'S 146°41'E], 2350 m, 30.iv.1969, moss forest, on small Rubus, J. Szent-Iivany leg., 1 female (BPBM);

Morobe province: Wau [ca. 07°20'S 146°43'E], 1200 m, 15.–25.x.1961, mercury vapor light trap, J.

Sedlacek leg., 1 male, 3 females (BPBM);
same locality, 29.x.1961, mercury vapor light trap, J. Sedlacek leg., 2 females (BPBM);
same locality, 1.–20.xi.1961, mercury vapor light trap, J. Sedlacek leg., 4 males, 7 females (BPBM);
Wau, 1050 m, 11.xi.1961, light trap, J. & M. Sedlacek leg., 1 male (BPBM);
Wau, 1200 m, 2.xii.1961, J. Sedlacek leg., 1 male (BPBM);
same locality, 7.–16.xii.1961, mercury vapor light trap, J. Sedlacek leg., 1 female (BPBM);
Wau, 2500 m, 28.xii.1961, mercury vapor light trap, J. Sedlacek leg., 1 male (BPBM);
Wau, 1200 m, 15.v.1962, J. Sedlacek leg., 1 male, 1 female (BPBM);
same locality, 10.–19.v.1962, mercury vapor light trap, J. Sedlacek leg., 1 male (BPBM);
same locality, 10.–19.vi.1962, light trap, J. Sedlacek leg., 1 male, 1 female (BPBM);
same locality, 20.–26.v.1962, light trap, J. Sedlacek leg., 1 female (BPBM);
same locality, 26.ix.1962, Malaise trap, J. Sedlacek leg., 1 male (BPBM);
same locality, 1.–4.x.1962, Malaise trap, J. Sedlacek leg., 4 males, 2 females (BPBM);
same locality, xii.1962, without collector's name, 1 male (AMNH);
same locality, 3.–4.i.1963, J. Sedlacek leg., 1 male, 1 female (BPBM);
same locality, 31.i.1963, J. Sedlacek leg., 1 female (BPBM);
same locality, 7.ii.1963, J. & M. Sedlacek leg., 1 female (BPBM);
same locality, 1.–3.iii.1963, mercury vapor light trap, J. Sedlacek leg., 2 females (BPBM);
same locality, 10.iii.1963, mercury vapor light trap, J. Sedlacek leg., 1 male, 1 female (BPBM);
same locality, 12.iii.1963, mercury vapor light trap, J. Sedlacek leg., 1 male (BPBM);
same locality, 23.iii.1963, mercury vapor light trap, J. Sedlacek leg., 2 females (BPBM);
same locality, 31.iii.1963, light trap, J. Sedlacek leg., 1 male, 1 female (BPBM);
same locality, 11.–17.iv.1963, mercury vapor light trap, J. Sedlacek leg., 1 male (BPBM);
same locality, 28.–29.vii.1963, J. Sedlacek leg., 2 males (BPBM);
same locality, 30.–31.vii.1963, J. Sedlacek leg., 1 male, 1 female (BPBM);
same locality, 1.–9.viii.1963, J. Sedlacek leg., 1 male (BPBM);
same locality, 11.–12.viii.1963, J. Sedlacek leg., 1 female (BPBM);
same locality, 15.–16.viii.1963, J. Sedlacek leg., 1 female (BPBM);
same locality, 17.viii.1963, mercury vapor light trap, J. Sedlacek leg., 9 males, 15 females (BPBM);
same locality, 1.–3.x.1963, mercury vapor light trap, J. Sedlacek leg., 13 males, 8 females (BPBM);
same locality, 2.–5.iii.1964, mercury vapor light trap, J. Sedlacek leg., 2 males (BPBM);
Wau, 1000–1250 m, 3.iii.1964, J. Sedlacek leg., 1 female (BPBM);
Wau, 1200 m, 2.–5.iii.1964, mercury vapor light trap, J. Sedlacek leg., 1 male (BPBM);
same locality, 5.–13.iii.1964, mercury vapor light trap, J. Sedlacek leg., 3 males, 5 females

(BPBM);
same locality, 14.–24.iii.1964, mercury vapor light trap, J. Sedlacek leg., 11 males, 9 females (BPBM);
same locality, 26.iii.1964, J. Sedlacek leg., 8 males, 3 females (BPBM);
same locality, 26.iii.–2.iv.1964, J. Sedlacek leg., 3 females (BPBM);
same locality, 1.–2.iv.1964, J. Sedlacek leg., 3 males, 3 females (BPBM);
same locality, 3.–7.iv.1964, mercury vapor light trap, J. Sedlacek leg., 5 males, 9 females (BPBM);
same locality, 11.iv.1964, J. Sedlacek leg., 1 female (BPBM);
same locality, 11.–12.iv.1964, light trap, J. & M. Sedlacek leg., 1 male, 3 females (BPBM);
same locality, 25.–30.iv.1964, mercury vapor light trap, J. Sedlacek leg., 3 males, 2 females (BPBM);
same locality, 16.viii.1964, mercury vapor light trap, J. Sedlacek leg., 1 male (BPBM);
same locality, 2.–5.iii.1964, mercury vapor light trap, J. Sedlacek leg., 1 female (BPBM);
same locality, 5.–13.iii.1964, mercury vapor light trap, J. Sedlacek leg., 1 male, 2 females (BPBM);
same locality, 14.–24.iii.1964, mercury vapor light trap, J. Sedlacek leg., 2 males, 4 females (BPBM);
same locality, 18.–20.viii.1964, mercury vapor light trap, J. Sedlacek leg., 1 male (BPBM);
same locality, 14.ix.1964, J. Sedlacek leg., 1 female (BPBM);
same locality, 23.ix.1964, mercury vapor light trap, J. Sedlacek leg., 1 female (BPBM);
same locality, 23.–24.ix.1964, mercury vapor light trap, J. Sedlacek leg., 2 males (BPBM);
same locality, 25.–30.ix.1964, mercury vapor light trap, J. Sedlacek leg., 6 males, 7 females (BPBM);
same locality, 26.–27.ix.1964, mercury vapor light trap, J. Sedlacek leg., 1 female (BPBM);
Wau, 1100–1300 m, 12.x.1964, J. & M. Sedlacek leg., 3 males (BPBM);
same locality, 13.x.1964, mercury vapor light trap, J. & M. Sedlacek leg., 1 male (BPBM);
Wau, 1200 m, 27.x.1964, mercury vapor light trap, J. & M. Sedlacek leg., 1 female (BPBM);
same locality, 30.–31.x.1964, mercury vapor light trap, J. Sedlacek leg., 1 female (BPBM);
Wau, 1000–1200 m, 27.–28.xi.1964, mercury vapor light trap, J. Sedlacek, 1 male, 1 female (BPBM);
Wau, 1200 m, 2.xii.1964, mercury vapor light trap, J. & M. Sedlacek leg., 2 females (BPBM);
Wau, Big Wau Creek, 1100 m, 29.xi.–30.xii.1964, mercury vapor light trap, Shanahan leg., 1 female (BPBM);
Wau, 1200 m, 30.xii.1964, mercury vapor light trap, J. & M. Sedlacek leg., 2 males, 1 female (BPBM);
Wau, Big Wau Creek, 1100 m, 31.xii.1964, mercury vapor light trap, J. & M. Sedlacek leg., 3

males, 2 females (BPBM);
Wau, 1200 m, 4.–15.i.1965, J.H. & M. Sedlacek leg., 1 male, 5 females (BPBM);
Wau, Hospital Creek, 1200 m, 18.i.1965, J. Sedlacek leg., 3 females (BPBM);
same locality, 9.–13.iv.1965, Malaise trap, J. Sedlacek leg., 1 male (BPBM);
Wau, Bishop Museum Field Station, 15.–25.iv.1965, J. Balogh & J.J. Szent-Ivány leg., 1 male, 2 females (HNHM);
Wau, 1200 m, 2.v.1965, mercury vapor light trap, J. & M. Sedlacek leg., 1 male (BPBM);
same locality, 6.vii.1965, mercury vapor light trap, J. & M. Sedlacek leg., 1 male, 1 female (BPBM);
same locality, 9.vii.1965, J. Sedlacek leg., 3 males, 4 females (BPBM);
same locality, 17.vii.1965, mercury vapor light trap, J. & M. Sedlacek leg., 1 male (BPBM);
Wau, 1200–1300 m, 13.vii.1965, mercury vapor light trap, J. & M. Sedlacek leg., 1 male, 1 female (BPBM);
same locality, 14.viii.1965, mercury vapor light trap, J. & M. Sedlacek leg., 4 males (BPBM);
same locality, 15.viii.1965, mercury vapor light trap, J. & M. Sedlacek leg., 3 males, 1 female (BPBM);
same locality, 21.viii.1965, mercury vapor light trap, J. & M. Sedlacek leg., 4 males, 3 females (BPBM);
Wau, Big Wau Creek, 1200 m, ix.1965, J. & M. Sedlacek leg., 4 males, 1 female (BPBM);
same locality, 1.–9.ix.1965, J. Sedlacek leg., 6 males, 4 females (BPBM);
Wau, 1200 m, 5.ix.1965, mercury vapor light trap, J. & M. Sedlacek leg., 1 male, 1 female (BPBM);
Wau, 1200–1500 m, 30.ix.1965, mercury vapor light trap, J. & M. Sedlacek leg., 1 male (BPBM);
Wau, Big Wau Creek, 1200 m, 26.x.1965, mercury vapor light trap, J. & M. Sedlacek leg., 1 male, 4 females (BPBM);
Wau, 4000 ft. [= ca. 1220 m], 29.x.1965, at mercury vapor light, Field No. 1230, D.H., A.C. & A.H. Kistner leg., 1 female (BPBM); same data, 30.x.1965, Field No. 1233, 2 females (BPBM); same data, 1.xi.1965, 3 males (BPBM); same data, 4.xi.1965, 1 female (BPBM);
Wau, 1200 m, 11.xii.1965, mercury vapor light trap, J. & M. Sedlacek leg., 1 female (BPBM);
same locality, 21.–25.xii.1965, J. Sedlacek leg., 1 female (BPBM);
Wau, Big Wau Creek, 1200 m, xii.1965, Malaise trap, J. Sedlacek leg., 1 male (BPBM);
Wau, 1100–1500 m, i.1966, J. Sedlacek leg., 1 male (BPBM);
Wau, 1100–1300 m, 2.i.1966, L. & M. Gressitt leg., 1 male, 1 female (BPBM);
Wau, 1200 m, 7.i.1966, mercury vapor light trap, J. Sedlacek leg., 1 male, 1 female (BPBM);
Wau, 1150–1250 m, 25.i.1966, J. Sedlacek leg., 1 spec. (BPBM);

Wau, 1200–1700 m, 28.i.1966, J. Sedlacek leg., 2 males (BPBM);
Wau, 1200 m, ii.1966, mercury vapor light trap, J. Sedlacek leg., 2 males (BPBM);
same locality, 3.ii.1966, J. Sedlacek leg., 2 females (BPBM);
same locality, 6.ii.1966, mercury vapor light trap, J. Sedlacek leg., 1 female (BPBM);
same locality, 23.ii.1966, J. Sedlacek leg., 3 females (BPBM);
same locality, 14.iii.1966, light trap, Gressitt & Wilkes leg., 1 female (BPBM);
same locality, 14.iii.1966, L.J. Gressitt leg., 1 female (BPBM);
same locality, 25.iii.1966, light trap, Gressitt & Wilkes leg., 2 males, 1 female (BPBM);
same locality, 25.iii.1966, light trap, J.L. Gressitt leg., 1 male, 1 female (BPBM);
same locality, 28.–29.iii.1966, J.L. Gressitt leg., 3 males (BPBM);
same locality, iv.1966, light trap, J.L. Gressitt leg., 2 males, 2 females (BPBM);
same locality, 3.–7.iv.1966, mercury vapor light trap, J. Sedlacek leg., 1 male, 2 females (BPBM);
same locality, 12.iv.1966, mercury vapor light trap, J. Sedlacek leg., 3 males (BPBM);
same locality, 27.iv.1966, G.A. Samuelson leg., 1 female (BPBM);
same locality, 18.vi.1966, light trap, Gressitt & Wilkes leg., 1 female (BPBM);
same locality, 23.vi.1966, J.L. Gressitt leg., 1 male (BPBM);
same locality, 27.vi.1966, G.A. Samuelson leg., 1 male, 2 females (BPBM);
same locality, 16.–17.viii.1966, J. Sedlacek leg., 1 female (BPBM);
same locality, 21.xi.1966, light trap, J.L. Gressitt leg., 1 female (BPBM);
same locality, 27.xi.1967, mercury vapor light trap, J. Sedlacek leg., 1 male (BPBM);
same locality, 19.–21.iv.1968, J.L. Gressitt leg., 1 male (BPBM);
Wau, 1150 m, 25.vii.1968, J.J.H. & M.L. Szent-Ivany leg., 2 females (BPBM);
Wau, 15.x.1969, J. Sedlacek leg., ex coll. J.E. Tobler, 1 male, 2 females (CAS);
Wau, Bishop Museum Field Station, 1200 m, 22.–25.iv.1970, T.W. Davies & M.C. Davies leg., 1 female (CAS);
Wau, iii.1973, Zwick leg., light trap, 2 males, 1 female (MHNG);
Wau, Big Wau Creek, 1200 m, 10.iii.1973, T.W. Davies leg., 1 male (CAS);
Wau, 1200 m, ii.1974, J. Sedlacek leg., 1 female (ZMUC);
same locality, 26.vii.1974, mercury vapor light trap, A.D. Hort leg., K-1146, 1 female (BPBM);
Wau env., xii.1978, without collector's name, 1 female (MHNG);
Wau, 21.iii.1982, light trap, R.T. Bell leg., 1 female (BPBM);
same locality, 30.iii.–v.1982, light trap, R.T. Bell leg., 1 female (BPBM);
Wau, 1300 m, 25.x.–6.xi.1982, at U.V. light, W.C. & B.H. Gagné leg., 1 male (BPBM);
same locality, 4.xi.–12.xi.1982, at U.V. light, W.C. & B.H. Gagné leg., 1 male (BPBM);
<u>Morobe province</u>: Wa'j valley [= Wau?], 1200 m, viii.1996, ex coll. C.C. Chua, 1 male, 1 female

(NMPC);

Morobe province: Garaina [ca. 07°53'S 147°08'E], 900–1800 m, 15.–21.i.1968, J. & M. Sedlacek, 1 male, 1 female (BPBM);

Gulf province: Tekadu, 120 m, 07°38'S 146°34'E, 25.i.2000, T.A. Sears & binatung brigade leg., DSS det., 4 males, 4 females (UCDC); same locality, 18.iii.2000, T.A. Sears & binatung brigade leg., DSS det., 2 males, 5 females (UCDC); same locality, 28.iii.2000, T.A. Sears & binatung brigade leg., DSS det., 1 female (UCDC); same locality, 29.iii.2000, T.A. Sears & binatung brigade leg., DSS det., 1 male (UCDC); same locality, 26.iv.2000, T.A. Sears & T. David leg., DSS det., 1 male, 5 females (UCDC);

Gulf province: Ivimka Res. Station, Lakekamu Basin, 120 m, 07°44'S 146°30'E, 3.–15.xi.1999, S.L. Heydon, N. Schiff, T.A. Sears leg., DSS det., 18 males, 18 females (UCDC); same locality, 16.–25.xi.1999, T.A. Sears leg., DSS det., 2 males (UCDC); same locality, 19.–25.xi.1999, T.A. Sears leg., DSS det., 2 males, 5 females (UCDC); same locality, 22.ii.–1.iii.2000, T.A. Sears leg., DSS det., 7 males, 1 female ([UCDC); same locality, 5.iii.2000, T.A. Sears leg., DSS det., 4 males (UCDC); same locality, 10.iii.2000, T.A. Sears & binatung brigade leg., DSS det., 1 male, 3 females (UCDC); same locality, 15.–26.iii.2000, T.A. Sears & binatung brigade leg., DSS det., 7 males, 5 females (UCDC); same locality, 21.iii.2000, T.A. Sears & binatung brigade leg., DSS det., 2 females (UCDC); same locality, 28.iii.2000, T.A. Sears & binatung brigade leg., DSS det., 2 males, 3 females (UCDC); same locality, 29.iii.2000, T.A. Sears & binatung brigade leg., DSS det., 1 female (UCDC); same locality, 30.iii.2000, T.A. Sears & binatung brigade leg., DSS det., 1 male (UCDC); same locality, 31.iii.2000, T.A. Sears & binatung brigade leg., DSS det., 1 male (UCDC); same locality, 2.iv.2000, T.A. Sears leg., DSS det., 1 male, 2 females (UCDC); same locality, 3.iv.2000, T.A. Sears & binatung brigade leg., DSS det., 1 female (UCDC); same locality, 4.iv.2000, T.A. Sears leg., DSS det., 1 female (UCDC); same locality, 5.iv.2000, T.A. Sears leg., DSS det., 2 females (UCDC); same locality, 10.iv.2000, T.A. Sears leg., DSS det., 4 males, 2 females (UCDC);

Gulf province: Murua [ca. 07°55'S 145°48'E], 13.xi.1964, W. Steffan leg., 1 female (BPBM);

Northern province: Popondetta [ca. 07°20'S 146°41'E], 25 m, v.1966, light trap, Snanahan & Lippert leg., 1 female (BPBM); same data, vi.1966, 2 males, 1 female (BPBM);

Northern province: Kokoda [ca. 08°53'S 147°44'E], 400 m, 18.xi.1966, J. & M. Sedlacek leg., 1 male (BPBM);

Western province: Oriomo River [ca. 09°01'S 143°13'E], 6 m, 14.ii.1964, light trap, H.C. leg., 1 male (BPBM);

Milne Bay province: Woodlark Island [= Muyua Island, ca. 09°08'S 152°46'E], 1901, A.S. Meek leg., 1 male, 1 female (BMNH);

Milne Bay province: Fergusson Island [ca. 09°32'S 150°40'E], pre-1941, without collector's name, ex coll. Levick, 1 female (BMNH);
National Capital District: Port Moresby [ca. 09°28'S 147°11'E], without date and collector's name, 1 male (SMTD);
Not or imprecisely located: Fly river, 1876–1877, L.M. d'Albertis leg., 1 male, 1 female (ZMHB); same data, 3 males, 2 females (MNHN); same data, 2 females (ZMAS); same data, ex coll. E. Witte, 2 females (SMFD); same data, ex coll. A. Grouvelle, 1 male, 1 female (MNHN); same data, ex coll. Deharme, 2 f*f* (MNHN); same data, ex coll. Lokay, 1 female (NMPC);
"Neu-Guinea, Kaiser Wlh: [Wilhelms] Land, N. Guinea Comp. V", without date and collector's name, 1 male (ZMHB);
[Papua] New Guinea, Mafalu, without date [probably 1903], [A.E.] Pratt leg., ex coll. R. Oberthur, 1 m* (MNHN);
"Neuguinea", without date and collector's name, 1 female (SDEI);
Solomon Islands: New Georgia Island, Munda [ca. 08°19'S 157°14'E], 1944, L.A. Comvell leg., 1 female (NMNH);
Guadalcanal Is., Tenaru River [ca. 09°26'S 160°05'E], i.1945, G.E. Bohart leg., 1 male, 1 female (CAS);
Guadalcanal Is., Kukum [= Malaita Is., Kukumu, ca. 08°46'S 160°42'E], 19.xii.1964, P. Greenslade leg., 1 female (BMNH); same locality, iv.1965, 2 males, 1 female (BMNH);
Not or imprecisely located: "Iles Salomon", without more precise locality, without date and collector's name, 1 female (IRSNB);
Australia: Northern Territory: Darwin [ca. 12°27'S 130°50'E], without date, C.F. Hill leg., 1 female (AMNH); same locality, without date, ex coll. Deuquet, 1 female (CAS);
Queensland: Cape York Peninsula, Lockerbie [ca. 10°48'S 142°28'E], HQ, 7.–14.iv.1977, A. & M. Walford-Huggins leg., 1 male (BMNH);
Queensland: Cape York Peninsula, Brown's Creek in Pascoe River drainage basin [ca. 14°38'S 144°06'E], 200 ft. [= 60 m], 12.vii.1948, G.M. Tate leg., 1 male, 2 females (AMNH);
Queensland: 13 km W of Musgrave, 14°48'S 143°23'E, 220 m, 15.i.1994, G. Daniels, A. Daniels & A. Eastwood leg., MV lamp, MN det., 1 spec. (MNIC);
Queensland: Cape York Peninsula, Hann River Roadhouse, 15°01'S 143°52'E, 76 m, 27.xi.2010, S. Jakl leg., 1 male (JRUC);
Queensland: Endeavour River [ca. 15°25'S 145°05'E], without date and collector's name, 1 male, 1 female (HNHM);
Queensland: Daintree [ca. 16°15'S 145°19'E], ii.1999, without collector's name, MN det., 1 spec. (MNIC); same locality, i.2001, S. Lamond leg., 2 male (BMNH);

Queensland: Mt. Molloy [ca. 16°40'S 145°20'E], 21.vi.1970, A. & M. Walford-Huggins leg., 1 male (BMNH);

Queensland: Mareeba Shire, Kuranda Russet Park [ca. 16°40'S 145°28'E], 460 m, 22.xi.1987, T.W. Davies leg., mercury vapor light trap, 1 male (CAS); same data, 18.–26.xii.1987, 1 male (CAS);

Queensland: Cairns [ca. 16°55'S 145°45'E], without date, Hacker leg., 1 male, 1 female (SDIC); same locality, 14.ii.1909, G.E. Bryant leg., 1 male, 1 female (BMNH); same locality, 1918, carrion, J.F. Illingworth leg., 7 males, 4 females (BPBM); same locality, 1920, J.F. Illingworth leg., 2 females (CAS); same data, 18 males, 16 females (BPBM); Cairns, Kamerunga, 6.ix.1969, A. & M. Walford-Huggins leg., at MV light, 1 female (BMNH);

Queensland: Gordonvale [ca. 17°06'S 145°47'E], 1917, J.F. Illingworth leg., on carrion, 1 female (CAS); same data, 1 male, 2 females (BPBM);

Queensland: Atherton [ca. 17°16'S 145°28'E], without date [probably 1912–1913], [E.] Mjöberg leg., 9 males, 8 females (NHRS); same locality, 1919, ex coll. J.J. de Vos, 1 male, 1 female (RMNH); same locality, 1919, ex coll. J. Hlisnikovský, 1 male, 1 female (NMPC);

Queensland: Yungaburra [ca. 17°16'S 145°35'E], 23.iii.1965, G.F. Bornemissza leg., 1 female (HNHM);

Queensland: Innisfail [ca. 17°31'S 146°02'E], Polly Creek, 11.i.2000, K. Tazoe leg., 1 male (NSMT);

Queensland: 35 km W Innisfail, Wooroo-nooran Nat. Park, 17°36'S 145°41'E, 790 m, 10.–12.xii.2010, S. Jakl leg., 1 male (JRUC);

Queensland: Normantown [= Normanton, ca. 17°40'S 141°03'E], without date (but before 1917), Ch. French leg., ex coll. A. Grouvelle, 1 male (MNHN);

Queensland: Mackay [ca. 21°09'S 149°10'E], without date and collector's name, ex coll. E. Lokay, 1 male (NMPC); same locality, without date [but before 1927], without collector's name, ex coll. G. C. Champion, 1 female (BMNH);

Queensland: Carmila [ca. 21°54'S 149°25'E], 1.vii.1971, H.M.J. Sas leg., 1 male, 1 female (RMNH);

Queensland: Eidsvold [ca. 25°22'S 151°07'E], without date, Dr. T.L. Bancroft, 1 male, 1 female (SDEI);

Queensland: Brisbane [ca. 27°28'S 153°02'E], without date, ex coll. Hacker, 1 male (SDEI);

Queensland: Teviot Range [ca. 27°55'S 152°45'E], near Fliders-Peak, 23.ix.1971, L. Oosterweghel leg., 1 male (RMNH);

Queensland: McPherson Range, Lamington Nat. Park, Binna Burra, 28°11'S 153°11'E, 750 m, 5.–12.i.1992, D. Burckhardt leg., at light, 1 female (MHNG); Lamington Nat. Park, 11.–17.ii.1963, G. Monteith leg., 3 females (BPBM);

Queensland: Little Mangrove River [not located], without date and collector’s name, 2 females (CAS);
New South Wales: Mullumbimby [ca. 28°33'S 153°30'E], Cedar Rd., Huonbrook, 1.–15.ii.1991, T. Ralph leg., MVL, G. Hangay coll., KH det., 1 male, 1 female (KHAC);
New South Wales: Armidale [ca. 30°31'S 151°40'E], without date, C.F. Deuquet leg., 1 female (CAS);
New South Wales: Macleay River [ca. 30°51'S 152°27'E], v.1904, ex coll. Helms, 1 female (BPBM); same data, vii.1906, 3 males, 2 females (BPBM);
New South Wales: Blue Mountains [ca. 33°44'S 150°18'E], without date [but before 1922], without collector’s name, ex coll. L. Bedel, 2 males (MNHN);
New South Wales: Sydney [ca. 33°50'S 151°06'E], without date and collector’s name, ex coll. E. Lokay, 1 male (NMPC);
New South Wales: Sydney, Cabramatta [ca. 33°53'S 150°56'E], 16.iii.1959, M.I. Nikitin leg., 1 female (NSMT); same locality, 13.v.1959, M. Nikitin leg., 1 male (ZMAS);
New South Wales: Padstow [ca. 33°57'S 151°02'E], i.1924, Deuquet leg., 1 female (CAS);
Australian Capital Territory: Canberra [ca. 35°19'S 149°07'E], 650 m, 16.iii.1956, R. Staatman leg., 1 male (RMNH);
Victoria: Melbourne [ca. 37°49'S 144°57'E], without date [probably 1900–1901, Kořenský (1904)], J. Kořenský leg., 1 male (NMPC); same locality, without date and collector’s name, 1 male, 2 females (SMTD);
Not or imprecisely located: Goenoeng [= Gunung] Paukjar [illeg.], 16.v.1931, without collector’s name, ex coll. H. J. Klaasen, 1 male (ZMAN);
“Indien”, without more precise locality, date and collector’s name, ex coll. E. Boecker, 1 male (SMFD);
“Malaischer Archipel”, without more precise locality, date and collector’s name, ex coll. E. Witte, 1 male (SMFD);
“Malaysia”, without date, Chlopčík leg., 1 female (NMPC);
“Me Kong [= Mekong river]”, without more precise locality, without date, E. Heirne leg., 1 female (RMNH);
“Norsfried”, 1 female (NMPC);
“Sumatra”, without more precise locality, without date, without collector’s name, 2 males, 1 female (NMPC);
“Sumatra”, without more precise locality, without date [1854–1862], [A.R.] Wallace leg., ex coll. Fry, 1 male, 2 females (BMNH);
“Sumatra”, without more precise locality, without date, v. d. Bosche leg., 1 female (RMNH);

“Sumatra”, without more precise locality, without date, Ludeking leg., 1 male, 1 female (RMNH);
“Sumatra”, without more precise locality, without date, Muller leg., 1 male, 1 female (RMNH);
“Sumatra”, without more precise locality, without date, Heyne leg., coll. v. Schönfeld, 1 male (SMFD);
“Sumatra”, without more precise locality, without date, Klein leg., 1 male, 1 female (ZMUC);
“Sumatra, Museum Natura, Artis Magistra”, without more precise locality, without date [but probably 1880s] , [J.C.] Ploen leg., 4 males, 2 females (ZMAN);
“Sumatra, Westkust”, without more precise locality, without date, without collector’s name, 1 male (RMNH);
“Sumatra”, without more precise locality, 1941, without collector’s name, ex coll. K.J.W. Beenet Kempers, 1 female (ZMAN);
“Sumatra”, without more precise locality, 13.v.1983, H. Makihara leg., MN det., 1 spec. (MNIC);
“Sarawak, Borneo”, without more precise locality, without date [but before 1917], without collector’s name, ex coll. A. Grouvelle, 1 male (MNHN);
“Sarawak”, without more precise locality, without date [1854–1862], [A.R.] Wallace leg., 1 male (BMNH);
“S. O. Borneo”, without more precise locality, date and collector’s name, ex coll. B. Schwarzer, 1 male (SMFD);
“Borneo”, without more precise locality, without date [1854–1862], [A.R.] Wallace leg., 1 female (BMNH);
“Borneo”, without more precise locality, date and collector’s name, ex Mus. Westerm.[ann], 1 female (ZMUC);
“Borneo”, without more precise locality, without date [but before 1909], without collector’s name, ex coll. W. Müller, 1 male (SMTD);
“Bornéo”, without date, Montrouvier leg., ex coll. M. Pic, 1 female (MNHN);
“Borneo”, without more precise locality, 1881, without collector’s name, Post II, 1 female (NHMW);
“Java”, without date, coll. Mniszech, ex coll. R. Oberthür, 1 male (MNHN);
“Java”, without more precise locality, date and collector’s name, 1 female (MHNG); same data, 1 male (ZMAS);
“Java”, without more precise locality, without date, Westermann leg., 1 male (ZMUC);
“Java”, without more precise locality, without date, Reinwardt leg., ex Mus. Westerm.[ann], 1 female (ZMUC);
“Java”, without more precise locality, without date, Reinw.[ardt] leg., 1 female (RMNH);
“Java, Museum Natura, Artis Magistra”, without more precise locality, date and collector’s name, 1

male (ZMAN);
“Sulawesi”, without more precise locality, 2002, without collector’s name, WB det., 1 spec. (WBAC);
“Queensland”, without more precise locality and date, Heule leg., ex coll. v. Schönfeld, 1 male, 1 female (SMFD);
“Queensland”, without more precise locality, date and collector’s name, ex coll. B. Schwarzer, 1 female (SMFD);
“Queensland”, without more precise locality, without date, Fristedt leg., 1 male, 1 female (NHRS);
“Q.land”, without more precise locality, without date, Dequet leg., 1 female (CAS);
“N Queensland”, without more precise locality, date and collector’s name, ex coll. A. J. Buis, 3 females (ZMAN);
“N Queensland”, without more precise locality and date, E. Weiske leg., 2 males, 1 female (SMTD);
“North Queensland”, without more precise locality, date and collector’s name, 1 female (AMNH);
“N. Queensland”, without date, ex coll. H. Peters, 3 females (CAS);
“N.S. Wales”, without more precise locality, date and collector’s name, ex coll. H.J. Klaasen, 1 female (ZMAN); same data, 1 female (SMFD);
“Nieuw Zuid Wales” [= New South Wales], without more precise locality, without date, ex coll. H. v.d. Vaart, 2 females (RMNH);
“Australia”, without more precise locality, date and collector’s name, ex coll. Erben, 1 male (NMPC);
“Australia”, without more precise locality and date [probably 1900–1901, Kořenský (1904)], J. Kořenský leg., 1 female (NMPC);
