# Supplementary material for: Integrative taxonomy and species distribution models of the genus Diamesus Hope, 1840 (Coleoptera: Staphylinidae: Silphinae)
Source: Sci Rep. 2023 Feb 23;13:3192. doi: 10.1038/s41598-023-30019-x (PMC9950127; doi:10.1038/s41598-023-30019-x)

SM4: Boxplots representing variability in size or ratios of four major morphological characters between males and females of two studied species, *Diamesus bimaculatus* Portevin, 1914 and *D. osculans* (Vigors, 1825). Horizontal lines within the boxes indicate median values; upper and lower boxes indicate the 75th and 25th percentiles, respectively; whiskers indicate the values with the 1.5 interquartile ranges; small, black dots are outliers.

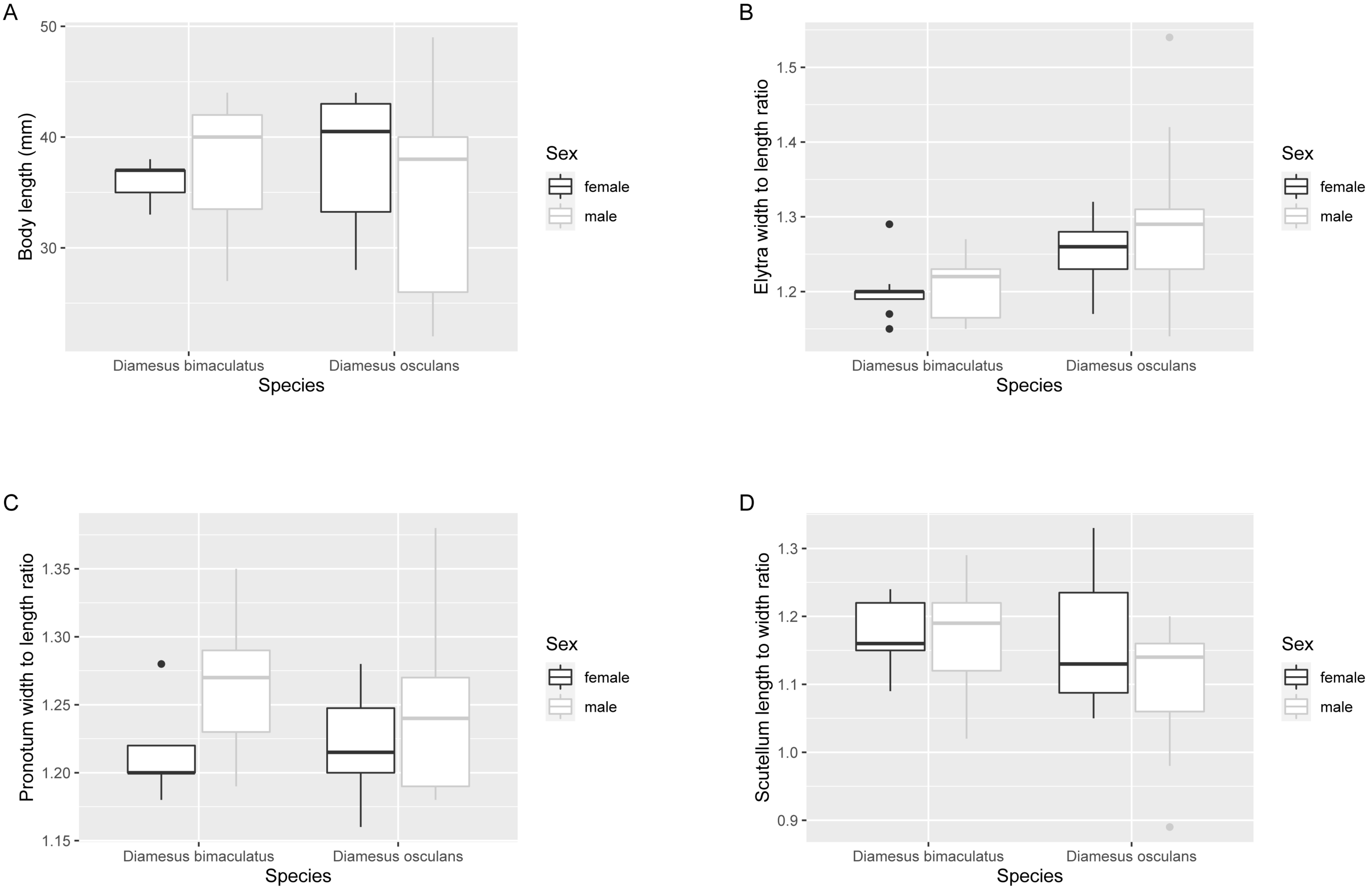

Supplement: Supplementary file 4 — Supplementary Information 4. [file 41598_2023_30019_MOESM4_ESM.pdf]
